# Global burden of hand osteoarthritis in adults aged ≥55 years: An underestimated and fastest-growing subtype, 1990 to 2021 with projections to 2050: an analysis of GBD 2021 data

**DOI:** 10.1097/MD.0000000000050098

**Published:** 2026-08-21

**Authors:** Xiaozong Lin, Wenyan Zhang, Bowen Wu, Mengyao Zhao, Duancen Liu, Maoqing Wang, Dongyu Hou, Jinpeng Zhuang

**Affiliations:** a Department of Orthopaedics, The Second Affiliated Hospital of Harbin Medical University, Harbin, Heilongjiang, China; b National Key Disciplines of Nutrition and Food Hygiene, Department of Nutrition and Food Hygiene, School of Public Health, Harbin Medical University, Harbin, Heilongjiang, China; c Key Laboratory of Precision Nutrition and Health, Ministry of Education, Harbin Medical University, Harbin, Heilongjiang, China.

**Keywords:** adults aged ≥55 years, disease burden, hand osteoarthritis, prevalence, projection

## Abstract

Among osteoarthritis (OA) subtypes, hand osteoarthritis (HOA) is the fastest-growing by estimated annual percentage change in age-standardized rates and percentage change in case numbers. Yet its burden in adults aged ≥55 years, who are disproportionately affected, has not been systematically quantified. Using Global Burden of Disease 2021 data, we assessed the global burden of HOA in this age group. Trends were analyzed using estimated annual percentage change and percentage change, and future projections were made using an autoregressive integrated moving average model. From 1990 to 2021, incident cases, prevalent cases, and years lived with disability (YLDs) in this age group increased by 3.13 million, 95.32 million, and 2.99 million, accounting for 51.40%, 80.80%, and 79.95% of total increases in the general population, respectively. In 2021, there were 5.13 million incident cases, 154.68 million prevalent cases, and 4.86 million YLDs, representing 49.52%, 79.61%, and 78.77% of the global burden, respectively, with corresponding age-standardized rates of 2.89, 4.71, and 4.66 times those in the general population. China, India, the United States, Japan, Russia, and Brazil together accounted for over 54% of incident cases, prevalent cases, and YLDs. By 2050, incident cases, prevalent cases, and YLDs are projected to reach 8.99 million, 292 million, and 8.96 million, respectively, with corresponding age-standardized rates of 2.57, 5.13, and 5.01 times those in the general population. The growth in HOA burden in this age group over the past 3 decades and through 2050 exceeds that of other osteoarthritis subtypes. The burden of HOA in adults aged ≥55 years is disproportionately high and is increasing faster than any other osteoarthritis subtype, with 6 countries (China, India, the United States, Japan, Russia, and Brazil) bearing the heaviest absolute burden. Thus, HOA in this age group has emerged as a global challenge demanding sufficient attention.

## 1. Introduction

Hand osteoarthritis (HOA) is a common subtype of osteoarthritis, characterized by pain, stiffness, swelling, restricted range of motion, and joint deformities in the hand joints.^[1,2]^ These symptoms severely impact patients’ daily activities, often intensifying pain at night, disturbing sleep, and increasing the risk of psychological disorders such as depression and anxiety.^[3–5]^ Compared to knee and hip osteoarthritis, HOA specifically affects fine finger movements, such as dressing, holding chopsticks, and opening bottle caps. It causes instability in holding objects and impairs basic life skills like washing and eating.^[6,7]^ As a result, its impact on daily self-care functions is greater than that of weight-bearing joint arthritis.

According to the 2021 Global Burden of Disease (GBD) data, there were 595 million prevalent cases of osteoarthritis globally in 2020, among which knee osteoarthritis carried the heaviest burden, while HOA ranked 2nd.^[8]^ However, among all osteoarthritis subtypes, HOA is the fastest-growing from 1990 to 2021, measured by estimated annual percentage change (EAPC) in age-standardized rates and percentage change in case numbers.^[8,9]^ Estimates of the global HOA burden in the general population showed that from 1990 to 2021, the number of incident cases increased by 6.09 million, prevalent cases by 117.96 million, and years lived with disability (YLDs) by 3.74 million. By 2021, there were approximately 10.37 million incident cases, 194.28 million prevalent cases, and 6.17 million YLDs globally. It is projected that by 2046, the global incident cases, prevalent cases, and YLDs of HOA will reach 17.66 million, 377.58 million, and 11.86 million, respectively.^[10]^ Critically, the incidence of HOA peaks in the 55 to 59 age group.^[10]^ With the acceleration of global population aging and increased life expectancy, the burden of HOA among older adults is expected to continue rising.^[11,12]^ Moreover, HOA has a more pronounced impact on older adults than on younger populations. Therefore, including younger individuals in all-age estimates leads to rate dilution: it lowers the age-specific rates observed in adults aged ≥55 years because the denominator includes a large number of younger people with a very low disease burden. This dilution may obscure the true high burden in the older age group. Consequently, focusing on adults aged ≥55 years is key to addressing the majority of the global burden of HOA.

Thus, this study utilizes the GBD 2021 database to comprehensively evaluate the temporal trends and current status of HOA disease burden in adults aged ≥55 years from 1990 to 2021 globally, regionally, and nationally, and to forecast the disease burden of HOA in this age group up to 2050, with a focus on incidence, prevalence, and YLDs. We also describe countries with a high absolute disease burden and discuss the underlying reasons. Furthermore, we compare the HOA burden in adults aged ≥55 years with that in the general population and other osteoarthritis subtypes, aiming to provide evidence for priority decision-making in healthcare resource allocation.

## 2. Methods

### 2.1. Study data

Data related to HOA, including the annual incidence, prevalence, and YLDs, were obtained from the 2021 GBD study database using the Global Health Data Exchange (GHDx) query tool. GHDx is a constantly updated website that reflects global and regional epidemiological data. Its data mainly come from the following sources: published reports and systematic reviews, data collected from official and nongovernmental websites, raw data not yet published, and data from GBD collaborators. HOA was defined as symptomatic radiographic osteoarthritis (Kellgren–Lawrence grade 2–4) in any hand joint; International Classification of Diseases-10 code M18 serves as a corresponding mapping code for this definition in coded data sources. To date, a total of 371 diseases in 204 countries and territories have been included in the GBD database to assess their global burden on human health. These 204 countries and territories were divided into 21 regions according to geographic proximity and epidemiological similarity. The sex and age of the patients and country socio-demographic index (SDI) values were also collected to assess their impact on incidence, prevalence, YLDs, and their age-standardized rate. The SDI is a composite measure that ranges from 0 to 1, reflecting the level of social and demographic development. Based on their SDI values, the 204 countries and regions worldwide are categorized into 5 groups: low, low–middle, middle, high–middle, and high SDI. Data on national SDI values were obtained from GHDx (http://ghdx.healthdata.org/gbd-2021/sources).

### 2.2. Statistical analysis

We utilized global, regional, and national data on adults aged ≥55 years with HOA, focusing on incidence, prevalence, and YLDs in terms of cases and rates.

The EAPC is a widely adopted statistical measure used to quantify the annual trends of changes in specific indicators, such as incidence or prevalence rates, over a defined period. It is derived using a linear regression model where the natural logarithm of time-series data is regressed on time to estimate the rate of change, expressed as an annual percentage. The EAPC value reflects the direction and magnitude of a trend: a positive value indicates an upward trend, a negative value suggests a downward trend, and a value close to zero implies minimal or no significant change. EAPC is particularly suitable for long-term trend analysis because it smooths out short-term fluctuations, providing a clearer depiction of overall trends. In epidemiology and public health, particularly within the GBD framework, the EAPC is extensively applied to assess dynamic trends in diseases, injuries, and risk factors.^[13]^

Percentage change is mainly applied to measure the variations in prevalence cases, incidence cases, and YLDs. Specifically, percentage change (%) = ([cases in 2021 − cases in 1990]/cases in 1990) × 100%.^[14]^

To quantify the contributions of demographic and epidemiological factors to changes in the global burden of HOA among adults aged ≥55 years from 1990 to 2021, we conducted a decomposition analysis using the Das Gupta method. This method decomposes the overall change in disease burden into 3 components: population growth, population aging, and epidemiological change. For a given region and year *y*, the total number of cases was expressed as:


$$N_{y}=\underset{k}{\sum }\;\left(a_{k,y}\times p_{y}\times e_{k,y}\right)$$

where *N*_*y*_ is the total number of cases in year *y*; *a*_*k,y*_ is the proportion of the population in age group *k* in year *y*; *p*_*y*_ is the total population in year *y*; and *e*_*k*,*y*_ is the age-specific rate in age group *k* in year *y*. The contribution of each component was estimated by sequentially varying 1 factor while holding the other factors constant.^[15,16]^

The autoregressive integrated moving average (ARIMA) model is a commonly used time-series model for forecasting. In the ARIMA (*p*, *d*, *q*) model, *p* represents the number of autoregressive terms, *d* represents the order of differencing required to achieve stationarity, and *q* represents the number of moving-average terms. In this study, annual data from 1990 to 2021 were used to fit ARIMA models for each projected outcome. The optimal model was selected according to the akaike information criterion and the Bayesian information criterion. Stationarity was assessed using the augmented Dickey–Fuller (ADF) test and the Kwiatkowski–Phillips–Schmidt–Shin (KPSS) test, with ADF *P* values <.05 and KPSS *P* values >.05 indicating acceptable stationarity. Residual autocorrelation was evaluated using the Ljung–Box test, with *P* values >.05 indicating no significant residual autocorrelation. The final ARIMA (*p*, *d*, *q*) parameters, akaike information criterion, Bayesian information criterion, ADF *P* values, KPSS *P* values, and Ljung–Box test *P* values are provided in Supplementary Material. The ARIMA model was then used to forecast the burden of HOA and other osteoarthritis subtypes from 2022 to 2050.^[17,18]^

The data were filtered and calculated using R (version 4.4.3; R Foundation for Statistical Computing). Visualizations were generated using ggplot2 (version 3.5.1; Posit Software, PBC).

### 2.3. Ethics statement

This study used publicly available, de-identified data from the GBD 2021 study. Therefore, institutional review board approval and informed consent were not required.

## 3. Results

### 3.1. Temporal trend of HOA in adults aged ≥55 years from 1990 to 2021

The global incident cases of HOA in this age group increased from 2,008,354 in 1990 to 5,134,309 cases in 2021 – an increase of nearly 3.13 million cases that accounted for 51.40% of the total growth in HOA incidence worldwide. The age-standardized incidence rate (ASIR) increased from 296.13 in 1990 to 344.20 per 100,000 people in 2021, with an EAPC of 0.43 (95% confidence interval [CI]: 0.37, 0.49) (Table S1, Supplemental Digital Content 1, Figs. 1A and 2A and B, Fig. S1, Supplemental Digital Content 2). The prevalent cases increased from 59,357,919 in 1990 to 154,676,897 in 2021 – an increase of nearly 95.32 million cases that accounted for 80.80% of the total growth in HOA prevalence worldwide. The age-standardized prevalence rate (ASPR) rose from 9225.55 in 1990 to 10,549.31 per 100,000 people in 2021, with an EAPC of 0.49 (95% CI: 0.42–0.57) (Table S2, Supplemental Digital Content 3, Figs. 1B and 2A and B, Fig. S1, Supplemental Digital Content 2). The number of YLD increased from 1,872,004 in 1990 to 4,858,626 person-years in 2021 – an increase of nearly 2.99 million person-years that accounted for 79.95% of the total growth in HOA YLD worldwide. The age-standardized YLD rate (ASYR) increased from 289.48 in 1990 to 330.77 per 100,000 people in 2021, with an EAPC of 0.50 (95% CI: 0.42–0.57) (Table S3, Supplemental Digital Content 4, Figs. 1C and 2A and B, Fig. S1, Supplemental Digital Content 2).

**Figure 1.**
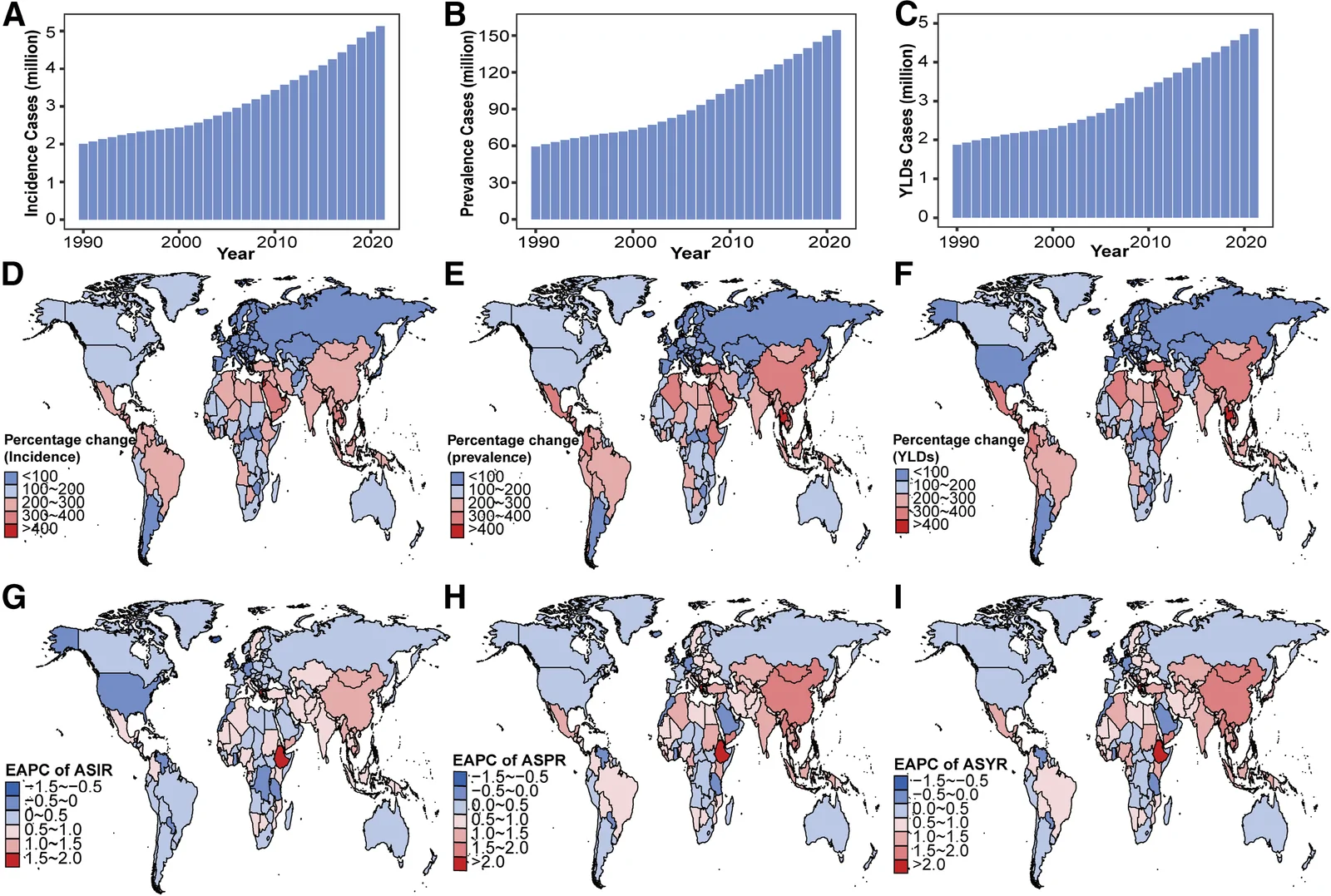
Global temporal trends in the burden of hand osteoarthritis (HOA) in adults aged ≥55 years from 1990 to 2021. (A) Incidence cases, (B) prevalence cases, and (C) YLDs cases. Percentage change: (D) incidence cases, (E) prevalence cases, and (F) YLDs cases. EAPC: (G) ASIR, (H) ASPR, and (I) ASYR. ASIR = age-standardized incidence rate, ASPR = age-standardized prevalence rate, ASR = age-standardized rate, ASYR = age-standardized YLD rate, EAPC = estimated annual percentage change, YLDs = years lived with disability.

**Figure 2.**
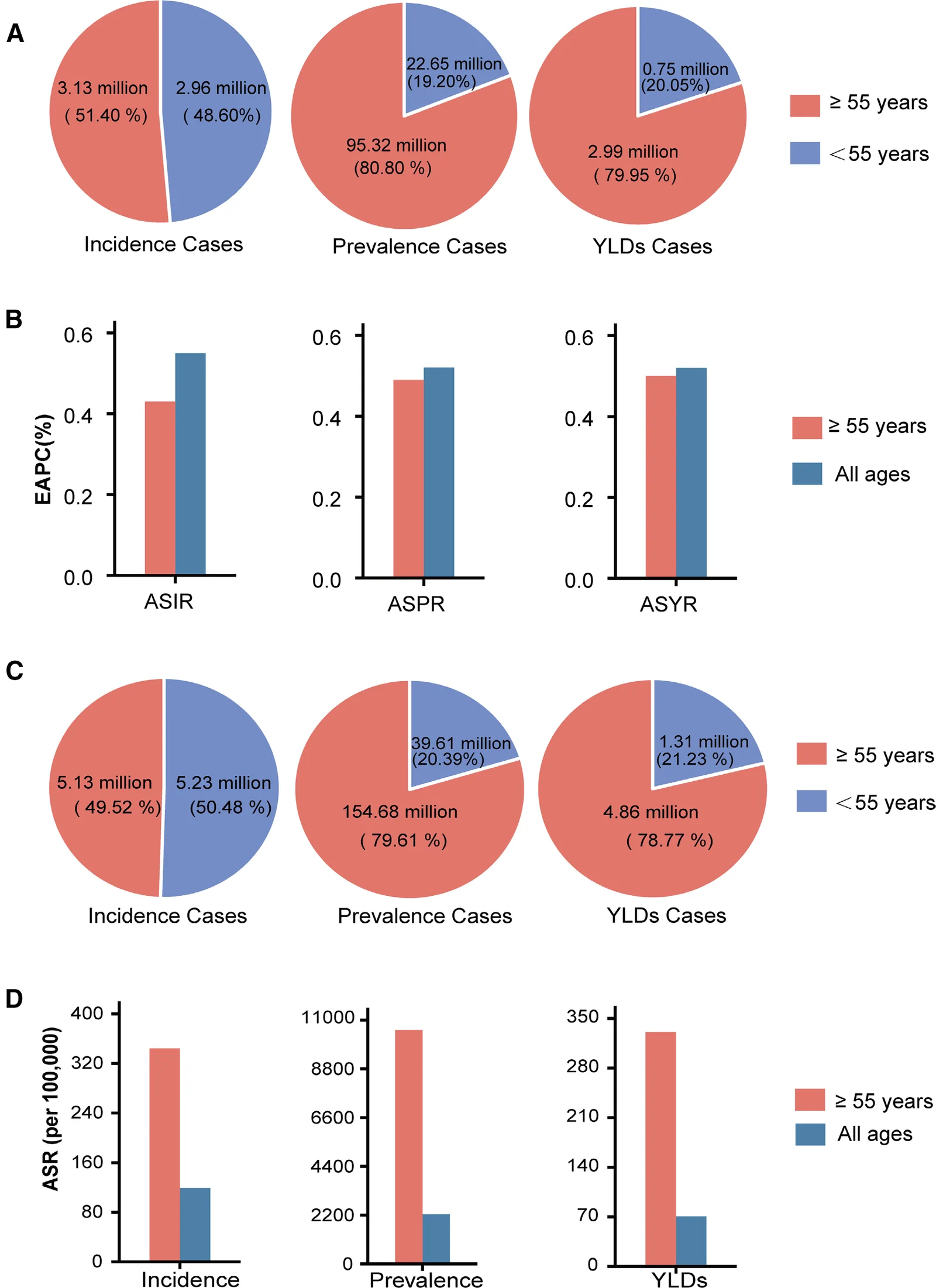
Comparative analysis of the global burden of HOA in adults aged ≥55 years and general population. (A) Change in case numbers, 1990 to 2021, (B) EAPC in age-standardized rates, 1990 to 2021, (C) case numbers in 2021, and (D) age-standardized rates in 2021. ASIR = age-standardized incidence rate, ASPR = age-standardized prevalence rate, ASR = age-standardized rate, ASYR = age-standardized YLD rate, EAPC = estimated annual percentage change, HOA = hand osteoarthritis, YLDs = years lived with disability.

Analyzing by SDI category, the incidence, prevalence, and YLD counts and their corresponding age-standardized rates for HOA in this age group have demonstrated an upward trend across all SDI regions. The middle SDI region experienced the most significant increases in these indicators (Tables S1–S3, Supplemental Digital Content 1, Fig. S1, Supplemental Digital Content 2).

At the national level, an upward trend was observed in the incidence cases, prevalence cases, and YLDs for HOA in this age group across nearly all countries. The United Arab Emirates experienced the fastest growth in incident cases, prevalent cases, and YLDs. Incident cases increased from 147 in 1990 to 2029 in 2021, prevalent cases increased from 4313 to 50,726, and YLDs increased from 136 to 1623, corresponding to percentage changes of 1280.27%, 1076.12%, and 1093.38%, respectively, followed by Qatar and Djibouti (Tables S4–S6, Supplemental Digital Content 5, Fig.1D–F). Among 204 countries, age-standardized rates increased in more than 92% of them. Equatorial Guinea exhibited the most rapid growth in ASIR, ASPR, and ASYR among all countries, with EAPCs of 1.97 (95% CI: 1.84–2.11), 2.69 (95% CI: 2.52–2.86), and 2.74 (95% CI: 2.56–2.92), respectively, followed by Ethiopia and Greece (Tables S4–S6, Supplemental Digital Content 5, Fig. 1G–I).

In terms of gender and age, new cases increased by 2 million in females and 1.1 million in males. Prevalent cases rose by 63.22 million in females and 32.09 million in males. YLDs increased by 2 million in females and 1 million in males. The ASIR, ASPR, and ASYR increased more sharply in females than in males. The highest disease burden growth was observed in the 55 to 59 age group (Tables S1–S3, Supplemental Digital Content 1, Fig. S2, Supplemental Digital Content 12).

### 3.2. Global burden of HOA in adults aged ≥55 years in 2021

GBD 2021 estimated that, globally, approximately 5.13 million incident cases, 154.68 million prevalent cases, and 4.86 million YLDs of HOA occurred in this age group, accounting for about 49.52%, 79.61%, and 78.77% of the total HOA burden, respectively. The age-standardized incidence, prevalence, and YLD rates of HOA in adults aged ≥55 years were 344.20, 10,549.31, and 330.77 per 100,000 population, respectively. These rates were 2.89, 4.71, and 4.66 times higher than the corresponding rates in the general population, which were 119.22, 2240.15, and 71.01 per 100,000 population, respectively (Fig. 2C and D).

Among the 5 SDI regions, the Middle SDI region reported the highest number of incidence cases, while the High SDI region had the highest number of prevalence cases and YLDs (Tables S1–S3, Supplemental Digital Content 1). The High SDI region had the highest ASIR, ASPR, and ASYR, followed by the High-middle SDI region (Fig. S1, Supplemental Digital Content 2).

In 2021, the top 6 countries with the highest incidence, prevalence, and YLD counts were China, India, the USA, Japan, Russia, and Brazil, in that order. The incidence counts for these countries were 1,148,737; 711,192; 464,284; 216,973; 212,511; and 179,648, respectively, with a total of 2,933,345 cases accounting for 57.13% of the global incidence total. The prevalence counts for these countries were 27,880,054; 19,307,442; 15,443,788; 8,586,002; 8,312,532; and 5,264,023, respectively, with a total of 84,793,841 cases accounting for 54.82% of the global prevalence total. The YLD counts for these countries were 885,231; 598,460; 477,385; 270,876; 260,104; and 164,085, respectively, with a total of 2,656,141 YLDs accounting for 54.67% of the global YLD total (Table 1, Tables S4–S6, Supplemental Digital Content 5, Fig. 3A–C). Russia had the highest ASIR (505.49 per 100,000 people), followed by Kazakhstan and Mongolia. Kazakhstan has the highest ASPR and ASYR, with ASPR at 21,580.69 per 100,000 people (95% uncertainty interval: 15,468.15–26,152.67) and ASYR at 678.48 per 100,000 people (95% uncertainty interval: 290.68–1341.25), followed by Mongolia and Russia (Tables S4–S6, Supplemental Digital Content 5, Fig. 3D–F).

**Table 1:** Disease burden and risk factors of HOA among adults aged ≥55 years in 6 high-burden countries, 2021.

Measure	Dimension	China	India	USA	Japan	Russia	Brazil
Incidence	Number_2021 (95% UI)	1,148,737 (778,955–1,643,125)	711,192 (490,682–1,026,672)	464,284 (324,079–665,294)	216,973 (156,839–300,114)	212,511 (145,399–309,742)	179,648 (123,421–259,267)
Incidence	ASR_2021 (95% CI)	305.02 (304.45–305.59)	350.21 (349.38–351.05)	479.25 (477.86–480.64)	470.46 (468.32–472.61)	505.49 (503.31–507.67)	410.62 (408.72–412.53)
Prevalence	Number_2021 (95% UI)	27,880,054 (20,121,293–36,885,010)	19,307,442 (14,050,518–25,288,127)	15,443,788 (11,355,709–19,850,168)	8,586,002 (6,440,704–10,949,904)	8,312,532 (6,153,567–10,693,606)	5,264,023 (3,856,447–6,840,527)
Prevalence	ASR_2021 (95% CI)	7643.31 (7640.41–7646.21)	10,021.77 (10,017.15–10,026.38)	15,031.10 (15,023.56–15,038.64)	14,515.91 (14,505.17–14,526.65)	19,492.71 (19,479.20–19,506.23)	12,355.43 (12,344.85–12,366.02)
YLDs	Number_2021 (95% UI)	885,231 (405,688–1,832,709)	598,460 (274,786–1,238,571)	477,385 (220,732–981,165)	270,876 (124,974–553,427)	260,104 (119,578–541,913)	164,085 (75,222–337,729)
YLDs	ASR_2021 (95% CI)	241.47 (240.95–241.98)	308.50 (307.70–309.31)	465.79 (464.46–467.12)	464.43 (462.50–466.37)	609.65 (607.26–612.04)	384.43 (382.57–386.30)
Risk factor	Demographic drivers	Accelerated aging	Large elderly population base	Large elderly population base	World’s most aged society; Longevity society	Accelerated aging	Accelerated aging
Risk factor	Behavioral/cultural factors	Agricultural/manual labor	Agricultural/manual labor; vegetarian diet	Opioid abuse risk	Precision manual work	High-salt diet; alcohol abuse	Agricultural/manual labor; delayed medical consultation
Risk factor	Metabolic factors	High prevalence of hyperuricemia/gout; diabetes	Vitamin D deficiency	High obesity rate	—	Obesity and metabolic syndrome	Rapidly rising obesity rate
Risk factor	Environmental factors	—	Hot and humid climate	—	—	Long-term cold exposure	Chikungunya infection

ASR = age-standardized rate, CI = confidence interval, HOA = hand osteoarthritis, YLDs = years lived with disability, UI = uncertainty interval, USA = United States of America.

“—” indicates not applicable.

**Figure 3.**
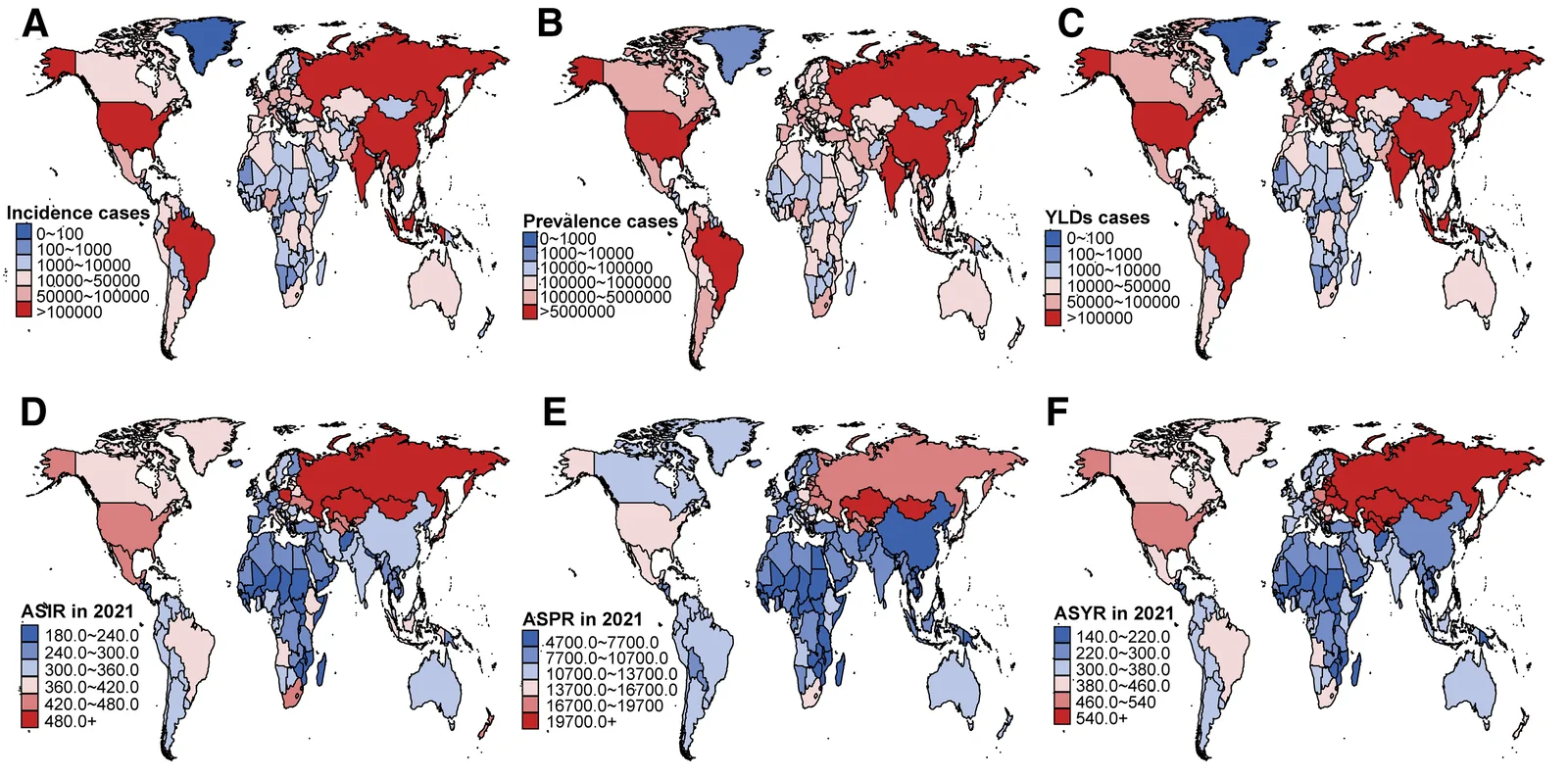
The burden of HOA in adults aged ≥55 years in 204 countries in 2021. (A) Incidence cases, (B) prevalence cases, (C) YLDs cases, (D) age-standardized incidence rate, (E) age-standardized prevalence rate, and (F) age-standardized YLD rate. ASIR = age-standardized incidence rate, ASPR = age-standardized prevalence rate, ASR = age-standardized rate, ASYR = age-standardized YLD rate, EAPC = estimated annual percentage change, HOA = hand osteoarthritis, YLDs = years lived with disability.

In terms of age and gender, the burden of HOA among older women was higher than that among older men. The incidence cases of HOA gradually decreased with age. The ASIR peaked in the 55 to 59 age group, decreased progressively until the 75 to 79 age group, and then rose again. The prevalence cases increased with age, peaked in the 65 to 69 age group, and then showed a downward trend until the oldest age group. The ASPR increased with age. The trend of YLD was the same as the trend of prevalence (Fig. S3, Supplemental Digital Content 13).

Decomposition analysis showed that in 2021, population growth was the primary driver of the incidence, prevalence, and YLD burden of HOA in this age group globally and across the 5 SDI regions, followed by epidemiological change and population aging (Table S7, Supplemental Digital Content 6, Fig. S4, Supplemental Digital Content 14).

### 3.3. Projection

By 2050, the global burden of HOA in adults aged ≥55 years is projected to reach 8.99 million incident cases, 291.92 million prevalent cases, and 8.96 million YLDs, representing increases of 75.12%, 88.73%, and 84.51%, respectively, compared to 2021 (Table S8, Supplemental Digital Content 7). The age-standardized incidence, prevalence, and YLD rates of HOA among adults aged ≥55 years are projected to reach 402.38, 12,082.39, and 373.62 per 100,000 population, respectively. These rates are projected to be 2.57, 5.13, and 5.01 times the corresponding rates in the general population, which are 156.38, 2352.51, and 74.54 per 100,000 population, respectively (Table S8, Supplemental Digital Content 7, Fig. 4).

**Figure 4.**
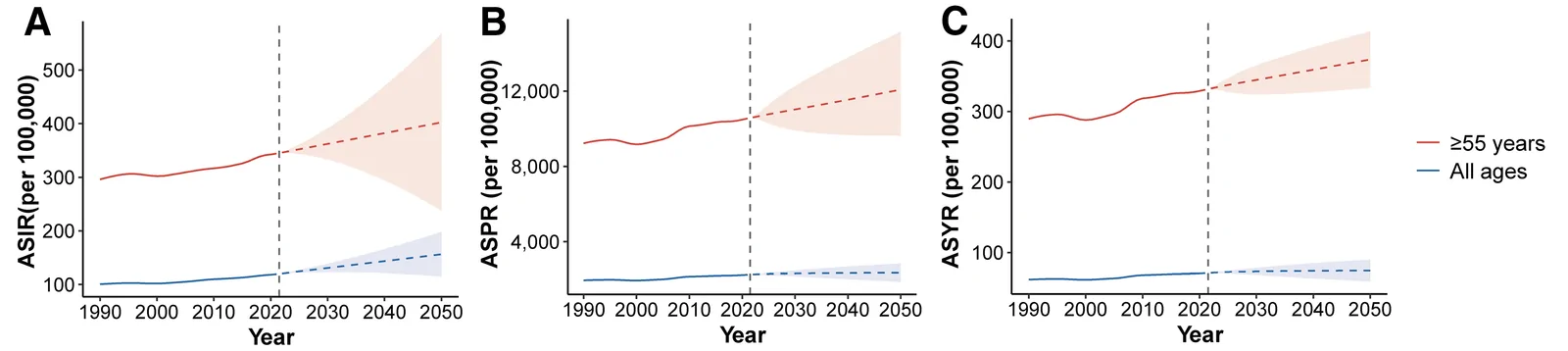
Comparative analysis of the projected global burden of HOA in adults aged ≥ 55 years and general population from 2022 to 2050. (A) Age-standardized incidence rate, (B) age-standardized prevalence rate, and (C) age-standardized YLD rate. ASIR = age-standardized incidence rate, ASPR = age-standardized prevalence rate, ASYR = age-standardized YLD rate, HOA = hand osteoarthritis, YLDs = years lived with disability.

### 3.4. Comparative analysis of the global burden of HOA and other OA in adults aged ≥55 years

**Figure 5.**
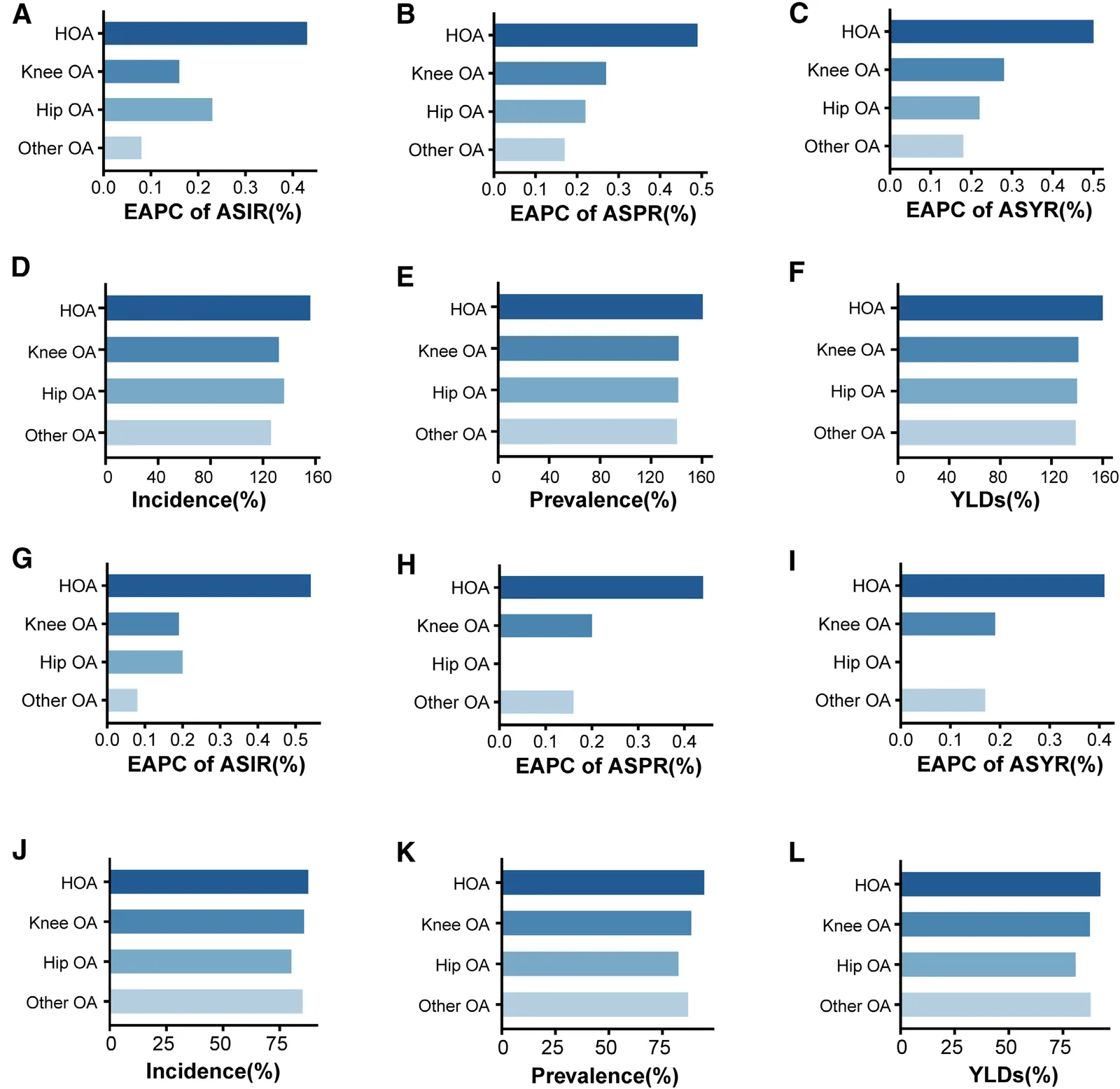
Comparative analysis of the global burden of HOA and other OA in adults aged ≥55 years. (A–C) EAPC from 1990 to 2021; (D–F) percentage change in case numbers, 2021 versus 1990; (G–I) EAPC from 2022 to 2050; and (J–L) percentage change in case numbers, 2050 versus 2021. ASIR = age-standardized incidence rate, ASPR = age-standardized prevalence rate, ASYR = age-standardized YLD rate, EAPC = estimated annual percentage change, HOA = hand osteoarthritis, OA = osteoarthritis, YLDs = years lived with disability.

As shown in Figure 5A–F, among adults aged ≥55 years, HOA exhibited the highest EAPCs in age-standardized rates and the largest percentage changes in case numbers from 1990 to 2021 compared with all other OA subtypes (Tables S9 and S10, Supplemental Digital Contents 8, Fig. 5A–F).

Although the overall burden of HOA remains lower than that of knee OA in both 2021 and 2050, projections indicate an important shift by 2050: both the EAPCs of age-standardized rates and the percentage increases in case numbers are higher for HOA than for other OA subtypes (Fig. 5G–L, Fig. S5, Supplemental Digital Content 15). This consistently steeper growth trajectory, observed both historically and in projections, highlights HOA as an increasingly important contributor to the overall OA burden, warranting targeted attention.

## 4. Discussion

While previous research has documented a growing global burden of HOA in the general population from 1990 to 2021, no study has specifically examined trends among older adults.^[8,10]^ This study offers the 1st comprehensive assessment of the global burden of HOA in adults aged ≥55 years. From 1990 to 2021, the increases in incident cases (3.13 million), prevalent cases (95.32 million), and YLDs (2.99 million person-years) in this age group accounted for 51.40%, 80.80%, and 79.95%, respectively, of the total rise in HOA burden worldwide. In 2021, this age group represented 49.52% of incident HOA cases, 79.61% of prevalent cases, and 78.77% of HOA-attributable YLDs. Their age-standardized incidence, prevalence, and YLD rates were 2.9 to 4.7 times higher than those in the general population. Countries with the highest absolute numbers of incident cases, prevalent cases, and YLDs included China, India, the USA, Japan, Russia, and Brazil. Earlier projections estimated a 48.6% increase in prevalent HOA cases in the general population by 2050.^[8]^ In contrast, our analysis suggests a substantially greater increase of 88.73% in adults aged ≥55 years. We further provide the 1st estimates indicating that incident cases and YLDs in this age group will rise by 75% and 85%, respectively. Moreover, age-standardized incidence, prevalence, and YLD rates in this age group are projected to be 2.57, 5.13, and 5.01 times those in the general population, respectively, by 2050. Notably, although the burden of HOA remained lower than that of knee OA between 1990 and 2050, its growth rate exceeded that of all other OA subtypes. These findings underscore that adults aged ≥55 years bear a disproportionately large and rapidly growing share of the global HOA burden, with a growth rate surpassing all other OA subtypes. Addressing HOA prevention and management in this population, therefore, represents a pressing public health challenge.

Global population aging is occurring concurrently with increased life expectancy, resulting in a steady rise among older adults.^[9,19]^ Our decomposition analysis reveals that 88.31% of the global YLD attributable to HOA in adults aged≥ 55 years in 2021 can be attributed to population growth. Apart from demographic factors, the rising prevalence of HOA in this age group may be associated with various factors, including economic development, metabolic factors, behavioral and cultural factors, ecological environments, and the availability and quality of healthcare infrastructure.^[20–23]^

This study confirmed a significantly higher burden of HOA in older females compared to males, in line with previous studies.^[8,19]^ Across different age groups, we observed that ASIR peaks in the 55 to 59 age group, gradually declines until the 75 to 79 age group, and then increases again. The peak in the 55 to 59 group is attributed to higher physical demands from work and daily activities during this period.^[24]^ After age 60, reduced physical activity following retirement leads to decreased joint loading and a corresponding decline in HOA incidence. However, beyond age 79, the rate increases again, likely due to age-related joint degeneration, accumulated pathological changes, and reduced compensatory capacity.^[11,25]^

Our study reveals that while the burden of HOA in this age group is currently relatively low in low to middle SDI regions, it is increasing rapidly. This trend is driven by high rates of physically demanding work in sectors like manufacturing and agriculture, where repetitive hand use and inadequate joint protection accelerate cartilage damage and osteophyte formation. These challenges are compounded by limited healthcare resources and underdeveloped diagnostic systems.^[26,27]^ In contrast, high and high-middle SDI regions have the highest HOA burden but the slowest growth, due to automation reducing manual labor and stronger healthcare systems controlling disease progression.^[9,28]^ Thus, a dual strategy is essential: high-SDI regions should focus on treatment and rehabilitation to reduce disability, while lower-SDI regions need preventive measures to curb incidence and spread.

In 2021, China, India, the United States, Japan, Russia, and Brazil were the leading countries in terms of incident cases, prevalent cases, and YLDs. Based on previous literature, we hypothesize that the following factors may contribute to the high burden in these countries. In China, the high prevalence of HOA in adults aged ≥55 years may be linked to accelerated population aging, mechanical stress from agricultural and manual labor, a high prevalence of metabolic factors such as hyperuricemia/gout and diabetes, coupled with systemic under-recognition of HOA and limited primary care management (Table 1).^[29–32]^ In India, the high burden of HOA among older adults may be linked to a large aging population, occupational exposure to agricultural and manual labor, potential nutritional deficiencies (e.g., Vitamin D deficiency), and unequal healthcare access.^[33–36]^ Obesity is positively correlated with HOA; a 10-unit increase in body mass index is associated with a 56% increase in OA risk among women.^[21–23]^ In the USA, the persistent issue of obesity, with a prevalence of about 35% among older adults, may be a plausible contributor to the HOA burden.^[37]^ Japan currently exhibits the highest level of population aging globally. Occupational factors and cultural lifestyle practices, such as cooking and fine manual work, may be associated with increased strain on hand joints.^[24,38]^ In Russia, extreme climatic conditions may worsen joint stiffness and pain in arthritis patients.^[39,40]^ Moreover, high intakes of salt, fat, and alcohol may contribute to systemic inflammation and cartilage damage.^[41]^ In Brazil, the burden of HOA may be associated with a confluence of factors, including rapid population aging amid regional development disparities (the “aging-before-prosperity” phenomenon), extreme health inequities, prior chikungunya infection, and the obesity epidemic.^[42–44]^ In summary, different countries face unique risk factors for HOA, and the high burden of HOA may result from the combined effects of diverse demographic structures, behavioral and cultural factors, metabolic factors, healthcare accessibility, and specific regional environmental and socioeconomic conditions.

Our study found that Kazakhstan had the highest point estimates of ASPR and ASYR for HOA in adults aged ≥55 years globally. However, this finding should be interpreted with caution. Taking ASYR as an example, its 95% uncertainty interval is very wide, with the upper bound approximately 4.6 times the lower bound (290.68–1341.25 per 100,000), indicating substantial model‑driven imprecision. Moreover, there is currently a lack of high‑quality primary epidemiological data on HOA in Kazakhstan. Based on these 2 observations, we speculate that Kazakhstan’s high age‑standardized rates may primarily reflect data scarcity and extrapolation uncertainty in the GBD modeling framework, rather than definitively representing a truly higher burden than all other countries. Of course, the possibility of true epidemiological differences cannot be completely ruled out.^[45,46]^ Therefore, we recommend cautious interpretation of this point estimate ranking and encourage population‑based primary studies in Kazakhstan to validate the GBD modeled estimates.

By 2050, we project 292 million prevalent HOA cases in adults aged ≥55 years, representing an 88.73% increase from 2021. This increase is substantially greater than the 48.6% rise in all-age HOA prevalence reported in the GBD 2021 study. Furthermore, age-standardized incidence, prevalence, and YLD rates are expected to remain markedly higher in this age group than in the general population, at approximately 2.57, 5.13, and 5.01 times, respectively. These findings highlight the necessity of age-stratified analyses, as focusing on the general population may mask the escalating burden in the older age group and lead to an underestimation of future healthcare needs. From a health policy perspective, 292 million affected elderly individuals by 2050 implies a surge in demand for hand surgery, occupational therapy, pain management, and assistive devices for daily living. These projections underscore the need to strengthen healthcare capacity for HOA, particularly in countries with rapidly aging populations or limited primary care infrastructure.

Previous general-population projections estimated a 48.6% increase in prevalent HOA cases by 2050, lower than those for knee (74.9%), hip (78.6%), and other OA (95.1%)^[8]^; in contrast, our age-stratified analysis indicates an 88.73% increase in prevalent cases in adults aged ≥55 years, with both case growth and EAPC exceeding those of all other OA subtypes. Thus, our study reveals a critical disparity: although the burden of HOA has remained lower than that of knee OA from 1990 to 2050, its growth rate exceeds that of all other OA subtypes. This divergence stems from knee OA’s larger baseline burden, amplified by escalating global overweight prevalence and sports-related injuries that sustain high levels of weight-bearing joint damage.^[47,48]^ Conversely, HOA’s “small baseline yet rapid growth” pattern underscores its insidious emergence as a concealed health threat, with chronic neglect and underdiagnosis substantially underestimating its true burden. This accelerated trajectory is driven by several HOA-specific factors. First, metabolic dysregulation – manifested through the rising prevalence of hyperuricemia, type 2 diabetes, and obesity – disproportionately affects hand joints via systemic inflammation and advanced glycation end-product accumulation in cartilage.^[29–31]^ Second, despite occupational ergonomic improvements reducing knee and hip injuries, labor-intensive manual work persists unrecognized in many regions, chronically exposing aging workers to repetitive hand strain.^[10,49]^ Third, HOA is frequently overlooked in primary care, where inadequate screening leads to diagnostic delays and case concentration among severely affected elderly patients, contrasting sharply with earlier knee OA identification and intervention.^[1,3,10]^ Furthermore, the hand’s multi-joint architecture confers higher cumulative susceptibility, while pronounced female predominance intersects with global population aging among women and prolonged postmenopausal estrogen deficiency to amplify disease expansion.^[1,19]^ These distinct drivers demonstrate that generic OA prevention strategies are inadequate for hand disease, necessitating urgent targeted interventions focusing on metabolic risk modulation, enhanced occupational hand protection, and improved diagnostic capacity in primary healthcare systems.

Future strategies should focus on the following key areas. Early screening programs: prioritize early screening programs for older women in high-risk countries.^[50]^ Occupational protection: provide occupational protection training and appropriate equipment to elderly workers in jobs that involve high hand use to reduce long-term joint injuries.^[51]^ Hand function rehabilitation: integrate hand function rehabilitation into primary healthcare to address disability caused by HOA.^[51]^ Personalized treatment plans: develop personalized treatment plans tailored to country-specific etiologies, integrating pharmacological therapy, physical therapy, and rehabilitation exercises to improve patient outcomes.^[52]^ By addressing these areas, we can better manage the growing burden of HOA and improve the quality of life for affected individuals.

## 5. Limitations

Our study has several limitations that warrant acknowledgment. First, data were sourced from multiple countries and territories, each employing distinct diagnostic criteria, screening protocols, and surveillance systems for HOA. Second, the limited availability of data from certain regions or time periods hindered a more accurate depiction of the intra-country epidemiological landscape. Third, it does not account for specific risk factors associated with HOA, such as obesity and occupational exposure. The absence of such data limits the potential for formulating targeted preventive and intervention strategies. Fourth, the ARIMA prediction is based on the continuation of past epidemiological patterns and does not take into account changes that may be brought about by preventive interventions, improvements in diagnostic techniques, or emerging therapeutic strategies. To quantify forecast uncertainty, the relative widths of the 2050 95% prediction intervals for incident cases, prevalent cases, YLDs, and their age‑standardized rates ranged from 21.3% to 90.5% of the respective point estimates among adults aged ≥55 years. Finally, it does not delineate the specific subtypes of HOA.

## 6. Conclusions

We present a comprehensive assessment of the global burden of HOA in adults aged ≥55 years. This age group accounts for a disproportionately high and steadily increasing share of cases, particularly in China, India, the United States, Japan, Russia, and Brazil. Our analysis reveals that over the past 3 decades, the burden of HOA has increased at a faster rate than that of any other osteoarthritis subtype, and projections indicate that this trend will persist through 2050. Taken together, these findings underscore the urgent need for prioritized public health interventions – including targeted prevention, early diagnosis, and effective management – to mitigate the future impact of HOA.

## Acknowledgments

We thank the Institute for Health Metrics and Evaluation staff and its collaborators who prepared these publicly available data.

## Author contributions

**Conceptualization:** Xiaozong Lin, Wenyan Zhang, Bowen Wu.

**Data curation:** Bowen Wu, Mengyao Zhao, Duancen Liu.

**Formal analysis:** Xiaozong Lin, Wenyan Zhang, Jinpeng Zhuang.

**Funding acquisition:** Jinpeng Zhuang.

**Investigation:** Xiaozong Lin, Wenyan Zhang, Bowen Wu, Mengyao Zhao, Duancen Liu, Maoqing Wang, Dongyu Hou, Jinpeng Zhuang.

**Methodology:** Xiaozong Lin, Wenyan Zhang, Bowen Wu, Maoqing Wang, Dongyu Hou, Jinpeng Zhuang.

**Project administration:** Xiaozong Lin, Wenyan Zhang, Maoqing Wang, Dongyu Hou, Jinpeng Zhuang.

**Resources:** Mengyao Zhao, Duancen Liu, Dongyu Hou.

**Software:** Mengyao Zhao, Duancen Liu, Jinpeng Zhuang.

**Supervision:** Maoqing Wang, Dongyu Hou, Jinpeng Zhuang.

**Validation:** Xiaozong Lin, Wenyan Zhang, Bowen Wu, Mengyao Zhao, Duancen Liu, Maoqing Wang, Dongyu Hou, Jinpeng Zhuang.

**Visualization:** Xiaozong Lin, Wenyan Zhang, Bowen Wu, Mengyao Zhao, Duancen Liu, Maoqing Wang, Dongyu Hou, Jinpeng Zhuang.

**Writing – original draft:** Xiaozong Lin, Wenyan Zhang, Bowen Wu.

**Writing – review & editing:** Xiaozong Lin, Wenyan Zhang, Bowen Wu, Mengyao Zhao, Duancen Liu, Maoqing Wang, Dongyu Hou, Jinpeng Zhuang.






























