# Supplementary material for: Global burden of hand osteoarthritis in adults aged ≥55 years: An underestimated and fastest-growing subtype, 1990 to 2021 with projections to 2050: an analysis of GBD 2021 data
Source: Medicine (Baltimore). 2026 Aug 21;105(34):e50098. doi: 10.1097/MD.0000000000050098 (PMC13502859; doi:10.1097/MD.0000000000050098)
Supplement: Supplementary file 1 [file medi-105-e50098-s001.docx]

**Table S1. The incidence of HOA cases and rate in adults aged ≥55 years in 1990 and 2021, and the trends from 1990 to 2021.**

| **location** | **Incidence Cases** | | **Percentage change**  **(100%)** | **ASIR** | | **EAPC (95%CI)** |
| --- | --- | --- | --- | --- | --- | --- |
|  | **1990 (95% UI)** | **2021 (95% UI)** |  | **1990(95%CI)** | **2021(95%CI)** |  |
| Global | 2008354 (1368574,2926073) | 5134309 (3514305,7415103) | 155.65 | 296.13 (295.71,296.54) | 344.20(343.90,344.50) | 0.43 (0.37,0.49) |
| Female | 1307266 (897389,1885862) | 3294824 (2273314,4721186) | 152.03 | 363.01 (362.39,363.64) | 420.25(419.80,420.71) | 0.51 (0.44,0.58) |
| Male | 701088 (469813,1034649) | 1839485 (1237245,2692393) | 162.38 | 219.07 (218.54,219.61) | 259.42(259.04,259.80) | 0.32 (0.22,0.43) |
| High SDI | 686002 (476484,993167) | 1328181 (922412,1904246) | 93.61 | 376.81 (375.92,377.71) | 403.48(402.78,404.17) | 0.14 (0.04,0.23) |
| High-middle SDI | 547067 (368815,802015) | 1218411 (824170,1763156) | 122.72 | 313.42 (312.57,314.27) | 351.62(350.99,352.25) | 0.3 (0.2,0.4) |
| Middle SDI | 448069 (299827,653208) | 1627474 (1108666,2365460) | 263.22 | 256.66 (255.86,257.46) | 341.92(341.39,342.45) | 0.87 (0.8,0.94) |
| Low-middle SDI | 244816 (165487,354444) | 739655 (502311,1063517) | 202.13 | 241.76 (240.75,242.77) | 303.29(302.59,304.00) | 0.73 (0.68,0.78) |
| Low SDI | 79883 (53819,117128) | 216133 (145938,312082) | 170.56 | 214.86 (213.22,216.52) | 260.69(259.53,261.85) | 0.64 (0.6,0.67) |
| Andean Latin America | 10519 (7004,15046) | 33305 (22405,48533) | 216.62 | 304.35 (298.53,310.29) | 331.64(328.09,335.23) | 0.24 (0.17,0.31) |
| Australasia | 13076 (8919,18859) | 30087 (20739,43737) | 130.09 | 342.68 (336.78,348.68) | 357.42(353.36,361.53) | 0.12 (0.11,0.14) |
| Caribbean | 12251 (8249,17676) | 29417 (19990,42655) | 140.12 | 285.29 (280.21,290.50) | 314.69(311.10,318.31) | 0.32 (0.32,0.33) |
| Central Asia | 32650 (21949,49574) | 73123 (49234,107473) | 123.96 | 384.56 (380.33,388.85) | 449.37(446.03,452.74) | 0.56 (0.51,0.6) |
| Central Europe | 99022 (67756,144120) | 143924 (99336,208480) | 45.35 | 368.39 (366.05,370.75) | 410.36(408.21,412.53) | 0.39 (0.37,0.42) |
| Central Latin America | 43167 (28960,62507) | 160440 (108666,235710) | 271.67 | 310.73 (307.77,313.73) | 368.06(366.25,369.87) | 0.58 (0.57,0.59) |
| Central Sub-Saharan Africa | 10939 (7331,16750) | 28541 (19215,42592) | 160.91 | 275.19 (269.26,281.45) | 297.09(293.31,300.97) | 0.19 (0.1,0.27) |
| East Asia | 302242 (198996,442699) | 1187819 (806114,1698658) | 293.00 | 209.56 (208.70,210.44) | 304.54(303.99,305.10) | 1.03 (0.84,1.23) |
| Eastern Europe | 222540 (149971,324468) | 298126 (204222,431272) | 33.97 | 449.93 (448.02,451.85) | 486.75(484.99,488.52) | 0.29 (0.27,0.31) |
| Eastern Sub-Saharan Africa | 27888 (18769,40909) | 82950 (55839,119772) | 197.44 | 228.60 (225.65,231.64) | 299.65(297.51,301.81) | 0.9 (0.88,0.92) |
| High-income Asia Pacific | 152555 (106434,220143) | 287269 (202969,405927) | 88.31 | 431.20 (429.03,433.40) | 444.92(443.22,446.62) | 0.3 (0.06,0.53) |
| High-income North America | 242341 (167293,351181) | 508012 (355069,729238) | 109.63 | 441.99 (440.21,443.78) | 467.66(466.37,468.96) | -0.06 (-0.33,0.22) |
| North Africa and Middle East | 62833 (41938,92529) | 213308 (142536,313127) | 239.48 | 218.21 (216.43,220.00) | 270.72(269.55,271.90) | 0.67 (0.58,0.76) |
| Oceania | 1114 (741,1613) | 3510 (2382,5201) | 215.08 | 225.42 (210.39,243.12) | 270.40(260.84,280.46) | 0.59 (0.58,0.61) |
| South Asia | 251420 (169230,367212) | 855397 (587734,1242521) | 240.23 | 261.97 (260.87,263.07) | 340.39(339.65,341.13) | 0.86 (0.82,0.9) |
| Southeast Asia | 104390 (70032,152870) | 382145 (258181,552808) | 266.07 | 244.59 (243.05,246.15) | 324.49(323.43,325.55) | 0.87 (0.85,0.9) |
| Southern Latin America | 26646 (17883,39219) | 50285 (33883,73255) | 88.72 | 335.58 (331.54,339.67) | 348.83(345.78,351.90) | 0.08 (0.07,0.1) |
| Southern Sub-Saharan Africa | 17223 (11716,24950) | 43143 (29350,61795) | 150.50 | 383.13 (377.35,389.03) | 431.54(427.36,435.80) | 0.38 (0.36,0.41) |
| Tropical Latin America | 54782 (37193,78769) | 182823 (125700,263759) | 233.73 | 356.42 (353.37,359.51) | 408.44(406.57,410.32) | 0.43 (0.4,0.46) |
| Western Europe | 285350 (196565,408919) | 444130 (305219,635376) | 55.64 | 304.89 (303.76,306.02) | 315.41(314.47,316.36) | 0.07 (0.03,0.1) |
| Western Sub-Saharan Africa | 35406 (23846,51905) | 96555 (65056,142120) | 172.71 | 246.02 (243.35,248.74) | 291.67(289.75,293.61) | 0.66 (0.55,0.77) |
