# Supplementary material for: Global burden of hand osteoarthritis in adults aged ≥55 years: An underestimated and fastest-growing subtype, 1990 to 2021 with projections to 2050: an analysis of GBD 2021 data
Source: Medicine (Baltimore). 2026 Aug 21;105(34):e50098. doi: 10.1097/MD.0000000000050098 (PMC13502859; doi:10.1097/MD.0000000000050098)
Supplement: Supplementary file 2 [file medi-105-e50098-s002.docx]

**Table S2. The Prevalence of HOA cases and rate in adults aged ≥55 years in 1990 and 2021, and the trends from 1990 to 2021.**

| **location** | **Prevalence cases** | | **Percentage change**  **(100%)** | **ASPR** | | **EAPC (95%CI)** |
| --- | --- | --- | --- | --- | --- | --- |
|  | **1990 (95% UI)** | **2021 (95% UI)** |  | **1990(95%CI)** | **2021(95%CI)** |  |
| Global | 59357919 (43382064,77437231) | 154676897 (113502928,201841628) | 160.58 | 9225.55 (9223.14,9227.96) | 10549.31(10547.64,10550.98) | 0.49 (0.42,0.57) |
| Female | 40633478 (30012110,52699944) | 103857694(77009822,134542545) | 155.6 | 11446.46 (11442.91,11450.01) | 13167.61(13165.08,13170.15) | 0.6 (0.49,0.71) |
| Male | 18724441 (13471418,24862198) | 50819202(36669752,66953901) | 171.41 | 6443.47 (6440.35,6446.59) | 7494.59(7492.50,7496.68) | 0.37 (0.27,0.47) |
| High SDI | 22487049 (16570249,28981723) | 46797396 (34555051,60083116) | 108.11 | 11872.32 (11867.38,11877.26) | 12998.45(12994.68,13002.23) | 0.42 (0.23,0.62) |
| High-middle SDI | 17835437 (13194527,23218826) | 38308844 (28319851,50179092) | 114.79 | 10722.28 (10717.13,10727.44) | 11121.33(11117.80,11124.86) | 0.2 (0.1,0.29) |
| Middle SDI | 11102888 (7950273,14775562) | 44351829 (32449213,58020651) | 299.46 | 6945.23 (6940.82,6949.65) | 9807.47(9804.54,9810.41) | 1.12 (1.06,1.18) |
| Low-middle SDI | 5974650 (4287176,7898716) | 19651612 (14232584,25622361) | 228.92 | 6434.27 (6428.77,6439.79) | 8550.09(8546.20,8553.97) | 0.9 (0.83,0.98) |
| Low SDI | 1877587 (1339308,2499791) | 5412370 (3904277,7117717) | 188.26 | 5525.85 (5516.99,5534.74) | 7096.22(7089.84,7102.61) | 0.8 (0.75,0.86) |
| Andean Latin America | 323709 (237372,422737) | 1099260 (807301,1432037) | 239.58 | 9935.00 (9900.52,9969.58) | 11221.71(11200.72,11242.73) | 0.32 (0.22,0.41) |
| Australasia | 446329 (333742,570121) | 1065964 (778651,1377265) | 138.83 | 11163.10 (11130.06,11196.25) | 11641.05(11618.71,11663.41) | 0.13 (0.13,0.14) |
| Caribbean | 393993 (287958,509093) | 979748 (723317,1259506) | 148.67 | 9227.63 (9198.39,9257.01) | 10634.94(10613.85,10656.06) | 0.46 (0.45,0.46) |
| Central Asia | 1129782 (831742,1473131) | 2536112 (1887804,3236256) | 124.48 | 14716.95 (14689.20,14744.75) | 18391.70(18368.00,18415.43) | 0.78 (0.7,0.86) |
| Central Europe | 3235638 (2401584,4193508) | 5608738 (4178170,7221378) | 73.34 | 12511.06 (12497.00,12525.15) | 14609.18(14596.92,14621.44) | 0.55 (0.52,0.59) |
| Central Latin America | 1282322 (936078,1669000) | 5221041 (3894915,6709582) | 307.16 | 9780.53 (9763.30,9797.80) | 12406.63(12395.96,12417.31) | 0.85 (0.81,0.88) |
| Central Sub-Saharan Africa | 286458 (205758,379077) | 761623 (556346,1001041) | 165.88 | 8344.97 (8307.87,8382.41) | 9356.78(9333.17,9380.49) | 0.28 (0.16,0.41) |
| East Asia | 6474819 (4565892,8799433) | 29005400 (20943224,38343977) | 347.97 | 4827.47 (4823.05,4831.91) | 7676.79(7673.94,7679.64) | 1.5 (1.31,1.69) |
| Eastern Europe | 8676096 (6414377,11140675) | 11754929 (8723936,15081051) | 35.49 | 18083.84 (18071.44,18096.26) | 18810.95(18800.04,18821.85) | 0.34 (0.27,0.4) |
| Eastern Sub-Saharan Africa | 674291 (484655,896905) | 2155816 (1560525,2833167) | 219.72 | 6051.82 (6035.46,6068.27) | 8636.20(8623.92,8648.51) | 1.18 (1.15,1.2) |
| High-income Asia Pacific | 4123794 (3020750,5333194) | 10972964 (8202066,14050396) | 166.09 | 12102.35 (12090.50,12114.21) | 14182.66(14173.80,14191.53) | 1.12 (0.54,1.7) |
| High-income North America | 8307277 (6125861,10652798) | 17046760 (12547199,21892837) | 105.20 | 13867.55 (13858.02,13877.08) | 14776.22(14769.17,14783.28) | 0.18 (-0.15,0.5) |
| North Africa and Middle East | 1698658 (1234421,2249174) | 6307230 (4588359,8215473) | 271.31 | 6451.05 (6440.83,6461.30) | 8726.75(8719.73,8733.78) | 0.92 (0.78,1.07) |
| Oceania | 26838 (19263,35388) | 87030 (63268,114637) | 224.28 | 6246.98 (6157.53,6338.90) | 7818.75(7761.71,7876.32) | 0.76 (0.74,0.78) |
| South Asia | 5996429 (4304862,7965672) | 23201113 (16867885,30382703) | 286.92 | 6900.45 (6894.44,6906.48) | 9759.00(9754.90,9763.10) | 1.14 (1.09,1.19) |
| Southeast Asia | 2438052 (1750611,3225635) | 9681586 (7050255,12701110) | 297.10 | 6233.63 (6225.41,6241.86) | 8984.00(8978.15,8989.84) | 1.14 (1.11,1.16) |
| Southern Latin America | 877626 (643358,1139240) | 1739718 (1275011,2246061) | 98.23 | 11195.07 (11171.44,11218.77) | 11652.40(11635.06,11669.76) | 0.11 (0.1,0.12) |
| Southern Sub-Saharan Africa | 478808 (349908,619094) | 1201041 (880856,1554389) | 150.84 | 11315.65 (11282.90,11348.51) | 13079.58(13055.13,13104.09) | 0.5 (0.46,0.53) |
| Tropical Latin America | 1479497 (1074960,1940611) | 5365192 (3931188,6971147) | 262.64 | 10236.33 (10219.27,10253.44) | 12313.96(12303.51,12324.41) | 0.6 (0.57,0.62) |
| Western Europe | 10111006 (7454514,13084687) | 16458887 (12157526,21097908) | 62.78 | 10148.40 (10142.07,10154.74) | 10471.56(10466.38,10476.74) | 0.08 (0.04,0.11) |
| Western Sub-Saharan Africa | 896497 (645089,1192688) | 2426746 (1753683,3192993) | 170.69 | 6672.20 (6657.57,6686.86) | 8166.62(8155.78,8177.47) | 0.78 (0.64,0.92) |
