# Supplementary material for: Global burden of hand osteoarthritis in adults aged ≥55 years: An underestimated and fastest-growing subtype, 1990 to 2021 with projections to 2050: an analysis of GBD 2021 data
Source: Medicine (Baltimore). 2026 Aug 21;105(34):e50098. doi: 10.1097/MD.0000000000050098 (PMC13502859; doi:10.1097/MD.0000000000050098)
Supplement: Supplementary file 3 [file medi-105-e50098-s003.docx]

**Table S3. The YLDs of HOA cases and rate in adults aged ≥55 years in 1990 and 2021, and the trends from 1990 to 2021.**

| **location** | **YLDs Cases** | | **Percentage change**  **(100%)** | **ASYR** | | **EAPC (95%CI)** |
| --- | --- | --- | --- | --- | --- | --- |
|  | **1990 (95% UI)** | **2021 (95% UI)** |  | **1990(95%CI)** | **2021(95%CI)** |  |
| Global | 1872004 (856592,3869916) | 4858626 (2226493,10044723) | 159.54 | 289.48 (289.05,289.90) | 330.77(330.48,331.07) | 0.5 (0.42,0.57) |
| Female | 1277182 (585975,2638253) | 3248065 (1489909,6710569) | 154.31 | 358.91 (358.29,359.54) | 411.92(411.47,412.37) | 0.6 (0.49,0.71) |
| Male | 594822 (270580,1231611) | 1610560 (734678,3334154) | 170.76 | 203.05 (202.50,203.60) | 236.53(236.16,236.90) | 0.37 (0.27,0.47) |
| High SDI | 709594 (325144,1470496) | 1465589 (675513,3027376) | 106.54 | 374.89 (374.02,375.77) | 409.16(408.49,409.83) | 0.42 (0.22,0.62) |
| High-middle SDI | 563151 (256708,1162847) | 1207549 (552645,2493361) | 114.43 | 336.96 (336.05,337.87) | 350.15(349.53,350.78) | 0.21 (0.11,0.31) |
| Middle SDI | 350988 (160065,729451) | 1396620 (638192,2906684) | 297.91 | 217.50 (216.73,218.28) | 307.33(306.81,307.84) | 1.12 (1.07,1.18) |
| Low-middle SDI | 186956 (85498,385651) | 614231 (282494,1270852) | 228.54 | 199.44 (198.48,200.41) | 265.57(264.89,266.25) | 0.92 (0.84,0.99) |
| Low SDI | 58782 (26600,120841) | 169769 (77051,349089) | 188.81 | 171.02 (169.48,172.59) | 220.65(219.53,221.77) | 0.83 (0.77,0.88) |
| Andean Latin America | 10294 (4650,21549) | 34828 (15802,71465) | 238.33 | 314.93 (308.83,321.14) | 355.11(351.39,358.87) | 0.31 (0.22,0.4) |
| Australasia | 14060 (6469,29152) | 33468 (15388,67941) | 138.04 | 351.64 (345.79,357.58) | 367.11(363.14,371.11) | 0.13 (0.12,0.14) |
| Caribbean | 12550 (5684,26003) | 30919 (14100,64159) | 146.37 | 293.36 (288.18,298.67) | 335.64(331.90,339.41) | 0.44 (0.43,0.44) |
| Central Asia | 35937 (16115,74366) | 80679 (35565,165868) | 124.50 | 466.48 (461.56,471.45) | 581.44(577.25,585.66) | 0.78 (0.7,0.86) |
| Central Europe | 101688 (46172,211549) | 175811 (80538,363269) | 72.89 | 391.62 (389.15,394.12) | 459.28(457.10,461.46) | 0.57 (0.54,0.61) |
| Central Latin America | 40442 (18292,83953) | 164548 (74738,340977) | 306.87 | 306.96 (303.92,310.03) | 390.24(388.35,392.14) | 0.85 (0.82,0.89) |
| Central Sub-Saharan Africa | 9027 (4124,18899) | 24047 (10827,49749) | 166.39 | 259.57 (253.15,266.33) | 292.14(288.02,296.37) | 0.31 (0.18,0.43) |
| East Asia | 206868 (92861,429053) | 921030 (422029,1905972) | 345.23 | 152.58 (151.80,153.37) | 242.59(242.08,243.09) | 1.49 (1.3,1.68) |
| Eastern Europe | 272761 (124918,564386) | 368108 (169543,766751) | 34.96 | 566.92 (564.73,569.12) | 589.03(587.11,590.97) | 0.34 (0.27,0.42) |
| Eastern Sub-Saharan Africa | 21257 (9652,44090) | 68082 (31090,140710) | 220.28 | 188.63 (185.78,191.57) | 270.23(268.08,272.41) | 1.2 (1.18,1.23) |
| High-income Asia Pacific | 131551 (60158,272384) | 346845 (160723,715083) | 163.66 | 384.76 (382.66,386.88) | 453.00(451.41,454.59) | 1.13 (0.55,1.71) |
| High-income North America | 260842 (119674,539621) | 528050 (243737,1087969) | 102.44 | 436.53 (434.84,438.22) | 458.91(457.66,460.15) | 0.14 (-0.19,0.47) |
| North Africa and Middle East | 53823 (24528,111145) | 198261 (89762,410239) | 268.36 | 202.70 (200.90,204.52) | 272.50(271.27,273.74) | 0.9 (0.75,1.05) |
| Oceania | 852 (386,1761) | 2751 (1247,5722) | 222.89 | 195.65 (180.30,213.77) | 244.25(234.33,254.69) | 0.75 (0.74,0.77) |
| South Asia | 186136 (85690,384526) | 720405 (330771,1494060) | 287.03 | 211.78 (210.74,212.83) | 300.98(300.27,301.70) | 1.16 (1.11,1.22) |
| Southeast Asia | 77349 (35369,159222) | 307328 (138988,639688) | 297.33 | 195.92 (194.47,197.37) | 282.93(281.90,283.97) | 1.15 (1.13,1.18) |
| Southern Latin America | 27889 (12593,56742) | 54828 (25089,111826) | 96.59 | 355.00 (350.80,359.24) | 367.72(364.65,370.82) | 0.1 (0.08,0.11) |
| Southern Sub-Saharan Africa | 15105 (6909,31498) | 37488 (16987,76830) | 148.18 | 354.87 (349.11,360.74) | 405.49(401.22,409.84) | 0.47 (0.43,0.5) |
| Tropical Latin America | 46309 (21077,96696) | 167273 (76673,344414) | 261.21 | 318.42 (315.43,321.45) | 383.21(381.37,385.06) | 0.6 (0.57,0.63) |
| Western Europe | 319061 (146076,656528) | 517097 (237647,1061388) | 62.07 | 320.83 (319.70,321.96) | 331.46(330.54,332.39) | 0.08 (0.04,0.12) |
| Western Sub-Saharan Africa | 28202 (12819,58277) | 76779 (34950,160567) | 172.25 | 208.19 (205.63,210.79) | 256.26(254.35,258.18) | 0.81 (0.67,0.95) |
