# Supplementary material for: Global burden of hand osteoarthritis in adults aged ≥55 years: An underestimated and fastest-growing subtype, 1990 to 2021 with projections to 2050: an analysis of GBD 2021 data
Source: Medicine (Baltimore). 2026 Aug 21;105(34):e50098. doi: 10.1097/MD.0000000000050098 (PMC13502859; doi:10.1097/MD.0000000000050098)
Supplement: Supplementary file 4 [file medi-105-e50098-s004.docx]

**Table S4. The incidence of HOA cases and rate in adults aged ≥55 years across 204 countries in 1990 and 2021, and the trends from 1990 to 2021.**

| **location** | **Incidence Cases** | | **Percentage change**  **(100%)** | **ASIR** | | **EAPC (95%CI)** |
| --- | --- | --- | --- | --- | --- | --- |
|  | **1990 (95% UI)** | **2021 (95% UI)** |  | **1990(95%CI)** | **2021(95%CI)** |  |
| Afghanistan | 1892 (1274,2797) | 2318 (1560,3386) | 22.52 | 156.13 (148.51,164.82) | 188.14(180.09,196.85) | 0.59 (0.51,0.67) |
| Albania | 806 (544,1158) | 2251 (1543,3171) | 179.28 | 229.26 (213.39,246.26) | 288.69(276.81,301.08) | 0.9 (0.84,0.96) |
| Algeria | 4723 (3168,6969) | 17253 (11346,25573) | 265.30 | 222.30 (214.61,232.90) | 274.61(270.31,279.18) | 0.67 (0.62,0.73) |
| American Samoa | 13 (9,20) | 33 (21,50) | 153.85 | 337.83 (171.09,1092.41) | 364.67(247.31,581.29) | 0.22 (0.2,0.23) |
| Andorra | 28 (19,40) | 78 (53,112) | 178.57 | 280.34 (181.74,767.60) | 290.96(229.71,364.12) | 0.12 (0.11,0.14) |
| Angola | 2313 (1520,3622) | 8129 (5438,12400) | 251.45 | 340.00 (324.60,357.55) | 380.27(371.23,389.89) | 0.35 (0.33,0.36) |
| Antigua and Barbuda | 27 (19,40) | 68 (46,99) | 151.85 | 335.00 (220.16,491.53) | 350.29(271.38,457.32) | 0.11 (0.09,0.12) |
| Argentina | 19197 (12826,28130) | 32220 (21556,46555) | 67.84 | 344.62 (339.73,349.58) | 355.65(351.77,359.57) | 0.05 (0.03,0.07) |
| Armenia | 1846 (1207,2820) | 3472 (2364,5056) | 88.08 | 347.22 (330.86,364.45) | 441.18(426.44,456.44) | 0.92 (0.87,0.98) |
| Australia | 10544 (7165,15237) | 24239 (16690,35389) | 129.88 | 332.27 (325.90,338.75) | 345.43(341.05,349.86) | 0.12 (0.11,0.13) |
| Austria | 5233 (3568,7459) | 8243 (5658,11995) | 57.52 | 284.41 (276.60,292.45) | 291.25(284.93,297.68) | 0.07 (0.06,0.08) |
| Azerbaijan | 3952 (2659,5841) | 9977 (6593,14921) | 152.45 | 406.07 (392.99,419.88) | 446.76(437.52,456.25) | 0.39 (0.29,0.49) |
| Bahamas | 84 (56,126) | 268 (178,406) | 219.05 | 337.66 (268.98,422.55) | 352.05(310.49,398.73) | 0.14 (0.13,0.15) |
| Bahrain | 83 (53,131) | 497 (312,767) | 498.80 | 275.06 (203.70,645.84) | 277.86(246.33,371.93) | -0.04 (-0.08,-0.01) |
| Bangladesh | 16745 (11324,24919) | 64123 (43165,92249) | 282.94 | 217.60 (214.25,221.03) | 271.52(269.39,273.68) | 0.74 (0.65,0.84) |
| Barbados | 144 (98,205) | 317 (211,475) | 120.14 | 332.15 (278.53,397.57) | 351.98(314.20,393.46) | 0.19 (0.17,0.21) |
| Belarus | 8300 (5463,12429) | 11021 (7390,15942) | 32.78 | 350.25 (342.66,358.00) | 384.22(377.02,391.52) | 0.37 (0.35,0.4) |
| Belgium | 7368 (5017,10642) | 10631 (7400,15619) | 44.29 | 290.45 (283.80,297.27) | 297.14(291.44,302.93) | 0.07 (0.06,0.08) |
| Belize | 41 (28,61) | 166 (111,238) | 304.88 | 267.55 (192.24,363.34) | 315.28(268.54,368.80) | 0.47 (0.44,0.51) |
| Benin | 675 (461,954) | 2375 (1562,3453) | 251.85 | 211.57 (195.72,228.85) | 278.45(267.05,290.44) | 0.9 (0.89,0.9) |
| Bermuda | 36 (24,54) | 80 (54,118) | 122.22 | 341.08 (238.72,491.06) | 354.00(280.33,441.70) | 0.11 (0.09,0.12) |
| Bhutan | 81 (54,121) | 246 (164,361) | 203.70 | 202.98 (157.68,274.14) | 246.05(216.17,279.95) | 0.6 (0.57,0.63) |
| Bolivia (Plurinational State of) | 1465 (981,2100) | 4941 (3282,7153) | 237.27 | 272.55 (258.34,288.09) | 312.15(303.36,321.29) | 0.43 (0.36,0.51) |
| Bosnia and Herzegovina | 2261 (1511,3274) | 3863 (2574,5722) | 70.85 | 291.45 (278.82,305.05) | 359.82(348.50,371.47) | 0.79 (0.67,0.9) |
| Botswana | 268 (177,391) | 908 (601,1363) | 238.81 | 280.99 (244.71,337.75) | 354.47(330.75,380.94) | 0.68 (0.64,0.72) |
| Brazil | 53628 (36479,77084) | 179648 (123421,259267) | 234.99 | 357.61 (354.51,360.75) | 410.62(408.72,412.53) | 0.44 (0.41,0.46) |
| Brunei Darussalam | 62 (41,92) | 260 (170,399) | 319.35 | 374.08 (283.18,619.80) | 394.17(342.13,580.79) | 0.13 (0.12,0.15) |
| Bulgaria | 8023 (5394,11939) | 8609 (5881,12602) | 7.30 | 349.89 (341.07,360.14) | 392.22(383.77,400.93) | 0.39 (0.35,0.43) |
| Burkina Faso | 1190 (806,1754) | 2748 (1902,4076) | 130.92 | 167.31 (157.03,179.16) | 183.64(176.53,191.15) | 0.31 (0.28,0.34) |
| Burundi | 775 (519,1118) | 1737 (1150,2559) | 124.13 | 206.93 (191.45,226.27) | 212.52(201.83,224.25) | 0.14 (0.1,0.17) |
| Cabo Verde | 83 (57,120) | 232 (157,336) | 179.52 | 210.10 (167.00,261.86) | 291.06(254.09,332.71) | 1.18 (1.13,1.22) |
| Cambodia | 1250 (843,1816) | 5337 (3569,7713) | 326.96 | 170.64 (160.43,182.24) | 241.16(234.27,248.45) | 1.23 (1.19,1.27) |
| Cameroon | 1499 (1009,2204) | 5400 (3561,7805) | 260.24 | 200.17 (189.54,211.86) | 256.45(249.25,264.03) | 0.78 (0.72,0.85) |
| Canada | 19232 (13109,28142) | 43665 (29736,63768) | 127.04 | 364.85 (359.68,370.07) | 372.87(369.36,376.41) | 0.07 (0.06,0.08) |
| Central African Republic | 469 (308,703) | 938 (607,1425) | 100.00 | 240.20 (214.59,273.65) | 250.87(230.98,279.75) | 0.05 (0.02,0.09) |
| Chad | 842 (587,1223) | 1813 (1225,2662) | 115.32 | 184.66 (172.00,198.68) | 193.68(184.34,203.81) | 0.13 (0.09,0.18) |
| Chile | 5234 (3504,7639) | 15100 (10143,22050) | 188.50 | 306.93 (298.60,315.57) | 336.37(331.03,341.78) | 0.27 (0.25,0.28) |
| China | 289275 (191121,423860) | 1148737 (778955,1643125) | 297.11 | 208.70 (207.81,209.61) | 305.02(304.45,305.59) | 1.05 (0.85,1.25) |
| Colombia | 8194 (5371,11641) | 31164 (21123,46879) | 280.33 | 275.59 (269.58,281.73) | 320.74(317.18,324.33) | 0.58 (0.55,0.61) |
| Comoros | 67 (44,98) | 218 (146,313) | 225.37 | 204.86 (154.15,310.05) | 263.19(228.45,305.23) | 0.92 (0.88,0.96) |
| Congo | 575 (377,845) | 1594 (1039,2386) | 177.22 | 309.46 (279.07,367.54) | 339.59(321.55,361.30) | 0.28 (0.24,0.32) |
| Cook Islands | 7 (5,10) | 17 (12,26) | 142.86 | 320.07 (125.01,1267.62) | 364.74(213.77,613.83) | 0.41 (0.38,0.43) |
| Costa Rica | 779 (528,1121) | 3090 (2062,4651) | 296.66 | 271.67 (252.91,291.59) | 317.26(306.15,328.69) | 0.5 (0.49,0.52) |
| Croatia | 4200 (2821,6133) | 5342 (3718,7465) | 27.19 | 360.59 (349.45,372.35) | 380.60(370.31,391.16) | 0.24 (0.22,0.26) |
| Cuba | 4714 (3198,6832) | 10842 (7451,15570) | 130.00 | 282.74 (274.54,291.70) | 314.39(308.46,320.41) | 0.35 (0.34,0.37) |
| Cyprus | 362 (244,538) | 985 (674,1429) | 172.10 | 268.69 (225.04,1067.35) | 294.55(275.49,322.84) | 0.27 (0.23,0.3) |
| Czechia | 9001 (6171,13372) | 12681 (8744,18578) | 40.88 | 387.98 (379.88,396.32) | 395.88(388.86,402.99) | 0.06 (0.04,0.08) |
| C么te d'Ivoire | 1364 (894,2039) | 4772 (3123,7041) | 249.85 | 208.04 (195.71,221.98) | 255.38(247.69,263.47) | 0.62 (0.57,0.67) |
| Democratic People's Republic of Korea | 5140 (3388,7579) | 13880 (9245,20548) | 170.04 | 193.66 (188.10,199.45) | 245.08(240.93,249.33) | 0.75 (0.72,0.77) |
| Democratic Republic of the Congo | 7248 (4829,11115) | 16976 (11444,24928) | 134.22 | 260.84 (253.84,268.31) | 268.68(264.26,273.24) | 0 (-0.14,0.14) |
| Denmark | 4018 (2799,5862) | 5199 (3594,7591) | 29.39 | 340.20 (329.59,351.09) | 290.66(282.69,298.82) | -0.37 (-0.45,-0.3) |
| Djibouti | 49 (32,73) | 323 (212,475) | 559.18 | 221.51 (155.19,369.31) | 292.10(257.11,342.15) | 1.08 (0.99,1.17) |
| Dominica | 27 (18,39) | 48 (32,69) | 77.78 | 287.43 (188.64,439.69) | 311.76(229.44,418.91) | 0.23 (0.2,0.25) |
| Dominican Republic | 1893 (1247,2715) | 5586 (3843,7968) | 195.09 | 301.27 (287.60,315.78) | 329.46(320.86,338.24) | 0.27 (0.26,0.29) |
| Ecuador | 2628 (1756,3839) | 9125 (6168,13377) | 247.22 | 299.26 (287.83,311.37) | 327.41(320.71,334.27) | 0.38 (0.35,0.42) |
| Egypt | 9448 (6204,14059) | 28419 (18669,41840) | 200.79 | 204.41 (199.84,209.35) | 241.03(237.95,244.18) | 0.39 (0.34,0.44) |
| El Salvador | 1336 (894,1946) | 3209 (2170,4689) | 140.19 | 269.17 (254.90,284.07) | 316.78(305.87,327.98) | 0.57 (0.56,0.58) |
| Equatorial Guinea | 67 (44,100) | 294 (195,443) | 338.81 | 209.26 (156.16,330.27) | 348.40(307.72,400.09) | 1.97 (1.84,2.11) |
| Eritrea | 351 (232,523) | 1129 (741,1680) | 221.65 | 197.77 (170.36,244.76) | 248.37(231.54,271.33) | 0.77 (0.75,0.79) |
| Estonia | 1321 (876,1959) | 1579 (1067,2282) | 19.53 | 366.65 (346.95,387.92) | 391.45(371.96,411.74) | 0.24 (0.19,0.28) |
| Eswatini | 113 (75,168) | 311 (209,471) | 175.22 | 247.46 (201.33,316.19) | 331.21(290.16,417.59) | 0.94 (0.91,0.97) |
| Ethiopia | 8293 (5515,12066) | 28860 (19797,40782) | 248.00 | 261.80 (254.42,270.15) | 411.89(407.04,416.82) | 1.73 (1.65,1.8) |
| Fiji | 169 (110,247) | 511 (347,766) | 202.37 | 285.91 (240.97,347.78) | 351.38(318.61,400.62) | 0.7 (0.66,0.74) |
| Finland | 3168 (2157,4740) | 5338 (3733,7663) | 68.50 | 277.87 (268.19,287.88) | 291.72(283.71,299.92) | 0.15 (0.13,0.17) |
| France | 38715 (26504,55381) | 60532 (41458,86957) | 56.35 | 288.65 (285.74,291.58) | 295.51(293.10,297.93) | 0.09 (0.08,0.1) |
| Gabon | 265 (179,395) | 610 (396,888) | 130.19 | 267.03 (234.80,307.13) | 321.94(295.42,353.05) | 0.53 (0.49,0.57) |
| Gambia | 120 (81,173) | 463 (310,676) | 285.83 | 216.17 (176.20,286.18) | 292.11(265.43,323.47) | 1.05 (1.01,1.08) |
| Georgia | 4290 (2880,6459) | 4218 (2997,6012) | -1.68 | 372.12 (360.92,383.73) | 412.27(399.83,425.03) | 0.28 (0.18,0.37) |
| Germany | 58723 (40573,86322) | 88444 (60228,126541) | 50.61 | 290.59 (288.22,292.98) | 296.20(294.22,298.19) | -0.11 (-0.18,-0.05) |
| Ghana | 3557 (2322,5548) | 9004 (5971,13459) | 153.13 | 328.84 (317.48,341.07) | 316.23(309.41,323.33) | -0.01 (-0.32,0.29) |
| Greece | 6434 (4413,9287) | 9355 (6453,13214) | 45.40 | 242.52 (236.58,248.68) | 276.47(270.72,282.32) | 1.62 (1.17,2.08) |
| Greenland | 21 (14,31) | 55 (36,81) | 161.90 | 343.12 (199.82,1374.73) | 363.40(268.85,527.17) | 0.17 (0.16,0.18) |
| Grenada | 34 (23,50) | 68 (45,98) | 100.00 | 294.17 (203.42,413.20) | 325.98(250.93,427.05) | 0.29 (0.25,0.34) |
| Guam | 46 (30,68) | 142 (97,208) | 208.70 | 330.94 (231.67,877.88) | 369.96(311.17,437.67) | 0.34 (0.32,0.37) |
| Guatemala | 1448 (952,2175) | 5144 (3500,7403) | 255.25 | 242.91 (229.47,259.92) | 278.16(270.47,286.38) | 0.45 (0.44,0.46) |
| Guinea | 974 (667,1404) | 1919 (1288,2741) | 97.02 | 177.70 (166.43,189.96) | 208.48(199.04,218.42) | 0.48 (0.44,0.51) |
| Guinea-Bissau | 125 (85,187) | 278 (186,409) | 122.40 | 196.69 (159.41,266.27) | 235.87(205.10,284.67) | 0.58 (0.54,0.62) |
| Guyana | 175 (117,253) | 385 (259,575) | 120.00 | 279.93 (239.38,330.49) | 324.84(292.38,361.61) | 0.48 (0.46,0.49) |
| Haiti | 1129 (764,1630) | 3116 (2075,4513) | 176.00 | 207.24 (194.07,222.73) | 251.96(242.64,262.00) | 0.67 (0.66,0.68) |
| Honduras | 851 (562,1250) | 3030 (2017,4417) | 256.05 | 250.15 (233.00,270.92) | 281.90(271.69,292.81) | 0.42 (0.38,0.45) |
| Hungary | 9653 (6531,14289) | 11766 (8109,17349) | 21.89 | 378.61 (370.95,386.54) | 403.52(396.06,411.10) | 0.16 (0.11,0.21) |
| Iceland | 148 (102,213) | 262 (175,385) | 77.03 | 323.74 (273.49,380.77) | 278.13(245.26,314.31) | -0.71 (-1.08,-0.33) |
| India | 206329 (138667,301557) | 711192 (490682,1026672) | 244.69 | 266.67 (265.41,267.94) | 350.21(349.38,351.05) | 0.91 (0.86,0.95) |
| Indonesia | 44208 (29671,65468) | 156711 (105745,225663) | 254.49 | 273.31 (270.59,276.07) | 364.30(362.36,366.27) | 0.86 (0.81,0.91) |
| Iran (Islamic Republic of) | 12799 (8504,18915) | 44621 (30291,66248) | 248.63 | 275.48 (270.10,280.99) | 336.24(333.08,339.42) | 0.72 (0.68,0.76) |
| Iraq | 2956 (1998,4341) | 10442 (6983,15693) | 253.25 | 231.06 (222.77,239.59) | 258.68(253.54,263.95) | 0.22 (0.17,0.27) |
| Ireland | 1758 (1199,2535) | 3680 (2529,5333) | 109.33 | 272.60 (259.80,286.18) | 291.72(282.33,301.36) | 0.18 (0.16,0.21) |
| Israel | 2233 (1522,3165) | 5630 (3907,8374) | 152.13 | 288.05 (276.02,300.90) | 299.30(291.44,307.34) | -1.25 (-1.77,-0.72) |
| Italy | 51222 (35068,73552) | 78409 (54027,111515) | 53.08 | 344.20 (341.19,347.24) | 364.19(361.58,366.81) | 0.53 (0.28,0.79) |
| Jamaica | 824 (559,1186) | 1678 (1152,2389) | 103.64 | 288.09 (268.63,308.63) | 313.24(298.34,328.71) | 0.27 (0.25,0.28) |
| Japan | 132905 (93059,190904) | 216973 (156839,300114) | 63.25 | 446.18 (443.77,448.60) | 470.46(468.32,472.61) | 0.47 (0.17,0.77) |
| Jordan | 574 (370,850) | 3802 (2510,5767) | 562.37 | 252.84 (231.53,276.60) | 282.31(272.92,292.13) | 0.37 (0.33,0.41) |
| Kazakhstan | 8903 (5977,13794) | 17063 (11406,25531) | 91.65 | 407.25 (398.69,415.99) | 491.52(483.98,499.30) | 0.66 (0.63,0.69) |
| Kenya | 4138 (2804,5998) | 15734 (10577,23234) | 280.23 | 307.73 (298.05,317.99) | 408.49(401.76,415.40) | 0.94 (0.91,0.98) |
| Kiribati | 20 (13,28) | 47 (31,70) | 135.00 | 316.34 (183.21,756.41) | 343.90(241.90,658.46) | 0.18 (0.15,0.22) |
| Kuwait | 250 (160,383) | 1437 (909,2174) | 474.80 | 254.61 (222.18,291.97) | 277.86(262.69,293.98) | 0.33 (0.3,0.36) |
| Kyrgyzstan | 2138 (1431,3230) | 4295 (2873,6375) | 100.89 | 393.31 (376.51,411.16) | 449.01(435.16,463.44) | 0.58 (0.47,0.68) |
| Lao People's Democratic Republic | 625 (411,928) | 1956 (1335,2938) | 212.96 | 184.50 (168.07,206.87) | 246.30(234.99,258.37) | 0.97 (0.9,1.03) |
| Latvia | 2233 (1477,3254) | 2378 (1623,3521) | 6.49 | 355.66 (340.96,371.08) | 386.21(370.60,402.33) | 0.33 (0.3,0.37) |
| Lebanon | 929 (608,1338) | 2860 (1939,4244) | 207.86 | 238.17 (222.63,254.97) | 296.82(285.99,307.97) | 0.71 (0.68,0.75) |
| Lesotho | 339 (228,488) | 612 (406,884) | 80.53 | 242.84 (217.03,273.36) | 331.25(303.15,369.02) | 1.09 (1.03,1.16) |
| Liberia | 384 (260,568) | 891 (590,1339) | 132.03 | 206.23 (185.11,232.03) | 258.21(240.78,277.30) | 0.84 (0.8,0.89) |
| Libya | 788 (515,1182) | 2487 (1623,3674) | 215.61 | 245.07 (228.01,263.20) | 280.13(268.93,291.75) | 0.44 (0.38,0.5) |
| Lithuania | 2799 (1838,4231) | 3556 (2428,5339) | 27.05 | 348.39 (335.50,361.80) | 386.11(373.39,399.17) | 0.42 (0.38,0.45) |
| Luxembourg | 263 (178,380) | 521 (363,756) | 98.10 | 287.27 (253.27,327.75) | 296.30(271.34,322.97) | 0.06 (0.04,0.08) |
| Madagascar | 1491 (1016,2177) | 3690 (2454,5522) | 147.48 | 176.54 (167.19,186.66) | 196.24(189.04,203.99) | 0.32 (0.31,0.32) |
| Malawi | 1226 (823,1798) | 2778 (1820,3982) | 126.59 | 195.35 (183.47,208.91) | 236.31(227.05,246.17) | 0.69 (0.65,0.73) |
| Malaysia | 4234 (2845,6212) | 17215 (11676,25273) | 306.59 | 279.49 (271.05,288.17) | 340.92(335.77,346.17) | 0.59 (0.53,0.64) |
| Maldives | 48 (31,71) | 206 (136,315) | 329.17 | 285.31 (199.94,668.53) | 352.34(304.44,408.00) | 0.58 (0.51,0.65) |
| Mali | 1251 (844,1836) | 3365 (2278,4868) | 168.98 | 186.95 (175.66,199.80) | 227.53(219.24,236.82) | 0.67 (0.64,0.69) |
| Malta | 209 (143,298) | 427 (294,625) | 104.31 | 292.69 (254.13,338.35) | 298.36(269.83,329.31) | 0.04 (0.02,0.05) |
| Marshall Islands | 9 (6,12) | 24 (16,37) | 166.67 | 328.12 (133.93,2409.10) | 370.02(217.55,1253.91) | 0.37 (0.35,0.4) |
| Mauritania | 349 (240,508) | 977 (660,1449) | 179.94 | 211.71 (188.90,241.99) | 266.20(249.47,284.23) | 0.73 (0.65,0.81) |
| Mauritius | 379 (251,552) | 1252 (848,1891) | 230.34 | 305.54 (274.62,341.80) | 360.67(340.80,381.56) | 0.55 (0.53,0.57) |
| Mexico | 24598 (16566,35755) | 92904 (63098,135118) | 277.69 | 346.64 (342.22,351.17) | 421.85(419.13,424.58) | 0.74 (0.69,0.8) |
| Micronesia (Federated States of) | 23 (16,34) | 50 (33,78) | 117.39 | 291.03 (180.93,623.07) | 351.06(252.48,552.41) | 0.65 (0.61,0.69) |
| Monaco | 29 (20,41) | 43 (30,62) | 48.28 | 290.23 (190.80,427.76) | 295.57(212.14,402.20) | 0.04 (0.03,0.06) |
| Mongolia | 645 (440,975) | 2291 (1519,3388) | 255.19 | 357.06 (329.50,388.13) | 486.95(465.84,512.40) | 1.17 (1.1,1.24) |
| Montenegro | 413 (279,630) | 667 (449,946) | 61.50 | 364.30 (329.61,402.30) | 386.81(357.61,420.14) | 0.24 (0.21,0.26) |
| Morocco | 5830 (3874,8479) | 15644 (10442,23320) | 168.34 | 245.43 (239.09,251.96) | 253.08(249.03,257.21) | -0.05 (-0.14,0.03) |
| Mozambique | 1829 (1242,2683) | 4157 (2747,6117) | 127.28 | 187.63 (178.30,198.15) | 229.64(222.10,237.62) | 0.66 (0.62,0.69) |
| Myanmar | 7929 (5277,11548) | 25208 (17065,36403) | 217.92 | 201.75 (196.96,206.79) | 289.99(286.33,293.71) | 1.32 (1.26,1.38) |
| Namibia | 265 (176,392) | 725 (483,1044) | 173.58 | 242.24 (211.20,282.19) | 310.96(287.65,337.84) | 0.73 (0.67,0.79) |
| Nauru | 2 (2,3) | 4 (2,5) | 100.00 | 303.69 (26.20,16219.17) | 359.11(73.73,4292.93) | 0.58 (0.54,0.62) |
| Nepal | 2702 (1839,3939) | 8801 (5948,12945) | 225.72 | 175.33 (168.28,182.92) | 221.71(216.96,226.58) | 0.72 (0.67,0.76) |
| Netherlands | 8846 (6525,12184) | 16258 (11157,23389) | 83.79 | 281.28 (275.42,287.26) | 293.91(289.37,298.51) | -0.14 (-0.47,0.19) |
| New Zealand | 2533 (1776,3645) | 5847 (4108,8334) | 130.83 | 394.46 (379.11,410.40) | 420.02(409.27,430.99) | 0.17 (0.15,0.19) |
| Nicaragua | 595 (393,864) | 2394 (1577,3481) | 302.35 | 234.81 (216.12,254.98) | 287.01(275.53,299.10) | 0.66 (0.65,0.67) |
| Niger | 776 (524,1167) | 2726 (1848,4021) | 251.29 | 178.47 (164.54,195.65) | 193.92(186.01,202.45) | 0.3 (0.27,0.34) |
| Nigeria | 20030 (13613,28985) | 52828 (35739,77866) | 163.74 | 277.47 (273.50,281.52) | 341.49(338.46,344.56) | 0.87 (0.75,1) |
| Niue | 1 (1,2) | 1 (1,2) | 0.00 | 323.42 (11.91,1910.32) | 366.14(24.50,2686.55) | 0.42 (0.4,0.44) |
| North Macedonia | 1099 (730,1617) | 2130 (1406,3192) | 93.81 | 319.18 (300.27,339.15) | 358.69(340.64,405.23) | 0.42 (0.39,0.46) |
| Northern Mariana Islands | 9 (6,14) | 39 (26,59) | 333.33 | 336.97 (132.77,3214.94) | 361.63(246.85,609.53) | 0.17 (0.15,0.18) |
| Norway | 3632 (2507,5137) | 5799 (4011,8352) | 59.66 | 361.05 (348.99,373.46) | 374.84(365.12,384.76) | 0.29 (0.06,0.53) |
| Oman | 261 (168,396) | 940 (605,1390) | 260.15 | 253.96 (223.14,290.54) | 279.97(260.78,301.68) | 0.32 (0.29,0.35) |
| Pakistan | 25564 (17405,36311) | 71036 (47814,107051) | 177.88 | 280.41 (276.90,283.98) | 347.25(344.59,349.93) | 0.65 (0.62,0.68) |
| Palau | 5 (4,8) | 17 (11,26) | 240.00 | 321.08 (99.72,1975.41) | 362.05(196.89,1362.52) | 0.38 (0.36,0.4) |
| Palestine | 332 (223,482) | 1260 (833,1923) | 279.52 | 226.23 (202.12,253.89) | 276.60(260.68,294.27) | 0.61 (0.57,0.65) |
| Panama | 661 (447,977) | 2322 (1564,3303) | 251.29 | 270.81 (250.51,292.50) | 311.11(298.57,324.04) | 0.44 (0.43,0.45) |
| Papua New Guinea | 590 (388,854) | 2085 (1425,3117) | 253.39 | 196.16 (177.24,220.94) | 242.58(231.18,254.99) | 0.68 (0.66,0.7) |
| Paraguay | 1153 (777,1692) | 3175 (2149,4570) | 175.37 | 313.22 (295.35,332.00) | 312.81(301.97,323.98) | -0.04 (-0.19,0.1) |
| Peru | 6426 (4304,9311) | 19239 (12902,27851) | 199.39 | 315.21 (307.50,323.07) | 339.41(334.62,344.25) | 0.13 (0.02,0.23) |
| Philippines | 15679 (10591,22698) | 56031 (37979,81367) | 257.36 | 323.35 (317.46,329.99) | 391.16(387.83,394.52) | 0.5 (0.44,0.55) |
| Poland | 33193 (22681,47160) | 56557 (39294,80465) | 70.39 | 427.82 (423.17,432.53) | 485.52(481.45,489.61) | 0.43 (0.39,0.47) |
| Portugal | 6390 (4393,9301) | 10358 (7190,14755) | 62.10 | 267.84 (261.22,274.73) | 287.75(282.11,293.49) | 0.53 (0.34,0.72) |
| Puerto Rico | 1984 (1332,2834) | 3837 (2611,5504) | 93.40 | 335.06 (320.36,350.59) | 352.86(341.51,364.52) | 0.16 (0.15,0.17) |
| Qatar | 45 (28,71) | 460 (281,715) | 922.22 | 246.04 (164.84,577.26) | 261.69(230.82,306.29) | 0.1 (0.06,0.14) |
| Republic of Korea | 18085 (11987,26670) | 63939 (43185,96259) | 253.55 | 341.53 (336.22,347.18) | 379.50(376.55,382.46) | 0.32 (0.15,0.5) |
| Republic of Moldova | 2373 (1559,3399) | 3601 (2441,5169) | 51.75 | 300.42 (287.98,313.95) | 341.98(330.84,353.41) | 0.59 (0.5,0.68) |
| Romania | 16364 (10985,23589) | 19348 (12990,28231) | 18.24 | 319.74 (314.61,325.13) | 345.86(340.83,350.96) | 0.32 (0.29,0.35) |
| Russian Federation | 150622 (101816,222102) | 212511 (145399,309742) | 41.09 | 469.81 (467.38,472.27) | 505.49(503.31,507.67) | 0.25 (0.22,0.27) |
| Rwanda | 820 (547,1224) | 2234 (1511,3318) | 172.44 | 177.15 (163.59,193.57) | 209.90(200.61,219.99) | 0.57 (0.55,0.59) |
| Saint Kitts and Nevis | 19 (13,27) | 47 (31,70) | 147.37 | 322.39 (188.80,589.74) | 341.72(247.11,476.34) | 0.18 (0.16,0.19) |
| Saint Lucia | 40 (27,57) | 139 (94,200) | 247.50 | 278.77 (197.42,410.69) | 322.75(271.13,383.23) | 0.4 (0.34,0.45) |
| Saint Vincent and the Grenadines | 34 (23,48) | 81 (55,116) | 138.24 | 287.37 (197.41,437.23) | 312.03(247.11,401.55) | 0.25 (0.23,0.27) |
| Samoa | 42 (28,61) | 88 (60,128) | 109.52 | 281.64 (200.27,414.19) | 343.84(274.60,437.38) | 0.78 (0.72,0.84) |
| San Marino | 16 (11,24) | 33 (23,49) | 106.25 | 286.89 (163.60,469.77) | 294.91(201.45,418.00) | 0.09 (0.08,0.09) |
| Sao Tome and Principe | 28 (19,40) | 65 (43,100) | 132.14 | 246.96 (162.73,375.21) | 336.59(257.53,458.99) | 1.08 (1.02,1.14) |
| Saudi Arabia | 2135 (1438,3228) | 9233 (5757,13889) | 332.46 | 233.50 (223.43,244.11) | 273.18(266.81,279.83) | 0.28 (0.18,0.39) |
| Senegal | 1131 (761,1611) | 3457 (2332,5136) | 205.66 | 213.51 (200.75,227.87) | 261.04(252.17,270.31) | 0.63 (0.6,0.65) |
| Serbia | 6844 (4522,9811) | 9589 (6610,14143) | 40.11 | 315.26 (307.33,323.73) | 358.68(351.44,366.04) | 0.48 (0.46,0.5) |
| Seychelles | 27 (18,40) | 79 (53,115) | 192.59 | 285.15 (188.09,416.92) | 362.38(285.64,459.22) | 0.74 (0.66,0.83) |
| Sierra Leone | 619 (429,907) | 1513 (1011,2217) | 144.43 | 189.05 (174.05,205.71) | 245.85(233.22,259.48) | 0.89 (0.82,0.97) |
| Singapore | 1502 (1003,2193) | 6097 (4108,9145) | 305.93 | 393.70 (373.75,414.95) | 393.59(383.74,403.65) | -0.04 (-0.04,-0.03) |
| Slovakia | 3912 (2649,5851) | 6337 (4303,9231) | 61.99 | 378.25 (366.35,390.56) | 400.67(390.79,410.75) | 0.18 (0.15,0.22) |
| Slovenia | 1670 (1118,2414) | 2688 (1849,3895) | 60.96 | 381.81 (363.52,401.11) | 388.55(373.81,403.75) | 0.03 (0,0.06) |
| Solomon Islands | 57 (38,86) | 176 (117,256) | 208.77 | 243.13 (173.75,539.34) | 307.91(261.01,389.13) | 0.79 (0.75,0.83) |
| Somalia | 731 (480,1097) | 2064 (1348,3018) | 182.35 | 198.44 (182.24,218.17) | 217.17(205.04,234.03) | 0.35 (0.32,0.38) |
| South Africa | 14821 (10053,21446) | 37920 (25922,54228) | 155.85 | 431.33 (424.35,438.44) | 467.91(463.11,472.80) | 0.27 (0.24,0.29) |
| South Sudan | 688 (472,1001) | 1216 (835,1800) | 76.74 | 170.03 (156.78,185.25) | 191.08(179.66,203.42) | 0.44 (0.41,0.48) |
| Spain | 25185 (17290,36553) | 42723 (29629,61572) | 69.64 | 268.84 (265.51,272.20) | 291.53(288.73,294.36) | 0.11 (0.05,0.17) |
| Sri Lanka | 4284 (2884,6220) | 15017 (10265,21579) | 250.54 | 236.76 (229.35,244.94) | 310.11(305.12,315.21) | 0.86 (0.83,0.89) |
| Sudan | 2421 (1632,3561) | 7129 (4754,10433) | 194.47 | 165.38 (158.47,172.86) | 222.19(216.93,227.59) | 0.99 (0.91,1.06) |
| Suriname | 150 (99,222) | 403 (264,596) | 168.67 | 328.53 (277.50,387.39) | 345.99(312.74,382.29) | 0.19 (0.17,0.21) |
| Sweden | 7308 (4861,10680) | 10692 (7259,15544) | 46.31 | 327.15 (319.43,335.03) | 335.71(329.18,342.34) | 0.69 (-0.24,1.63) |
| Switzerland | 4650 (3217,6701) | 8215 (5752,11924) | 76.67 | 290.53 (282.15,299.11) | 293.27(286.88,299.76) | 0.02 (0.01,0.02) |
| Syrian Arab Republic | 1875 (1251,2741) | 6518 (4334,9584) | 247.63 | 206.48 (196.95,216.44) | 261.94(255.23,269.22) | 0.9 (0.84,0.96) |
| Taiwan (Province of China) | 7827 (4997,11863) | 25202 (16994,36970) | 221.99 | 275.61 (269.22,282.29) | 334.43(330.30,338.61) | 0.62 (0.6,0.64) |
| Tajikistan | 1680 (1120,2441) | 4553 (3059,6769) | 171.01 | 336.87 (320.66,353.99) | 382.23(370.44,394.59) | 0.52 (0.39,0.65) |
| Thailand | 12614 (8497,18503) | 55092 (37071,79186) | 336.75 | 209.58 (205.80,213.43) | 280.09(277.76,282.45) | 0.94 (0.92,0.97) |
| Timor-Leste | 82 (55,122) | 396 (269,571) | 382.93 | 185.86 (144.41,243.27) | 279.32(251.58,312.65) | 1.53 (1.41,1.65) |
| Togo | 409 (276,601) | 1729 (1164,2508) | 322.74 | 207.71 (186.87,232.75) | 266.76(253.14,281.90) | 0.85 (0.8,0.9) |
| Tokelau | 1 (0,1) | 1 (1,1) | 0.00 | 282.14 (0.91,6079.42) | 351.53(5.62,2756.69) | 0.78 (0.74,0.81) |
| Tonga | 26 (18,38) | 43 (29,62) | 65.38 | 271.06 (175.11,455.29) | 317.58(229.84,432.31) | 0.51 (0.5,0.52) |
| Trinidad and Tobago | 432 (289,618) | 1186 (797,1778) | 174.54 | 318.89 (288.25,361.95) | 337.53(318.52,357.47) | 0.18 (0.17,0.2) |
| Tunisia | 2053 (1360,3069) | 6733 (4504,10067) | 227.96 | 231.90 (221.07,244.50) | 282.65(275.88,289.58) | 0.61 (0.56,0.66) |
| Turkey | 11999 (7903,17657) | 43858 (29155,63106) | 265.51 | 197.32 (193.68,201.03) | 261.77(259.31,264.25) | 0.84 (0.38,1.31) |
| Turkmenistan | 1285 (854,1894) | 3605 (2373,5333) | 180.54 | 367.86 (347.50,389.57) | 448.61(433.60,464.12) | 0.78 (0.69,0.87) |
| Tuvalu | 3 (2,5) | 7 (4,10) | 133.33 | 283.25 (49.18,3981.80) | 351.35(131.64,1272.23) | 0.72 (0.68,0.75) |
| Uganda | 1937 (1318,2878) | 5487 (3670,7939) | 183.27 | 186.71 (177.90,196.49) | 227.79(221.50,234.33) | 0.6 (0.57,0.64) |
| Ukraine | 54890 (36617,77249) | 63479 (43476,89659) | 15.65 | 440.19 (436.44,443.99) | 476.30(472.57,480.06) | 0.33 (0.3,0.36) |
| United Arab Emirates | 147 (95,227) | 2029 (1245,3260) | 1280.27 | 246.02 (203.98,307.99) | 234.91(215.73,269.85) | -0.13 (-0.19,-0.07) |
| United Kingdom | 49178 (33735,69728) | 71884 (49641,102676) | 46.17 | 347.68 (344.57,350.83) | 358.02(355.37,360.68) | -0.4 (-0.72,-0.08) |
| United Republic of Tanzania | 4312 (2889,6429) | 10381 (6918,15070) | 140.75 | 236.39 (228.76,244.73) | 246.35(241.48,251.34) | -0.26 (-0.41,-0.11) |
| United States of America | 223083 (154121,322704) | 464284 (324079,665294) | 108.12 | 450.17 (448.28,452.07) | 479.25(477.86,480.64) | -0.06 (-0.36,0.25) |
| United States Virgin Islands | 47 (31,68) | 106 (73,157) | 125.53 | 322.79 (232.49,576.89) | 345.71(282.37,423.60) | 0.2 (0.18,0.22) |
| Uruguay | 2214 (1481,3177) | 2962 (2016,4235) | 33.79 | 334.81 (320.96,349.19) | 343.33(330.94,356.09) | 0.07 (0.05,0.09) |
| Uzbekistan | 7912 (5359,11620) | 23649 (15609,34839) | 198.90 | 381.67 (373.17,390.41) | 442.75(436.86,448.73) | 0.5 (0.44,0.55) |
| Vanuatu | 19 (13,28) | 68 (46,101) | 257.89 | 189.64 (103.87,649.70) | 232.42(175.40,364.45) | 0.63 (0.61,0.66) |
| Venezuela (Bolivarian Republic of) | 4704 (3163,6810) | 17185 (11707,24963) | 265.33 | 292.18 (283.81,300.84) | 320.77(315.96,325.64) | -0.15 (-0.39,0.09) |
| Viet Nam | 12879 (8720,18381) | 47112 (32037,68967) | 265.80 | 185.32 (182.09,188.61) | 262.64(260.21,265.09) | 1.19 (1.16,1.22) |
| Yemen | 1260 (848,1869) | 5169 (3398,7507) | 310.24 | 160.63 (150.93,171.56) | 225.04(218.68,231.67) | 1.36 (1.26,1.47) |
| Zambia | 1161 (770,1724) | 2869 (1907,4140) | 147.11 | 243.75 (228.78,261.97) | 257.68(247.61,268.72) | 0.24 (0.13,0.35) |
| Zimbabwe | 1417 (952,2076) | 2666 (1735,3925) | 88.14 | 214.64 (202.86,227.66) | 237.36(227.34,248.66) | 0.13 (0.05,0.2) |
