# Supplementary material for: Global burden of hand osteoarthritis in adults aged ≥55 years: An underestimated and fastest-growing subtype, 1990 to 2021 with projections to 2050: an analysis of GBD 2021 data
Source: Medicine (Baltimore). 2026 Aug 21;105(34):e50098. doi: 10.1097/MD.0000000000050098 (PMC13502859; doi:10.1097/MD.0000000000050098)
Supplement: Supplementary file 5 [file medi-105-e50098-s005.docx]

**Table S7. Decomposition analysis of the global burden of HOA in adults aged ≥55 years in 2021.**

| **Location** | **Measure** | **Overall difference** | **Aging** | **Population** | **Epidemiological change** |
| --- | --- | --- | --- | --- | --- |
| Low SDI | Incidence | 139551.05 | 407.76(0.29%) | 109451.22(78.43%) | 29692.07(21.28%) |
| Low-middle SDI | Incidence | 470944.54 | 1351.01(0.29%) | 377262.03(80.11%) | 92331.50(19.61%) |
| Middle SDI | Incidence | 1120592.71 | 4352.64(0.39%) | 867001.77(77.37%) | 249238.29(22.24%) |
| High-middle SDI | Incidence | 659959.94 | 19307.94(2.93%) | 562713.61(85.26%) | 77938.40(11.81%) |
| High SDI | Incidence | 644834.41 | 22470.02(3.48%) | 571780.65(88.67%) | 50583.74(7.84%) |
| Global | Incidence | 3327203.92 | -23470.24(-0.71%) | 2903217.16(87.26%) | 447457.00(13.45%) |
| Low SDI | Prevalence | 4841666.02 | 22802.84(0.47%) | 3774954.68(77.97%) | 1043908.50(21.56%) |
| Low-middle SDI | Prevalence | 13781649.33 | 279822.29(2.03%) | 9965320.85(72.31%) | 3536506.18(25.66%) |
| Middle SDI | Prevalence | 33138285.99 | 596404.07(1.80%) | 23507691.79(70.94%) | 9034190.14(27.26%) |
| High-middle SDI | Prevalence | 25282602.78 | 734443.51(2.90%) | 23586202.49(93.29%) | 961956.77(3.8%) |
| High SDI | Prevalence | 26728680.98 | 398868.33(1.49%) | 23007642.76(86.08%) | 3322169.88(12.43%) |
| Global | Prevalence | 118973328.4 | 707108.90(0.59%) | 102058717.94(85.78%) | 16207501.57(13.62%) |
| Low SDI | YLDs | 143738.82 | -781.35(-0.54%) | 113020.23(78.63%) | 31499.94(21.91%) |
| Low-middle SDI | YLDs | 552700.81 | -6024.75(-1.09%) | 415012.50(75.09%) | 143713.06(26%) |
| Middle SDI | YLDs | 1265012.17 | -10776.58(-0.85%) | 958216.15(75.75%) | 317572.60(25.1%) |
| High-middle SDI | YLDs | 508047.56 | 13871.88(2.73%) | 632171.49(124.43%) | -137995.80(-27.16%) |
| High SDI | YLDs | 700833.08 | 15009.12(2.14%) | 678104.74(96.76%) | 7719.21(1.1%) |
| Global | YLDs | 3568027.84 | -39796.98(-1.12%) | 3150919.72(88.31%) | 456905.09(12.81%) |
