# Supplementary material for: Global burden of hand osteoarthritis in adults aged ≥55 years: An underestimated and fastest-growing subtype, 1990 to 2021 with projections to 2050: an analysis of GBD 2021 data
Source: Medicine (Baltimore). 2026 Aug 21;105(34):e50098. doi: 10.1097/MD.0000000000050098 (PMC13502859; doi:10.1097/MD.0000000000050098)
Supplement: Supplementary file 6 [file medi-105-e50098-s006.docx]

**Table S8. Comparative analysis of the projected global burden of HOA in adults aged ≥55 years and general population from 1990 to 2050.**

| **Group** | **Measure** | **ASR_1990** | **ASR_2021** | **ASR_2050** | **EAPC(1990-2021)** | **Number_1990** | **Number_2021** | **Number_2050** |
| --- | --- | --- | --- | --- | --- | --- | --- | --- |
| ≥55 years | Incidence | 296.13 (295.71,296.54) | 344.20(343.90,344.50) | 402.38(237.48,567.28) | 0.43 (0.37,0.49) | 2008354 (1368574,2926073) | 5134309 (3514305,7415103) | 8991374(4923209，13059538) |
| ≥55 years | Prevalence | 9225.55 (9223.14,9227.96) | 10549.31(10547.64,10550.98) | 12082.39(9632.57，15155.26) | 0.49 (0.42,0.57) | 59357919 (43382064,77437231) | 154676897 (113502928,201841628) | 291920469(214860115，368980822) |
| ≥55 years | YLDs | 289.48 (289.05,289.90) | 330.77(330.48,331.07) | 373.62(333.78，413.46) | 0.5 (0.42,0.57) | 1872004 (856592,3869916) | 4858626 (2226493,10044723) | 8964535(6019316，11909754) |
| All ages | Incidence | 100.68(100.58，100.78) | 119.22(119.15，119.29) | 156.38(114.74，198.03) | 0.55 (0.49,0.6) | 4282225(2308344, 7004172) | 10367241(5631991, 16960394) | 18412584(14837948，21987220) |
| All ages | Prevalence | 1946.90(1946.46，1947.35) | 2240.15(2239.83，2240.46) | 2352.51(1862.39，2842.62) | 0.52 (0.45,0.59) | 76317645(54176416, 102583219) | 194284754(138360219, 259981168) | 353063674(276551161，429576186) |
| All ages | YLDs | 61.73(61.65，61.81) | 71.01(70.96，71.07) | 74.54(58.99，90.08) | 0.52 (0.45,0.6) | 2432664(1075695, 5043894) | 6167308(2738736, 12791788) | 11175296(8688034，13662558) |
