# Supplementary material for: Global burden of hand osteoarthritis in adults aged ≥55 years: An underestimated and fastest-growing subtype, 1990 to 2021 with projections to 2050: an analysis of GBD 2021 data
Source: Medicine (Baltimore). 2026 Aug 21;105(34):e50098. doi: 10.1097/MD.0000000000050098 (PMC13502859; doi:10.1097/MD.0000000000050098)
Supplement: Supplementary file 7 [file medi-105-e50098-s007.docx]

**Table S9. Comparative analysis of the global burden of HOA and other OA in adults aged ≥55 years from 1990 to 2050.**

| **Cause** | **Measure** | **ASR_1990** | **ASR_2021** | **ASR_2050** | **EAPC**  **(1990-2021)** | **EAPC**  **(2022-2050)** | **Number_1990** | **Number_2021** | **Number_2050** |  | **Percentage change**  **(2021/1990)** | **Percentage**  **change**  **(2050/2021)** |
| --- | --- | --- | --- | --- | --- | --- | --- | --- | --- | --- | --- | --- |
| HOA | Incidence | 296.13 (295.71,296.54) | 344.20(343.90,344.50) | 402.38(237.48,567.28) | 0.43 (0.37,0.49) | 0.54 (0.53,0.54) | 2008354 (1368574,2926073) | 5134309 (3514305,7415103) | 8991374(4923209，13059538) |  | 156.00% | 75.12% |
| Knee OA | Incidence | 985.68 (984.93,986.43) | 1046.80(1046.28,1047.32) | 1111.78(1052.15,1171.41) | 0.16 (0.13,0.2) | 0.19 (0.19,0.2) | 6762392 (5520055,8335917) | 15678582 (12817458,19181931) | 26890127(17460907，36319346) |  | 132.00% | 71.51% |
| Hip OA | Incidence | 65.49 (65.30,65.69) | 69.77(69.63,69.90) | 74.19(69.2,79.18) | 0.23 (0.2,0.26) | 0.2 (0.2,0.2) | 439053 (306776,619276) | 1037000 (739448,1461330) | 1661417(737760，2585074) |  | 136.00% | 60.21% |
| Other OA | Incidence | 131.78 (131.51,132.06) | 134.89(134.71,135.08) | 137.95(137.17,138.74) | 0.08 (0.08,0.08) | 0.08 (0.08,0.08) | 889235 (661838,1163701) | 2008248 (1506113,2611399) | 3418002(2571383，4264622) |  | 126.00% | 70.20% |
| HOA | Prevalence | 9225.55 (9223.14,9227.96) | 10549.31(10547.64,10550.98) | 12082.39(9632.57，15155.26) | 0.49 (0.42,0.57) | 0.44(0.43,0.44) | 59357919 (43382064,77437231) | 154676897 (113502928,201841628) | 291920469(214860115，368980822) |  | 160.58% | 88.73% |
| Knee OA | Prevalence | 17359.32 (17356.09,17362.56) | 18716.67(18714.45,18718.88) | 19812.05(18991.31，20632.79) | 0.27 (0.26,0.29) | 0.2 (0.2,0.2) | 114369351 (96720634,132084037) | 276356905 (234725518,318293530) | 489221448(380629722，597813173) |  | 141.64% | 77.03% |
| Hip OA | Prevalence | 1792.88 (1791.81,1793.95) | 1884.41(1883.70,1885.12) | 1889.99(1755.71，2024.27) | 0.22 (0.2,0.25) | 0 (0,0) | 11392418 (8622342,14557724) | 27494279 (20880072,35229429) | 45268855(18484452，72053259) |  | 141.34% | 64.65% |
| Other OA | Prevalence | 3243.35 (3241.93,3244.78) | 3420.08(3419.13,3421.03) | 3589.66(3568.48，3610.85) | 0.17 (0.17,0.18) | 0.16 (0.16,0.17) | 20834170 (16112609,27273288) | 50073588 (38914957,65416741) | 86956847(70822825，103090870) |  | 140.34% | 73.66% |
| HOA | YLDs | 289.48 (289.05,289.90) | 330.77(330.48,331.07) | 373.62(333.78，413.46) | 0.5 (0.42,0.57) | 0.41 (0.41,0.42) | 1872004 (856592,3869916) | 4858626 (2226493,10044723) | 8964535(6019316，11909754) |  | 159.54% | 84.51% |
| Knee OA | YLDs | 549.68 (549.11,550.26) | 591.87(591.47,592.26) | 624.28(598.20，650.36) | 0.28 (0.26,0.29) | 0.19 (0.19,0.19) | 3636056 (1778623,7099267) | 8752241 (4293589,17096055) | 15306885(11920748，18693022) |  | 140.71% | 74.89% |
| Hip OA | YLDs | 56.15 (55.96,56.33) | 58.91(58.78,59.03) | 59.08(54.81，63.34) | 0.22 (0.2,0.25) | 0 (0,0) | 358754 (170182,730537) | 861216 (407598,1753757) | 1392344(550911，2233778) |  | 140.06% | 61.67% |
| Other OA | YLDs | 102.06 (101.81,102.31) | 107.58(107.41,107.75) | 112.91(111.73，114.10) | 0.18 (0.17,0.18) | 0.17 (0.16,0.17) | 659149 (300224,1415264) | 1578117 (722028,3381071) | 2771344(2265740，3276948) |  | 139.42% | 75.61% |
