# Supplementary material for: Global burden of hand osteoarthritis in adults aged ≥55 years: An underestimated and fastest-growing subtype, 1990 to 2021 with projections to 2050: an analysis of GBD 2021 data
Source: Medicine (Baltimore). 2026 Aug 21;105(34):e50098. doi: 10.1097/MD.0000000000050098 (PMC13502859; doi:10.1097/MD.0000000000050098)
Supplement: Supplementary file 8 [file medi-105-e50098-s008.docx]

**Table S5. The prevalence of HOA cases and rate in adults aged ≥55 years across 204 countries in 1990 and 2021, and the trends from 1990 to 2021.**

| **location** | **Prevalence Cases** | | **Percentage change**  **(100%)** | **ASPR** | | **EAPC (95%CI)** |
| --- | --- | --- | --- | --- | --- | --- |
|  | **1990 (95% UI)** | **2021 (95% UI)** |  | **1990(95%CI)** | **2021(95%CI)** |  |
| Afghanistan | 47050 (33509,62551) | 61154 (44205,80402) | 29.98 | 4181.96 (4139.92,4224.98) | 5186.54(5142.96,5230.75) | 0.67 (0.57,0.78) |
| Albania | 22495 (16430,29392) | 73085 (53941,94329) | 224.89 | 6866.93 (6775.26,6959.73) | 9329.67(9261.60,9398.25) | 1.22 (1.14,1.3) |
| Algeria | 134784 (97407,179036) | 548243 (397140,718482) | 306.76 | 6689.33 (6642.56,6738.31) | 9332.52(9305.95,9359.35) | 1.06 (0.97,1.14) |
| American Samoa | 359 (261,473) | 918 (668,1197) | 155.71 | 10537.28 (9364.84,12061.00) | 11534.10(10769.61,12375.22) | 0.28 (0.27,0.29) |
| Andorra | 939 (683,1227) | 2646 (1945,3436) | 181.79 | 9639.27 (8983.91,10515.71) | 9969.34(9590.90,10359.33) | 0.12 (0.1,0.13) |
| Angola | 67023 (48158,88940) | 234135 (169713,301400) | 249.34 | 11753.53 (11648.72,11860.35) | 13219.54(13158.38,13281.28) | 0.38 (0.37,0.4) |
| Antigua and Barbuda | 1036 (759,1340) | 2241 (1661,2929) | 116.31 | 11522.64 (10821.09,12261.16) | 12272.52(11760.85,12809.35) | 0.17 (0.15,0.19) |
| Argentina | 640610 (469003,830521) | 1135848 (832206,1462089) | 77.31 | 11625.41 (11596.67,11654.23) | 11942.20(11920.17,11964.25) | 0.06 (0.04,0.07) |
| Armenia | 56662 (41297,73257) | 137085 (101711,176974) | 141.93 | 12447.61 (12340.01,12556.14) | 17506.77(17412.26,17601.80) | 1.33 (1.22,1.43) |
| Australia | 367924 (276827,468030) | 883615 (647389,1146459) | 140.16 | 11054.32 (11018.28,11090.48) | 11493.30(11469.05,11517.60) | 0.13 (0.12,0.14) |
| Austria | 198469 (145006,255234) | 307274 (226539,395637) | 54.82 | 9839.22 (9794.86,9883.80) | 10001.03(9965.07,10037.09) | 0.04 (0.03,0.05) |
| Azerbaijan | 132535 (97307,173872) | 322322 (237677,413537) | 143.20 | 16257.94 (16166.21,16350.42) | 18084.29(18016.03,18152.83) | 0.49 (0.32,0.66) |
| Bahamas | 2798 (2053,3650) | 8494 (6224,11225) | 203.57 | 11643.29 (11211.53,12090.44) | 12353.75(12086.74,12625.97) | 0.18 (0.17,0.2) |
| Bahrain | 2449 (1775,3278) | 14250 (10332,18973) | 481.87 | 9730.84 (9198.65,10430.57) | 10035.50(9803.54,10304.10) | 0.07 (0.04,0.09) |
| Bangladesh | 438784 (314347,582220) | 1847821 (1336670,2418176) | 321.12 | 6048.59 (6030.23,6067.03) | 8123.93(8111.93,8135.95) | 0.98 (0.86,1.1) |
| Barbados | 5526 (4067,7145) | 11285 (8265,14737) | 104.22 | 11205.37 (10900.15,11520.86) | 12327.39(12100.56,12557.78) | 0.31 (0.28,0.35) |
| Belarus | 332722 (246871,430892) | 506344 (374372,648781) | 52.18 | 14639.75 (14589.21,14690.46) | 17613.17(17564.20,17662.26) | 0.7 (0.66,0.73) |
| Belgium | 271325 (197618,350695) | 407009 (300747,525477) | 50.01 | 10155.78 (10117.06,10194.66) | 10290.61(10258.40,10322.91) | 0.04 (0.03,0.05) |
| Belize | 1284 (949,1662) | 5069 (3740,6524) | 294.78 | 8365.96 (7914.15,8837.39) | 10535.43(10242.78,10834.96) | 0.67 (0.61,0.72) |
| Benin | 17779 (12715,23570) | 65210 (47536,85647) | 266.78 | 5720.02 (5634.80,5806.63) | 8379.46(8313.01,8446.52) | 1.24 (1.23,1.25) |
| Bermuda | 1235 (911,1614) | 2975 (2187,3836) | 140.89 | 11849.23 (11190.85,12548.88) | 12455.30(12009.17,12914.50) | 0.16 (0.15,0.17) |
| Bhutan | 1956 (1401,2603) | 6950 (5033,9048) | 255.32 | 5538.78 (5270.34,5828.22) | 7123.99(6956.50,7295.35) | 0.76 (0.73,0.78) |
| Bolivia (Plurinational State of) | 42351 (30779,55835) | 152710 (111126,199136) | 260.58 | 8462.38 (8378.25,8547.80) | 10212.82(10160.21,10265.78) | 0.6 (0.49,0.71) |
| Bosnia and Herzegovina | 63653 (46448,83127) | 143944 (106694,187176) | 126.14 | 9395.00 (9316.75,9474.23) | 13017.97(12950.36,13085.90) | 1.22 (1.05,1.39) |
| Botswana | 7363 (5357,9776) | 26253 (19205,34320) | 256.55 | 8530.80 (8304.89,8772.28) | 11615.03(11465.63,11767.13) | 0.9 (0.83,0.97) |
| Brazil | 1442140 (1047825,1892000) | 5264023 (3856447,6840527) | 265.01 | 10231.88 (10214.57,10249.23) | 12355.43(12344.85,12366.02) | 0.61 (0.59,0.63) |
| Brunei Darussalam | 1942 (1428,2520) | 7651 (5611,9996) | 293.98 | 12961.08 (12348.24,13670.04) | 13731.05(13363.02,14176.33) | 0.17 (0.15,0.18) |
| Bulgaria | 274586 (198723,355958) | 371512 (275591,478095) | 35.30 | 12423.97 (12364.38,12484.81) | 15018.26(14968.48,15068.28) | 0.66 (0.6,0.72) |
| Burkina Faso | 28024 (19791,37506) | 66099 (47269,88876) | 135.87 | 4199.80 (4144.91,4256.13) | 4724.22(4686.37,4762.47) | 0.38 (0.35,0.41) |
| Burundi | 19669 (14223,26482) | 42261 (30490,56052) | 114.86 | 5539.80 (5454.10,5628.79) | 5844.40(5783.34,5906.47) | 0.24 (0.19,0.3) |
| Cabo Verde | 2234 (1620,2938) | 6505 (4776,8471) | 191.18 | 5596.40 (5364.63,5836.40) | 8880.29(8660.66,9104.67) | 1.66 (1.61,1.72) |
| Cambodia | 27965 (19811,37621) | 130411 (94243,171322) | 366.34 | 4165.68 (4111.65,4221.02) | 6542.79(6504.21,6581.77) | 1.58 (1.53,1.64) |
| Cameroon | 36094 (25987,48195) | 140886 (101375,185525) | 290.33 | 5324.28 (5265.45,5384.13) | 7504.82(7462.75,7547.27) | 1.06 (0.98,1.14) |
| Canada | 691903 (510156,893233) | 1601163 (1179922,2064169) | 131.41 | 12503.31 (12473.72,12532.95) | 12715.10(12695.27,12734.94) | 0.05 (0.05,0.06) |
| Central African Republic | 11485 (8194,15282) | 22387 (16099,29640) | 94.92 | 6935.21 (6775.55,7101.72) | 7274.67(7150.01,7406.29) | 0.07 (0.01,0.12) |
| Chad | 21165 (15237,28100) | 43857 (31555,58066) | 107.21 | 4758.71 (4692.04,4826.67) | 5129.33(5077.95,5181.48) | 0.22 (0.17,0.27) |
| Chile | 161279 (117838,209115) | 496149 (362435,638516) | 207.63 | 9784.48 (9736.12,9833.15) | 11088.99(11058.14,11119.91) | 0.39 (0.37,0.4) |
| China | 6146841 (4331490,8360686) | 27880054 (20121293,36885010) | 353.57 | 4764.73 (4760.19,4769.27) | 7643.31(7640.41,7646.21) | 1.53 (1.33,1.74) |
| Colombia | 255139 (184255,331347) | 1076962 (795081,1396741) | 322.11 | 9189.77 (9153.58,9226.09) | 11326.38(11304.92,11347.87) | 0.78 (0.74,0.83) |
| Comoros | 1638 (1171,2184) | 5885 (4288,7720) | 259.28 | 5520.44 (5221.59,5857.29) | 7699.25(7496.80,7908.51) | 1.21 (1.14,1.28) |
| Congo | 16261 (11757,21530) | 45079 (32470,59140) | 177.22 | 9832.23 (9628.73,10054.35) | 11319.76(11201.65,11441.08) | 0.42 (0.36,0.48) |
| Cook Islands | 198 (143,260) | 536 (394,691) | 170.71 | 9778.98 (8403.18,11611.51) | 11569.84(10606.67,12617.27) | 0.54 (0.51,0.56) |
| Costa Rica | 25323 (18474,32764) | 104788 (77474,135768) | 313.81 | 8999.14 (8888.45,9110.97) | 11032.68(10965.74,11099.94) | 0.67 (0.64,0.7) |
| Croatia | 137989 (102160,178210) | 220836 (164039,284046) | 60.04 | 12952.47 (12881.32,13024.25) | 14267.30(14206.87,14327.99) | 0.39 (0.37,0.42) |
| Cuba | 152653 (111586,199067) | 356644 (261323,459775) | 133.63 | 8954.47 (8907.95,9001.69) | 10446.34(10411.88,10480.88) | 0.49 (0.46,0.51) |
| Cyprus | 12464 (9135,16268) | 35655 (26141,46238) | 186.06 | 8975.55 (8667.68,9778.97) | 10055.86(9942.33,10176.16) | 0.35 (0.29,0.41) |
| Czechia | 348792 (255718,449794) | 563040 (413782,726181) | 61.43 | 14641.23 (14591.46,14691.23) | 15443.83(15402.48,15485.28) | 0.15 (0.12,0.19) |
| C么te d'Ivoire | 32418 (23351,43621) | 122722 (88187,162042) | 278.56 | 5708.27 (5636.69,5781.40) | 7504.75(7459.32,7550.57) | 0.83 (0.77,0.9) |
| Democratic People's Republic of Korea | 120692 (86807,161059) | 355200 (259976,465030) | 194.30 | 4973.17 (4943.32,5003.24) | 6557.54(6535.43,6579.75) | 0.87 (0.83,0.91) |
| Democratic Republic of the Congo | 182654 (131488,240741) | 434344 (312962,575921) | 137.80 | 7564.13 (7521.36,7607.39) | 8047.25(8020.52,8074.11) | 0.03 (-0.17,0.22) |
| Denmark | 172998 (130643,221453) | 201228 (147821,260706) | 16.32 | 12752.00 (12690.76,12813.48) | 9998.38(9953.77,10043.16) | -0.48 (-0.6,-0.37) |
| Djibouti | 1162 (829,1565) | 8552 (6208,11280) | 635.97 | 6217.61 (5798.60,6693.70) | 9178.99(8951.84,9418.37) | 1.52 (1.39,1.65) |
| Dominica | 888 (654,1149) | 1540 (1122,2006) | 73.42 | 8871.43 (8288.12,9499.19) | 10548.37(10023.01,11097.44) | 0.55 (0.5,0.61) |
| Dominican Republic | 58460 (42512,76184) | 186339 (136625,240736) | 218.75 | 9881.80 (9799.66,9964.77) | 11272.04(11220.86,11323.43) | 0.4 (0.39,0.41) |
| Ecuador | 80822 (59508,105490) | 301999 (223368,390322) | 273.66 | 9629.24 (9562.16,9696.99) | 11024.72(10985.26,11064.32) | 0.45 (0.42,0.48) |
| Egypt | 247529 (178565,330625) | 787136 (569409,1037002) | 218.00 | 6005.06 (5978.10,6032.37) | 7640.83(7621.67,7660.06) | 0.59 (0.53,0.66) |
| El Salvador | 42645 (31214,55814) | 112875 (83665,145577) | 164.69 | 8837.34 (8753.45,8921.86) | 10866.80(10803.14,10930.74) | 0.73 (0.72,0.75) |
| Equatorial Guinea | 1636 (1177,2175) | 8448 (6136,11061) | 416.38 | 5699.96 (5380.07,6065.63) | 11428.55(11168.73,11698.17) | 2.69 (2.52,2.86) |
| Eritrea | 7616 (5455,10117) | 27429 (19705,36534) | 260.15 | 5235.73 (5071.31,5414.51) | 7036.49(6935.35,7142.54) | 0.99 (0.96,1.02) |
| Estonia | 56918 (41795,73058) | 84953 (63080,109653) | 49.26 | 15991.80 (15858.45,16126.65) | 18586.69(18458.69,18715.44) | 0.47 (0.39,0.54) |
| Eswatini | 2990 (2173,3953) | 8802 (6429,11489) | 194.38 | 7177.00 (6900.91,7471.50) | 10495.52(10227.47,10793.18) | 1.23 (1.19,1.27) |
| Ethiopia | 189136 (135359,251649) | 783010 (571217,1024561) | 313.99 | 6659.11 (6616.96,6702.15) | 11979.48(11952.06,12006.99) | 2.23 (2.14,2.33) |
| Fiji | 4325 (3124,5704) | 13830 (10078,18377) | 219.77 | 8371.01 (8096.10,8660.48) | 10946.10(10738.50,11166.21) | 0.93 (0.88,0.97) |
| Finland | 116002 (85719,150758) | 214488 (157946,274971) | 84.90 | 9540.03 (9484.66,9595.71) | 10012.34(9968.51,10056.33) | 0.15 (0.12,0.17) |
| France | 1429384 (1053598,1857576) | 2394420 (1773375,3096772) | 67.51 | 10004.15 (9987.38,10020.95) | 10206.68(10193.34,10220.04) | 0.06 (0.05,0.07) |
| Gabon | 7398 (5389,9733) | 17231 (12566,22587) | 132.91 | 7960.50 (7770.91,8157.13) | 10476.24(10307.90,10648.88) | 0.83 (0.78,0.88) |
| Gambia | 3124 (2235,4136) | 13378 (9656,17624) | 328.23 | 6085.95 (5851.11,6342.75) | 9073.51(8914.70,9236.64) | 1.36 (1.32,1.39) |
| Georgia | 148131 (107964,191988) | 170065 (126058,219268) | 14.81 | 13786.40 (13714.98,13858.22) | 15931.22(15854.71,16008.05) | 0.34 (0.17,0.51) |
| Germany | 2193183 (1610540,2856087) | 3372782 (2507034,4355281) | 53.78 | 10187.28 (10173.59,10200.98) | 10233.26(10222.06,10244.47) | -0.14 (-0.2,-0.08) |
| Ghana | 106044 (77491,140874) | 254436 (186099,333555) | 139.93 | 11064.79 (10992.45,11137.99) | 9957.98(9916.56,9999.67) | -0.18 (-0.6,0.25) |
| Greece | 209236 (153373,274535) | 362855 (263460,466399) | 73.42 | 7838.97 (7804.92,7873.23) | 9209.21(9177.90,9240.60) | 2.03 (1.45,2.6) |
| Greenland | 586 (422,770) | 1542 (1126,2010) | 163.14 | 11565.57 (10509.00,13124.05) | 12249.96(11592.54,12962.22) | 0.18 (0.17,0.19) |
| Grenada | 1179 (861,1524) | 2143 (1571,2809) | 81.76 | 9395.55 (8859.72,9957.13) | 11252.74(10761.00,11768.39) | 0.56 (0.49,0.63) |
| Guam | 1229 (886,1633) | 4401 (3248,5730) | 258.10 | 10306.08 (9625.63,11241.40) | 11755.63(11406.24,12113.81) | 0.41 (0.38,0.44) |
| Guatemala | 41593 (30212,54703) | 164966 (120624,214937) | 296.62 | 7703.36 (7621.25,7788.42) | 9176.00(9130.63,9221.88) | 0.55 (0.53,0.56) |
| Guinea | 24164 (17339,32512) | 48669 (35077,64688) | 101.41 | 4552.43 (4493.24,4612.59) | 5632.68(5581.36,5684.51) | 0.64 (0.6,0.68) |
| Guinea-Bissau | 3032 (2182,4069) | 6823 (4935,9034) | 125.03 | 5184.90 (4969.79,5423.06) | 6623.35(6438.62,6821.71) | 0.78 (0.73,0.83) |
| Guyana | 5206 (3762,6828) | 11743 (8630,15380) | 125.57 | 8810.89 (8567.43,9063.42) | 10980.14(10775.05,11189.50) | 0.7 (0.67,0.73) |
| Haiti | 28907 (21052,37872) | 84108 (61500,109934) | 190.96 | 5838.64 (5762.67,5916.71) | 7620.13(7564.50,7676.46) | 0.9 (0.88,0.92) |
| Honduras | 25547 (18550,33244) | 94968 (69131,123924) | 271.74 | 7980.99 (7878.75,8086.38) | 9334.71(9273.17,9396.94) | 0.53 (0.49,0.58) |
| Hungary | 358657 (264760,462842) | 520036 (385585,671200) | 45.00 | 13957.56 (13910.39,14005.00) | 15612.11(15568.64,15655.68) | 0.3 (0.22,0.37) |
| Iceland | 5682 (4274,7216) | 10569 (7840,13731) | 86.01 | 11680.61 (11377.64,11989.93) | 10589.94(10387.31,10795.70) | -0.4 (-0.61,-0.19) |
| India | 4850285 (3473359,6452117) | 19307442 (14050518,25288127) | 298.07 | 7002.73 (6995.79,7009.68) | 10021.77(10017.15,10026.38) | 1.19 (1.14,1.25) |
| Indonesia | 978096 (701063,1292845) | 3691647 (2678752,4864294) | 277.43 | 6713.45 (6699.08,6727.86) | 9708.02(9697.08,9718.99) | 1.11 (1.05,1.16) |
| Iran (Islamic Republic of) | 314999 (226516,418913) | 1234212 (903957,1615128) | 291.81 | 7710.73 (7679.50,7742.11) | 9896.88(9879.17,9914.62) | 0.92 (0.63,1.22) |
| Iraq | 88747 (63761,116214) | 309676 (226899,408301) | 248.94 | 7183.00 (7135.65,7230.59) | 8513.95(8482.42,8545.61) | 0.34 (0.26,0.41) |
| Ireland | 64485 (47204,83358) | 133555 (97515,173740) | 107.11 | 9267.13 (9194.14,9340.85) | 9939.46(9885.93,9993.23) | 0.21 (0.17,0.25) |
| Israel | 81534 (59574,105865) | 213523 (156140,277317) | 161.88 | 9892.72 (9823.15,9963.07) | 10313.42(10269.13,10357.87) | -1.47 (-2.09,-0.84) |
| Italy | 1664023 (1219748,2172141) | 2766819 (2038516,3552427) | 66.27 | 10756.57 (10739.98,10773.19) | 11351.07(11337.21,11364.95) | 0.53 (0.25,0.8) |
| Jamaica | 27711 (20287,35905) | 55593 (41230,71816) | 100.62 | 9167.48 (9059.16,9276.83) | 10465.77(10378.13,10553.99) | 0.41 (0.38,0.44) |
| Japan | 3540552 (2594745,4587471) | 8586002 (6440704,10949904) | 142.50 | 12157.20 (12144.41,12170.01) | 14515.91(14505.17,14526.65) | 1.33 (0.65,2.01) |
| Jordan | 16588 (11985,21851) | 115627 (84704,150489) | 597.05 | 8335.17 (8201.21,8471.58) | 9839.69(9779.36,9900.48) | 0.52 (0.48,0.56) |
| Kazakhstan | 328147 (240326,426196) | 653889 (490873,829941) | 99.27 | 16191.75 (16135.16,16248.53) | 21580.69(21525.38,21636.24) | 1.02 (0.94,1.1) |
| Kenya | 102674 (74304,136272) | 412667 (300850,541071) | 301.92 | 8191.90 (8139.06,8245.31) | 11926.20(11886.73,11965.85) | 1.25 (1.22,1.29) |
| Kiribati | 534 (385,703) | 1201 (870,1576) | 124.91 | 9607.38 (8710.23,10698.01) | 10572.28(9883.55,11392.64) | 0.2 (0.15,0.25) |
| Kuwait | 7558 (5523,10116) | 42160 (30868,56070) | 457.82 | 9063.67 (8847.08,9285.29) | 9759.74(9659.79,9860.68) | 0.33 (0.29,0.37) |
| Kyrgyzstan | 75126 (55122,97231) | 145168 (107364,188651) | 93.23 | 15162.45 (15051.36,15274.60) | 18234.67(18134.80,18335.15) | 0.82 (0.65,0.99) |
| Lao People's Democratic Republic | 14468 (10344,19443) | 47992 (34826,63107) | 231.71 | 4631.08 (4541.13,4725.85) | 6737.25(6673.63,6801.63) | 1.24 (1.15,1.32) |
| Latvia | 92649 (68740,119103) | 122527 (91064,157208) | 32.25 | 14893.77 (14796.94,14991.31) | 17921.31(17819.11,18023.99) | 0.67 (0.61,0.73) |
| Lebanon | 27018 (19726,35493) | 101441 (74794,131014) | 275.46 | 7575.28 (7481.79,7670.04) | 10154.99(10092.16,10218.13) | 0.97 (0.93,1.01) |
| Lesotho | 9169 (6650,12025) | 17619 (12814,23040) | 92.16 | 6867.34 (6722.42,7016.62) | 10436.66(10260.52,10620.70) | 1.53 (1.42,1.65) |
| Liberia | 10233 (7377,13564) | 23083 (16778,30352) | 125.57 | 5634.58 (5517.86,5755.48) | 7570.13(7467.80,7674.12) | 1.13 (1.06,1.2) |
| Libya | 23865 (17395,31684) | 76155 (55334,99438) | 219.11 | 8004.33 (7901.48,8108.29) | 9628.02(9557.92,9698.56) | 0.58 (0.5,0.66) |
| Lithuania | 112923 (83583,145757) | 176742 (131791,225891) | 56.52 | 14374.51 (14289.79,14459.76) | 17691.73(17607.98,17775.79) | 0.79 (0.73,0.84) |
| Luxembourg | 9495 (7058,12271) | 18441 (13557,23917) | 94.22 | 10079.66 (9874.53,10290.63) | 10217.49(10069.83,10366.81) | 0.02 (0,0.04) |
| Madagascar | 35277 (25300,46785) | 84037 (60548,111901) | 138.22 | 4530.22 (4479.89,4581.31) | 5200.57(5159.73,5241.97) | 0.41 (0.39,0.43) |
| Malawi | 29755 (21339,39756) | 72121 (52149,95211) | 142.38 | 5147.77 (5081.85,5215.26) | 6647.40(6594.88,6700.52) | 0.88 (0.83,0.93) |
| Malaysia | 115771 (84318,151467) | 501076 (366108,656220) | 332.82 | 8143.21 (8095.81,8190.84) | 10594.11(10564.05,10624.27) | 0.77 (0.7,0.84) |
| Maldives | 1175 (843,1567) | 5673 (4143,7473) | 382.81 | 8483.71 (7908.97,9231.23) | 11104.76(10808.26,11409.07) | 0.71 (0.62,0.81) |
| Mali | 29720 (21245,40146) | 85494 (61687,114803) | 187.66 | 4891.43 (4828.84,4955.49) | 6366.95(6319.51,6415.31) | 0.87 (0.83,0.92) |
| Malta | 7333 (5361,9538) | 17185 (12692,22227) | 134.35 | 10145.15 (9911.42,10385.42) | 10307.95(10149.48,10468.60) | 0.03 (0.01,0.05) |
| Marshall Islands | 248 (181,321) | 631 (456,827) | 154.44 | 10142.38 (8746.97,12655.06) | 11782.44(10651.35,13332.07) | 0.48 (0.45,0.51) |
| Mauritania | 9082 (6560,12106) | 26824 (19235,35003) | 195.35 | 5759.15 (5632.79,5891.71) | 7805.37(7709.87,7902.15) | 0.97 (0.87,1.07) |
| Mauritius | 10651 (7746,14068) | 37569 (27484,48798) | 252.73 | 9174.39 (8992.66,9361.14) | 11432.90(11315.88,11550.94) | 0.72 (0.69,0.74) |
| Mexico | 700551 (511974,912770) | 2929205 (2188306,3762511) | 318.13 | 10444.18 (10418.87,10469.61) | 13885.54(13869.56,13901.54) | 1.11 (1.02,1.21) |
| Micronesia (Federated States of) | 654 (474,856) | 1302 (946,1713) | 99.08 | 8525.48 (7838.99,9352.17) | 10905.85(10243.22,11643.09) | 0.84 (0.8,0.89) |
| Monaco | 1191 (874,1547) | 1680 (1227,2163) | 41.06 | 10073.36 (9485.45,10693.38) | 10168.44(9674.05,10683.38) | 0.03 (0.02,0.04) |
| Mongolia | 21782 (16101,28209) | 76654 (56624,99988) | 251.91 | 13058.53 (12880.08,13240.34) | 20843.38(20680.32,21010.24) | 1.72 (1.63,1.82) |
| Montenegro | 14029 (10262,18217) | 25580 (18958,33440) | 82.34 | 13266.08 (13044.04,13491.45) | 14742.25(14557.48,14930.79) | 0.42 (0.39,0.45) |
| Morocco | 177292 (129260,233603) | 471197 (346629,609897) | 165.77 | 7788.59 (7751.74,7825.63) | 8206.07(8181.96,8230.29) | -0.03 (-0.16,0.1) |
| Mozambique | 43540 (31244,57536) | 103964 (74522,137373) | 138.78 | 4900.66 (4849.23,4953.20) | 6417.91(6374.65,6461.61) | 0.87 (0.82,0.92) |
| Myanmar | 187023 (133885,247255) | 668199 (482712,868927) | 257.28 | 5217.83 (5191.71,5244.19) | 8392.19(8371.42,8413.02) | 1.71 (1.63,1.79) |
| Namibia | 6980 (5030,9194) | 20242 (14690,26328) | 190.00 | 6955.54 (6770.76,7148.22) | 9579.75(9438.89,9723.96) | 0.94 (0.86,1.02) |
| Nauru | 62 (44,81) | 98 (71,128) | 58.06 | 9211.16 (6462.49,23971.67) | 11325.84(8921.66,15955.91) | 0.73 (0.66,0.8) |
| Nepal | 63476 (45826,85871) | 230026 (165982,306406) | 262.38 | 4544.00 (4505.58,4582.94) | 6093.95(6068.06,6119.99) | 0.89 (0.84,0.95) |
| Netherlands | 365114 (301085,422850) | 611943 (451568,795505) | 67.60 | 10790.76 (10755.52,10826.09) | 10112.35(10086.73,10138.02) | -0.58 (-0.96,-0.19) |
| New Zealand | 78405 (57552,101530) | 182349 (134301,234917) | 132.57 | 11698.37 (11615.78,11781.54) | 12440.07(12382.75,12497.60) | 0.18 (0.17,0.2) |
| Nicaragua | 17411 (12783,22708) | 75376 (55073,98692) | 332.92 | 7288.88 (7179.61,7399.63) | 9517.24(9448.70,9586.38) | 0.88 (0.87,0.89) |
| Niger | 18242 (12921,24574) | 64096 (45937,84492) | 251.36 | 4617.58 (4540.29,4697.71) | 5080.78(5037.03,5125.14) | 0.34 (0.3,0.39) |
| Nigeria | 499045 (359172,658525) | 1278278 (923094,1685501) | 156.14 | 7350.61 (7329.24,7372.06) | 9268.01(9251.16,9284.89) | 0.98 (0.82,1.13) |
| Niue | 37 (27,48) | 44 (32,57) | 18.92 | 9894.62 (6943.49,13820.88) | 11569.86(8377.26,16093.00) | 0.54 (0.51,0.56) |
| North Macedonia | 34037 (24999,44502) | 75901 (54911,98598) | 123.00 | 10792.55 (10675.64,10910.56) | 13071.46(12947.35,13212.55) | 0.73 (0.68,0.78) |
| Northern Mariana Islands | 230 (168,303) | 1003 (729,1312) | 336.09 | 10532.37 (8947.33,13738.14) | 11364.16(10576.11,12248.40) | 0.19 (0.17,0.2) |
| Norway | 133447 (98316,172195) | 201076 (148387,260427) | 50.68 | 11530.27 (11466.45,11594.41) | 11843.94(11791.36,11896.71) | 0.34 (-0.01,0.69) |
| Oman | 8179 (5909,10743) | 28504 (20738,37383) | 248.50 | 8618.73 (8425.09,8817.82) | 10091.34(9962.20,10222.94) | 0.46 (0.43,0.5) |
| Pakistan | 641929 (466131,843625) | 1808875 (1305678,2365558) | 181.79 | 7313.77 (7295.24,7332.35) | 9820.52(9805.45,9835.62) | 0.9 (0.86,0.95) |
| Palau | 153 (110,200) | 444 (323,592) | 190.20 | 9879.68 (8248.30,12356.67) | 11496.30(10303.64,13168.17) | 0.48 (0.46,0.51) |
| Palestine | 9484 (6904,12451) | 37612 (27620,49556) | 296.58 | 6821.28 (6681.30,6964.65) | 9468.02(9366.08,9571.66) | 1.05 (1.01,1.09) |
| Panama | 21507 (15761,28016) | 81202 (59905,104347) | 277.56 | 8989.27 (8869.06,9110.87) | 10904.45(10829.45,10979.85) | 0.59 (0.58,0.61) |
| Papua New Guinea | 13361 (9570,17718) | 48559 (35176,64435) | 263.44 | 5094.42 (4984.48,5209.38) | 6624.50(6558.04,6691.97) | 0.86 (0.83,0.89) |
| Paraguay | 37357 (27496,48471) | 101169 (74023,131669) | 170.82 | 10479.43 (10372.94,10586.82) | 10461.17(10396.33,10526.33) | -0.05 (-0.26,0.16) |
| Peru | 200536 (147115,261172) | 644551 (472957,840756) | 221.41 | 10459.42 (10413.41,10505.60) | 11597.08(11568.74,11625.47) | 0.2 (0.07,0.32) |
| Philippines | 376688 (271819,496149) | 1392998 (1012497,1820644) | 269.80 | 8469.01 (8434.83,8503.86) | 10680.87(10662.45,10699.31) | 0.59 (0.52,0.66) |
| Poland | 995152 (731563,1292220) | 2014277 (1502634,2568823) | 102.41 | 13168.98 (13142.58,13195.44) | 16059.12(16036.62,16081.65) | 0.69 (0.63,0.75) |
| Portugal | 218167 (158942,284408) | 402117 (296985,521124) | 84.32 | 8946.78 (8908.32,8985.49) | 9745.78(9714.74,9776.90) | 0.93 (0.52,1.34) |
| Puerto Rico | 69923 (51094,90937) | 152495 (113143,195730) | 118.09 | 11441.49 (11355.61,11528.15) | 12351.64(12287.79,12415.76) | 0.25 (0.24,0.26) |
| Qatar | 1227 (883,1646) | 12203 (8878,16055) | 894.54 | 8864.78 (8254.58,9619.93) | 9594.12(9365.50,9833.97) | 0.2 (0.16,0.24) |
| Republic of Korea | 532479 (390828,689437) | 2173906 (1608189,2817021) | 308.26 | 11477.96 (11443.89,11512.37) | 13080.68(13063.25,13098.13) | 0.39 (0.23,0.56) |
| Republic of Moldova | 84524 (61829,109482) | 148890 (110140,190618) | 76.15 | 11357.37 (11276.53,11439.25) | 14088.72(14016.83,14160.90) | 0.92 (0.77,1.06) |
| Romania | 522126 (383307,678255) | 773591 (576535,1001198) | 48.16 | 10783.67 (10752.09,10815.50) | 12222.69(12194.68,12250.76) | 0.51 (0.46,0.55) |
| Russian Federation | 6133030 (4524653,7894183) | 8312532 (6153567,10693606) | 35.54 | 19885.51 (19869.18,19901.85) | 19492.71(19479.20,19506.23) | 0.21 (0.12,0.3) |
| Rwanda | 18862 (13362,25396) | 53757 (38894,71379) | 185.00 | 4475.90 (4401.50,4552.85) | 5602.76(5551.01,5655.27) | 0.77 (0.73,0.8) |
| Saint Kitts and Nevis | 709 (518,913) | 1442 (1057,1897) | 103.39 | 10775.31 (9945.16,11702.69) | 11978.48(11325.33,12668.98) | 0.33 (0.32,0.35) |
| Saint Lucia | 1266 (932,1641) | 4513 (3316,5835) | 256.48 | 8675.28 (8188.30,9202.31) | 10921.61(10604.18,11247.64) | 0.65 (0.56,0.73) |
| Saint Vincent and the Grenadines | 1094 (812,1418) | 2611 (1919,3401) | 138.67 | 9093.94 (8550.26,9684.62) | 10489.96(10086.05,10915.00) | 0.44 (0.42,0.47) |
| Samoa | 1109 (801,1459) | 2491 (1808,3238) | 124.62 | 8158.79 (7664.27,8695.13) | 10633.38(10208.79,11079.62) | 1.05 (0.96,1.14) |
| San Marino | 596 (436,774) | 1254 (918,1609) | 110.40 | 9883.75 (9100.05,10720.35) | 10107.58(9539.11,10702.76) | 0.05 (0.04,0.07) |
| Sao Tome and Principe | 772 (564,1018) | 1916 (1391,2513) | 148.19 | 7208.48 (6698.69,7757.01) | 11152.80(10637.76,11703.42) | 1.53 (1.44,1.62) |
| Saudi Arabia | 64830 (47242,85622) | 262148 (190266,345388) | 304.36 | 7695.80 (7634.64,7757.52) | 9658.53(9614.90,9702.46) | -0.2 (-0.54,0.13) |
| Senegal | 29302 (20976,38942) | 93592 (68328,123282) | 219.40 | 5864.11 (5793.51,5936.20) | 7673.83(7622.99,7725.08) | 0.82 (0.79,0.85) |
| Serbia | 207411 (151131,271393) | 371734 (275050,478519) | 79.23 | 10582.11 (10531.89,10632.85) | 12894.46(12852.54,12936.49) | 0.74 (0.71,0.77) |
| Seychelles | 802 (584,1045) | 2273 (1670,2954) | 183.42 | 8333.35 (7765.75,8933.33) | 11538.48(11058.26,12038.18) | 0.99 (0.86,1.11) |
| Sierra Leone | 15906 (11454,21080) | 40428 (28937,52828) | 154.17 | 4959.53 (4879.73,5040.91) | 7087.82(7015.94,7160.68) | 1.22 (1.12,1.32) |
| Singapore | 48821 (35932,63631) | 205406 (149910,264436) | 320.73 | 13978.61 (13851.63,14106.90) | 13715.64(13655.96,13775.54) | -0.07 (-0.08,-0.06) |
| Slovakia | 144538 (106430,188708) | 259584 (191380,337309) | 79.60 | 13987.36 (13913.98,14061.15) | 15602.73(15542.34,15663.30) | 0.35 (0.29,0.41) |
| Slovenia | 60431 (44169,78359) | 113982 (84973,146000) | 88.62 | 14176.41 (14061.53,14292.30) | 14959.81(14871.76,15048.28) | 0.14 (0.08,0.2) |
| Solomon Islands | 1384 (1003,1827) | 4554 (3307,5978) | 229.05 | 6800.53 (6355.81,7378.20) | 9078.60(8789.95,9392.16) | 0.99 (0.94,1.05) |
| Somalia | 16486 (11806,21938) | 48902 (35298,65907) | 196.63 | 5252.95 (5160.16,5348.90) | 5860.46(5788.75,5935.86) | 0.42 (0.38,0.47) |
| South Africa | 415530 (304658,536772) | 1061232 (778964,1370848) | 155.39 | 12753.23 (12713.93,12792.66) | 14151.22(14123.36,14179.17) | 0.37 (0.33,0.41) |
| South Sudan | 17406 (12453,23599) | 28064 (20300,37361) | 61.23 | 4458.65 (4387.03,4532.11) | 5033.25(4969.68,5097.77) | 0.48 (0.43,0.54) |
| Spain | 874494 (640147,1140414) | 1628498 (1199218,2092255) | 86.22 | 9079.80 (9060.65,9098.98) | 10001.41(9985.65,10017.20) | 0.35 (0.29,0.4) |
| Sri Lanka | 109145 (79372,144679) | 432182 (312843,570826) | 295.97 | 6506.99 (6465.63,6549.07) | 9185.46(9157.56,9213.45) | 1.08 (1.03,1.13) |
| Sudan | 64854 (46761,86309) | 203025 (148452,267894) | 213.05 | 4571.91 (4534.07,4610.30) | 6885.68(6854.85,6916.66) | 1.4 (1.28,1.52) |
| Suriname | 4713 (3456,6120) | 13111 (9687,16988) | 178.19 | 11237.16 (10913.85,11568.40) | 12112.15(11903.73,12323.66) | 0.29 (0.27,0.3) |
| Sweden | 242592 (170131,327247) | 353465 (247218,473681) | 45.70 | 9504.71 (9465.60,9543.96) | 9743.62(9710.28,9777.05) | 0.79 (-0.32,1.91) |
| Switzerland | 176882 (130812,230662) | 307159 (226294,396747) | 73.65 | 10102.96 (10055.28,10150.84) | 10059.62(10023.38,10095.97) | -0.02 (-0.03,-0.02) |
| Syrian Arab Republic | 50602 (36835,67542) | 193639 (141946,253360) | 282.67 | 6119.37 (6064.31,6174.87) | 8650.02(8608.43,8692.16) | 1.32 (1.23,1.41) |
| Taiwan (Province of China) | 207286 (149578,278651) | 770147 (560737,1011827) | 271.54 | 7912.73 (7876.03,7949.72) | 10204.68(10181.78,10227.62) | 0.81 (0.77,0.85) |
| Tajikistan | 52916 (38600,68709) | 133105 (97549,173479) | 151.54 | 11851.91 (11748.96,11955.80) | 13923.83(13841.77,14006.50) | 0.6 (0.46,0.75) |
| Thailand | 300745 (216734,397708) | 1542528 (1124917,2000365) | 412.90 | 5497.79 (5477.37,5518.29) | 8059.64(8046.89,8072.41) | 1.23 (1.2,1.26) |
| Timor-Leste | 1796 (1283,2438) | 11129 (8028,14594) | 519.65 | 4717.61 (4480.10,4969.63) | 8009.70(7853.11,8171.32) | 1.98 (1.82,2.14) |
| Togo | 10089 (7234,13425) | 44421 (31884,58740) | 340.29 | 5603.51 (5486.45,5724.40) | 7867.75(7785.95,7951.03) | 1.12 (1.04,1.21) |
| Tokelau | 19 (14,25) | 28 (20,36) | 47.37 | 8227.03 (4841.45,15304.57) | 10985.99(7290.91,16202.61) | 1.05 (1,1.09) |
| Tonga | 696 (506,913) | 1254 (918,1648) | 80.17 | 7776.85 (7187.48,8433.57) | 9506.16(8985.88,10051.95) | 0.64 (0.63,0.66) |
| Trinidad and Tobago | 14608 (10756,18870) | 40298 (29844,51998) | 175.86 | 10722.72 (10536.67,10918.46) | 11723.91(11608.99,11839.78) | 0.3 (0.28,0.32) |
| Tunisia | 58953 (43169,77759) | 219202 (161334,284802) | 271.83 | 7193.59 (7128.05,7260.70) | 9650.51(9609.66,9691.53) | 0.95 (0.89,1) |
| Turkey | 315943 (230961,420507) | 1387520 (1005368,1803563) | 339.17 | 5700.76 (5680.18,5721.41) | 8642.38(8627.87,8656.91) | 1.33 (0.87,1.8) |
| Turkmenistan | 41568 (30323,53725) | 122133 (90399,157154) | 193.81 | 13553.38 (13418.79,13689.37) | 18273.70(18166.11,18381.85) | 1.09 (0.96,1.22) |
| Tuvalu | 89 (64,116) | 192 (140,254) | 115.73 | 8194.58 (6271.35,12299.55) | 10980.76(9408.76,12993.96) | 0.97 (0.93,1.02) |
| Uganda | 47554 (34303,62931) | 137277 (98849,180873) | 188.68 | 4893.89 (4846.03,4942.66) | 6314.71(6279.41,6350.26) | 0.75 (0.71,0.79) |
| Ukraine | 1863331 (1388966,2388636) | 2402939 (1810245,3081729) | 28.96 | 15117.95 (15095.63,15140.30) | 17483.42(17461.10,17505.77) | 0.51 (0.48,0.55) |
| United Arab Emirates | 4313 (3140,5792) | 50726 (36438,67277) | 1076.12 | 8528.65 (8245.98,8827.67) | 8251.07(8104.29,8408.64) | -0.14 (-0.23,-0.04) |
| United Kingdom | 1653661 (1209529,2136885) | 2478755 (1820198,3180812) | 49.89 | 10654.44 (10637.92,10670.98) | 11259.54(11245.28,11273.82) | -0.37 (-0.76,0.03) |
| United Republic of Tanzania | 113084 (80688,149595) | 271404 (193996,354908) | 140.00 | 6731.18 (6687.11,6775.91) | 7037.24(7009.76,7064.85) | -0.39 (-0.59,-0.18) |
| United States of America | 7614598 (5610364,9766528) | 15443788 (11355709,19850168) | 102.82 | 14005.49 (13995.43,14015.56) | 15031.10(15023.56,15038.64) | 0.2 (-0.16,0.56) |
| United States Virgin Islands | 1456 (1058,1882) | 3949 (2892,5109) | 171.22 | 10915.63 (10317.70,11615.14) | 12046.70(11667.12,12439.35) | 0.31 (0.29,0.33) |
| Uruguay | 75695 (55281,98601) | 107624 (78988,139438) | 42.18 | 11124.70 (11045.21,11204.69) | 11404.65(11335.59,11474.05) | 0.09 (0.05,0.13) |
| Uzbekistan | 272914 (201419,355231) | 775692 (572642,1002372) | 184.23 | 14475.38 (14419.93,14531.06) | 17812.95(17770.52,17855.49) | 0.62 (0.54,0.71) |
| Vanuatu | 440 (314,583) | 1637 (1181,2155) | 272.05 | 4868.34 (4335.03,5623.56) | 6260.77(5920.44,6651.17) | 0.78 (0.75,0.82) |
| Venezuela (Bolivarian Republic of) | 152606 (111045,199260) | 580700 (425603,751456) | 280.52 | 10021.66 (9970.81,10072.78) | 11318.48(11289.07,11347.95) | -0.09 (-0.35,0.16) |
| Viet Nam | 310200 (222945,413618) | 1204405 (870203,1571792) | 288.27 | 4675.92 (4659.21,4692.69) | 7351.92(7338.41,7365.44) | 1.54 (1.5,1.57) |
| Yemen | 31465 (22603,42207) | 145517 (105843,191467) | 362.47 | 4375.81 (4321.50,4431.28) | 6869.81(6832.55,6907.35) | 1.82 (1.67,1.97) |
| Zambia | 29949 (21775,39381) | 74610 (53845,97934) | 149.12 | 7020.52 (6932.54,7111.34) | 7524.94(7465.71,7585.09) | 0.3 (0.13,0.47) |
| Zimbabwe | 36776 (26502,48293) | 66893 (48015,87708) | 81.89 | 5979.32 (5912.85,6046.98) | 6559.26(6501.51,6618.20) | 0.03 (-0.08,0.13) |
