# Supplementary material for: Global burden of hand osteoarthritis in adults aged ≥55 years: An underestimated and fastest-growing subtype, 1990 to 2021 with projections to 2050: an analysis of GBD 2021 data
Source: Medicine (Baltimore). 2026 Aug 21;105(34):e50098. doi: 10.1097/MD.0000000000050098 (PMC13502859; doi:10.1097/MD.0000000000050098)
Supplement: Supplementary file 9 [file medi-105-e50098-s009.docx]

**Table S6. The YLDs of HOA cases and rate in adults aged ≥55 years across 204 countries in 1990 and 2021, and the trends from 1990 to 2021.**

| **location** | **YLDs Cases** | | **Percentage change**  **(100%)** | **ASYR** | | **EAPC (95%CI)** |
| --- | --- | --- | --- | --- | --- | --- |
|  | **1990 (95% UI)** | **2021 (95% UI)** |  | **1990(95%CI)** | **2021(95%CI)** |  |
| Afghanistan | 1477 (678,2987) | 1876 (853,3803) | 27.01 | 130.01 (122.76,138.35) | 158.47(150.96,166.66) | 0.63 (0.52,0.73) |
| Albania | 714 (326,1475) | 2319 (1065,4729) | 224.79 | 216.95 (200.96,234.11) | 295.59(283.58,308.11) | 1.24 (1.16,1.32) |
| Algeria | 4276 (1921,8804) | 17269 (7881,35845) | 303.86 | 210.70 (202.61,221.67) | 292.36(287.71,297.30) | 1.04 (0.96,1.12) |
| American Samoa | 11 (5,24) | 29 (13,59) | 163.64 | 331.74 (154.83,1096.73) | 357.99(234.94,582.00) | 0.24 (0.24,0.25) |
| Andorra | 30 (14,62) | 84 (38,170) | 180.00 | 307.09 (200.71,795.85) | 315.86(251.50,392.20) | 0.1 (0.09,0.12) |
| Angola | 2128 (964,4451) | 7409 (3395,15307) | 248.17 | 368.46 (350.33,388.81) | 413.63(402.98,424.88) | 0.39 (0.38,0.41) |
| Antigua and Barbuda | 33 (15,68) | 71 (32,147) | 115.15 | 366.48 (250.43,522.27) | 386.31(300.27,500.87) | 0.14 (0.12,0.16) |
| Argentina | 20379 (9178,41374) | 35835 (16528,73410) | 75.84 | 369.01 (363.91,374.18) | 377.37(373.46,381.31) | 0.05 (0.04,0.06) |
| Armenia | 1803 (794,3749) | 4343 (1901,8975) | 140.88 | 393.79 (374.90,413.63) | 553.99(537.30,571.20) | 1.33 (1.23,1.43) |
| Australia | 11605 (5343,24154) | 27739 (12739,56185) | 139.03 | 348.65 (342.28,355.14) | 362.51(358.20,366.86) | 0.12 (0.11,0.13) |
| Austria | 6258 (2810,13056) | 9690 (4342,19623) | 54.84 | 311.18 (303.32,319.26) | 317.42(311.03,323.92) | 0.05 (0.04,0.06) |
| Azerbaijan | 4244 (1886,8743) | 10310 (4515,21125) | 142.93 | 518.06 (501.85,535.06) | 573.68(561.63,586.01) | 0.48 (0.31,0.66) |
| Bahamas | 89 (41,183) | 270 (123,551) | 203.37 | 371.25 (297.64,461.29) | 390.86(344.68,442.42) | 0.16 (0.14,0.18) |
| Bahrain | 78 (35,162) | 447 (202,904) | 473.08 | 304.55 (218.28,680.95) | 309.51(270.56,408.01) | 0.03 (0,0.05) |
| Bangladesh | 13747 (6271,28334) | 58075 (26339,119640) | 322.46 | 188.55 (185.34,191.85) | 254.36(252.24,256.50) | 1 (0.87,1.12) |
| Barbados | 175 (80,364) | 357 (163,736) | 104.00 | 357.15 (304.33,421.30) | 389.91(350.44,433.03) | 0.29 (0.25,0.32) |
| Belarus | 10518 (4866,21341) | 15978 (7423,32866) | 51.91 | 461.86 (452.93,470.96) | 555.54(546.87,564.32) | 0.71 (0.67,0.74) |
| Belgium | 8578 (3893,17719) | 12761 (5849,26213) | 48.76 | 321.47 (314.61,328.49) | 324.97(319.25,330.78) | 0.03 (0.02,0.04) |
| Belize | 41 (19,85) | 161 (73,330) | 292.68 | 267.32 (191.80,363.39) | 333.74(283.45,391.12) | 0.64 (0.59,0.69) |
| Benin | 557 (254,1146) | 2059 (933,4228) | 269.66 | 178.63 (163.87,194.82) | 262.84(251.23,275.06) | 1.25 (1.24,1.27) |
| Bermuda | 40 (18,82) | 94 (43,194) | 135.00 | 379.13 (269.40,536.41) | 395.84(319.58,485.68) | 0.15 (0.13,0.16) |
| Bhutan | 62 (28,126) | 219 (101,449) | 253.23 | 173.74 (129.32,244.97) | 223.73(194.91,256.61) | 0.76 (0.74,0.79) |
| Bolivia (Plurinational State of) | 1342 (608,2790) | 4818 (2196,9972) | 259.02 | 266.62 (251.93,282.65) | 320.74(311.50,330.32) | 0.59 (0.49,0.7) |
| Bosnia and Herzegovina | 2017 (912,4217) | 4505 (2054,9348) | 123.35 | 295.44 (281.76,310.12) | 407.59(395.70,419.81) | 1.21 (1.05,1.38) |
| Botswana | 235 (105,479) | 825 (377,1732) | 251.06 | 269.23 (230.84,328.23) | 361.99(336.17,390.68) | 0.87 (0.8,0.94) |
| Brazil | 45125 (20537,94209) | 164085 (75222,337729) | 263.62 | 318.14 (315.11,321.21) | 384.43(382.57,386.30) | 0.61 (0.59,0.64) |
| Brunei Darussalam | 62 (28,130) | 244 (110,504) | 293.55 | 409.55 (307.47,662.97) | 432.52(370.12,624.35) | 0.17 (0.15,0.18) |
| Bulgaria | 8709 (3974,17910) | 11666 (5302,24127) | 33.95 | 392.28 (381.89,404.09) | 472.94(464.13,481.99) | 0.67 (0.61,0.72) |
| Burkina Faso | 883 (397,1817) | 2095 (945,4271) | 137.26 | 131.07 (121.62,142.14) | 148.68(142.07,155.71) | 0.42 (0.38,0.45) |
| Burundi | 622 (285,1272) | 1346 (611,2735) | 116.40 | 173.90 (159.14,192.60) | 184.24(173.62,195.94) | 0.27 (0.21,0.33) |
| Cabo Verde | 71 (32,146) | 205 (94,427) | 188.73 | 177.45 (138.20,225.35) | 279.57(241.78,322.24) | 1.64 (1.59,1.7) |
| Cambodia | 884 (405,1805) | 4122 (1869,8515) | 366.29 | 130.11 (120.81,140.86) | 204.64(197.93,211.78) | 1.6 (1.55,1.66) |
| Cameroon | 1140 (505,2342) | 4462 (2022,9224) | 291.40 | 166.51 (156.31,177.79) | 235.58(228.22,243.33) | 1.07 (0.99,1.16) |
| Canada | 22044 (10115,46104) | 50607 (23059,105345) | 129.57 | 398.53 (393.26,403.85) | 403.22(399.69,406.78) | 0.04 (0.03,0.05) |
| Central African Republic | 361 (163,746) | 705 (318,1428) | 95.29 | 214.40 (187.49,249.43) | 225.49(204.35,255.70) | 0.08 (0.03,0.14) |
| Chad | 664 (300,1360) | 1387 (617,2852) | 108.89 | 148.61 (137.08,161.54) | 160.83(151.90,170.57) | 0.23 (0.17,0.29) |
| Chile | 5106 (2295,10515) | 15606 (7077,31803) | 205.64 | 308.91 (300.39,317.75) | 348.81(343.36,354.33) | 0.37 (0.35,0.39) |
| China | 196337 (87993,406852) | 885231 (405688,1832709) | 350.87 | 150.53 (149.74,151.34) | 241.47(240.95,241.98) | 1.53 (1.33,1.73) |
| Colombia | 8103 (3639,17071) | 34123 (15599,71122) | 321.12 | 290.41 (284.03,296.93) | 358.86(355.05,362.70) | 0.8 (0.75,0.84) |
| Comoros | 52 (24,106) | 186 (85,383) | 257.69 | 172.81 (123.95,277.68) | 242.07(207.45,284.20) | 1.23 (1.16,1.3) |
| Congo | 515 (234,1081) | 1425 (643,2919) | 176.70 | 308.24 (273.78,369.81) | 354.28(333.84,378.41) | 0.42 (0.36,0.48) |
| Cook Islands | 6 (3,13) | 17 (8,34) | 183.33 | 308.01 (109.98,1263.07) | 361.42(209.13,612.91) | 0.52 (0.49,0.54) |
| Costa Rica | 804 (363,1672) | 3302 (1513,6814) | 310.70 | 285.12 (265.72,305.68) | 347.43(335.64,359.54) | 0.66 (0.63,0.69) |
| Croatia | 4365 (1982,8832) | 6911 (3177,14176) | 58.33 | 407.37 (394.88,420.50) | 448.44(437.76,459.38) | 0.39 (0.37,0.42) |
| Cuba | 4869 (2201,10048) | 11278 (5143,23236) | 131.63 | 285.42 (277.19,294.40) | 330.77(324.66,336.97) | 0.47 (0.45,0.5) |
| Cyprus | 396 (177,818) | 1130 (513,2329) | 185.35 | 283.94 (233.28,1081.23) | 318.75(298.96,347.67) | 0.36 (0.31,0.42) |
| Czechia | 10901 (5002,22786) | 17554 (8136,36844) | 61.03 | 457.23 (448.48,466.21) | 483.87(476.55,491.28) | 0.16 (0.12,0.2) |
| C么te d'Ivoire | 1015 (467,2087) | 3890 (1770,7926) | 283.25 | 176.28 (164.02,190.20) | 235.60(227.66,243.95) | 0.88 (0.82,0.94) |
| Democratic People's Republic of Korea | 3882 (1722,8037) | 11342 (5173,23520) | 192.17 | 158.56 (153.30,164.05) | 208.63(204.71,212.65) | 0.87 (0.83,0.91) |
| Democratic Republic of the Congo | 5738 (2611,11917) | 13699 (6150,28035) | 138.74 | 234.46 (227.10,242.34) | 250.99(246.33,255.79) | 0.05 (-0.15,0.25) |
| Denmark | 5469 (2491,11358) | 6383 (2921,13108) | 16.71 | 404.82 (393.95,415.94) | 318.64(310.70,326.75) | -0.46 (-0.58,-0.34) |
| Djibouti | 37 (17,76) | 274 (124,570) | 640.54 | 195.69 (128.31,346.49) | 289.97(251.24,343.93) | 1.54 (1.4,1.67) |
| Dominica | 28 (13,58) | 49 (22,100) | 75.00 | 282.18 (186.51,430.66) | 332.42(244.91,445.35) | 0.53 (0.47,0.58) |
| Dominican Republic | 1873 (850,3927) | 5895 (2708,12233) | 214.74 | 315.33 (300.85,330.67) | 356.23(347.19,365.48) | 0.37 (0.36,0.39) |
| Ecuador | 2575 (1160,5342) | 9543 (4347,19886) | 270.60 | 305.87 (294.03,318.39) | 347.89(340.91,355.01) | 0.43 (0.4,0.46) |
| Egypt | 7885 (3577,16128) | 24931 (11326,52055) | 216.18 | 189.31 (184.60,194.41) | 239.39(236.04,242.82) | 0.57 (0.51,0.64) |
| El Salvador | 1351 (613,2775) | 3549 (1616,7334) | 162.69 | 279.50 (264.75,294.88) | 342.61(331.37,354.14) | 0.73 (0.71,0.75) |
| Equatorial Guinea | 51 (23,105) | 266 (121,555) | 421.57 | 175.86 (124.25,297.22) | 356.81(312.47,412.42) | 2.74 (2.56,2.92) |
| Eritrea | 242 (110,488) | 867 (394,1779) | 258.26 | 163.14 (135.74,210.62) | 219.69(202.37,243.23) | 1 (0.98,1.03) |
| Estonia | 1796 (823,3703) | 2668 (1245,5534) | 48.55 | 503.98 (480.57,528.96) | 586.82(564.20,610.19) | 0.48 (0.41,0.56) |
| Eswatini | 95 (43,196) | 276 (126,564) | 190.53 | 226.77 (180.32,296.35) | 326.15(280.92,416.05) | 1.18 (1.14,1.22) |
| Ethiopia | 5941 (2678,12377) | 24659 (11215,51060) | 315.06 | 205.69 (198.52,213.86) | 374.84(370.03,379.74) | 2.29 (2.2,2.39) |
| Fiji | 137 (62,281) | 435 (197,898) | 217.52 | 262.52 (216.21,326.28) | 340.29(304.91,392.14) | 0.9 (0.86,0.95) |
| Finland | 3648 (1679,7524) | 6716 (3081,13884) | 84.10 | 300.47 (290.70,310.55) | 316.03(308.25,323.97) | 0.16 (0.14,0.17) |
| France | 44956 (20563,93170) | 75082 (34272,155628) | 67.01 | 315.62 (312.64,318.62) | 322.89(320.51,325.28) | 0.07 (0.06,0.08) |
| Gabon | 234 (106,485) | 543 (245,1134) | 132.05 | 249.53 (217.11,289.99) | 326.82(297.86,360.52) | 0.82 (0.78,0.87) |
| Gambia | 99 (45,204) | 421 (189,868) | 325.25 | 191.16 (151.85,261.12) | 284.26(256.85,316.44) | 1.35 (1.32,1.39) |
| Georgia | 4719 (2133,9556) | 5351 (2439,10832) | 13.39 | 437.81 (425.19,450.85) | 502.14(488.62,515.96) | 0.32 (0.14,0.5) |
| Germany | 69195 (31237,140731) | 105835 (47904,213624) | 52.95 | 322.13 (319.70,324.58) | 323.52(321.52,325.52) | -0.14 (-0.2,-0.09) |
| Ghana | 3381 (1496,7005) | 8098 (3672,16811) | 139.51 | 349.90 (337.23,363.47) | 314.60(307.32,322.16) | -0.17 (-0.6,0.25) |
| Greece | 6646 (3044,13608) | 11426 (5256,23491) | 71.92 | 248.73 (242.70,254.96) | 292.61(287.04,298.28) | 2.05 (1.47,2.64) |
| Greenland | 19 (8,38) | 49 (22,103) | 157.89 | 363.37 (199.41,1402.01) | 387.36(278.80,566.14) | 0.21 (0.19,0.22) |
| Grenada | 37 (17,77) | 68 (31,139) | 83.78 | 298.10 (209.10,414.49) | 353.73(271.40,462.80) | 0.52 (0.45,0.59) |
| Guam | 40 (18,83) | 140 (64,290) | 250.00 | 329.02 (218.66,882.19) | 373.99(313.89,443.19) | 0.41 (0.38,0.44) |
| Guatemala | 1318 (601,2712) | 5169 (2358,10716) | 292.19 | 241.85 (227.64,259.65) | 286.73(278.78,295.21) | 0.53 (0.52,0.55) |
| Guinea | 763 (348,1594) | 1540 (704,3159) | 101.83 | 142.98 (132.70,154.28) | 177.12(168.15,186.62) | 0.65 (0.61,0.69) |
| Guinea-Bissau | 96 (44,196) | 217 (98,442) | 126.04 | 162.02 (126.31,231.01) | 208.02(176.71,257.67) | 0.79 (0.74,0.84) |
| Guyana | 163 (75,336) | 367 (169,767) | 125.15 | 275.39 (233.88,327.09) | 341.13(305.89,380.86) | 0.69 (0.66,0.73) |
| Haiti | 912 (423,1846) | 2646 (1182,5439) | 190.13 | 182.85 (169.72,198.36) | 238.06(228.37,248.49) | 0.89 (0.88,0.91) |
| Honduras | 812 (367,1663) | 2995 (1365,6098) | 268.84 | 252.22 (234.42,273.66) | 292.90(282.13,304.38) | 0.51 (0.47,0.56) |
| Hungary | 11189 (5108,23321) | 16274 (7373,33484) | 45.45 | 434.52 (426.25,443.06) | 490.81(483.11,498.62) | 0.34 (0.26,0.41) |
| Iceland | 181 (83,377) | 337 (154,699) | 86.19 | 373.13 (320.56,432.22) | 338.49(303.02,377.17) | -0.4 (-0.61,-0.18) |
| India | 150280 (69120,311488) | 598460 (274786,1238571) | 298.23 | 214.10 (212.90,215.31) | 308.50(307.70,309.31) | 1.22 (1.17,1.28) |
| Indonesia | 31051 (14155,63993) | 117600 (53186,244944) | 278.73 | 210.67 (208.16,213.24) | 305.55(303.63,307.49) | 1.12 (1.06,1.18) |
| Iran (Islamic Republic of) | 9985 (4558,20876) | 38645 (17650,80274) | 287.03 | 241.29 (235.84,246.89) | 308.45(305.33,311.59) | 0.92 (0.63,1.21) |
| Iraq | 2792 (1265,5829) | 9639 (4433,19908) | 245.24 | 225.54 (217.22,234.11) | 263.19(257.70,268.82) | 0.3 (0.23,0.37) |
| Ireland | 2044 (921,4227) | 4236 (1939,8729) | 107.24 | 293.90 (281.03,307.53) | 316.01(306.51,325.74) | 0.21 (0.18,0.25) |
| Israel | 2596 (1175,5363) | 6762 (3062,13984) | 160.48 | 314.97 (302.67,328.08) | 327.95(320.08,335.98) | -1.5 (-2.13,-0.87) |
| Italy | 52306 (23911,107974) | 86685 (39826,177650) | 65.73 | 338.24 (335.31,341.21) | 359.06(356.59,361.55) | 0.55 (0.28,0.83) |
| Jamaica | 883 (395,1799) | 1760 (802,3630) | 99.32 | 292.80 (273.69,312.97) | 331.77(316.31,347.81) | 0.39 (0.36,0.42) |
| Japan | 112926 (51764,233788) | 270876 (124974,553427) | 139.87 | 386.79 (384.52,389.08) | 464.43(462.50,466.37) | 1.35 (0.67,2.04) |
| Jordan | 526 (236,1089) | 3631 (1656,7480) | 590.30 | 261.85 (238.67,287.59) | 306.45(295.93,317.42) | 0.5 (0.46,0.54) |
| Kazakhstan | 10380 (4706,21312) | 20686 (9224,42564) | 99.29 | 510.33 (500.35,520.51) | 678.48(668.75,688.46) | 1.02 (0.94,1.1) |
| Kenya | 3232 (1472,6742) | 13035 (5927,27342) | 303.31 | 255.79 (246.59,265.58) | 372.91(366.02,380.00) | 1.27 (1.23,1.31) |
| Kiribati | 17 (8,35) | 38 (17,80) | 123.53 | 299.54 (161.15,747.38) | 329.73(219.05,652.67) | 0.2 (0.15,0.25) |
| Kuwait | 241 (109,508) | 1327 (608,2664) | 450.62 | 286.05 (248.85,328.52) | 304.26(286.90,322.64) | 0.29 (0.25,0.32) |
| Kyrgyzstan | 2389 (1062,4832) | 4639 (2071,9538) | 94.18 | 480.55 (460.98,501.20) | 578.89(561.28,597.13) | 0.84 (0.67,1.01) |
| Lao People's Democratic Republic | 461 (205,942) | 1532 (685,3153) | 232.32 | 146.14 (130.73,167.68) | 213.15(202.03,225.07) | 1.25 (1.16,1.33) |
| Latvia | 2915 (1327,5929) | 3839 (1772,7826) | 31.70 | 468.22 (451.19,485.96) | 564.14(546.08,582.67) | 0.69 (0.63,0.75) |
| Lebanon | 855 (387,1790) | 3135 (1427,6516) | 266.67 | 237.65 (221.41,255.19) | 314.86(303.86,326.17) | 0.94 (0.9,0.99) |
| Lesotho | 291 (134,602) | 551 (249,1134) | 89.35 | 216.99 (191.99,246.81) | 323.51(293.43,363.35) | 1.47 (1.36,1.59) |
| Liberia | 319 (146,652) | 721 (329,1494) | 126.02 | 174.47 (154.57,199.16) | 234.63(216.98,254.02) | 1.14 (1.07,1.22) |
| Libya | 758 (346,1578) | 2386 (1077,4903) | 214.78 | 253.00 (235.03,272.09) | 299.73(287.50,312.40) | 0.53 (0.45,0.62) |
| Lithuania | 3564 (1618,7325) | 5538 (2568,11352) | 55.39 | 453.22 (438.29,468.68) | 556.73(541.93,571.85) | 0.79 (0.73,0.84) |
| Luxembourg | 301 (134,617) | 584 (269,1188) | 94.02 | 319.33 (283.72,361.36) | 324.47(298.57,352.06) | 0.03 (0.01,0.05) |
| Madagascar | 1119 (510,2296) | 2682 (1199,5499) | 139.68 | 142.38 (133.63,151.91) | 163.85(156.74,171.55) | 0.43 (0.41,0.45) |
| Malawi | 941 (429,1935) | 2283 (1036,4694) | 142.61 | 160.99 (149.63,174.08) | 208.43(199.29,218.20) | 0.91 (0.86,0.97) |
| Malaysia | 3664 (1672,7438) | 15846 (7240,33092) | 332.48 | 256.53 (248.20,265.11) | 332.91(327.63,338.30) | 0.78 (0.71,0.85) |
| Maldives | 38 (17,79) | 181 (81,382) | 376.32 | 268.64 (175.99,658.20) | 352.48(301.46,411.70) | 0.72 (0.63,0.82) |
| Mali | 938 (422,1933) | 2698 (1214,5651) | 187.63 | 152.61 (141.83,165.00) | 199.24(191.00,208.49) | 0.89 (0.85,0.93) |
| Malta | 234 (104,481) | 544 (246,1124) | 132.48 | 323.36 (282.81,370.97) | 328.21(300.36,358.29) | 0.03 (0.01,0.05) |
| Marshall Islands | 8 (4,16) | 20 (9,42) | 150.00 | 318.53 (117.79,2406.50) | 366.92(193.81,1264.85) | 0.45 (0.42,0.49) |
| Mauritania | 288 (132,593) | 854 (392,1776) | 196.53 | 181.32 (159.62,210.64) | 247.12(230.42,265.14) | 1 (0.89,1.1) |
| Mauritius | 338 (154,708) | 1178 (536,2470) | 248.52 | 288.60 (257.29,325.39) | 357.00(336.62,378.42) | 0.69 (0.67,0.72) |
| Mexico | 21968 (10021,45460) | 92097 (41962,189854) | 319.23 | 325.81 (321.37,330.35) | 435.36(432.53,438.19) | 1.13 (1.03,1.22) |
| Micronesia (Federated States of) | 21 (9,42) | 41 (19,86) | 95.24 | 267.93 (159.20,601.49) | 340.84(233.34,552.46) | 0.82 (0.77,0.87) |
| Monaco | 38 (17,79) | 53 (24,108) | 39.47 | 322.33 (224.29,455.57) | 324.25(240.89,429.42) | 0.02 (0.01,0.03) |
| Mongolia | 694 (309,1437) | 2450 (1083,5031) | 253.03 | 414.05 (382.90,448.79) | 660.66(632.11,693.51) | 1.73 (1.63,1.83) |
| Montenegro | 446 (204,944) | 807 (370,1658) | 80.94 | 420.54 (381.84,462.67) | 464.02(431.79,500.34) | 0.41 (0.38,0.45) |
| Morocco | 5608 (2512,11679) | 14719 (6783,30121) | 162.46 | 245.37 (238.89,252.06) | 254.91(250.69,259.22) | -0.07 (-0.21,0.06) |
| Mozambique | 1363 (620,2787) | 3266 (1480,6727) | 139.62 | 151.74 (142.90,161.80) | 199.36(191.87,207.30) | 0.9 (0.84,0.95) |
| Myanmar | 5935 (2711,12297) | 21142 (9608,43963) | 256.23 | 163.66 (159.12,168.47) | 263.36(259.70,267.06) | 1.72 (1.64,1.8) |
| Namibia | 223 (102,455) | 641 (289,1319) | 187.44 | 219.66 (188.17,260.33) | 301.22(276.83,329.27) | 0.93 (0.84,1.01) |
| Nauru | 2 (1,4) | 3 (1,6) | 50.00 | 290.16 (12.10,16217.06) | 353.06(56.19,4300.46) | 0.7 (0.63,0.77) |
| Nepal | 1976 (893,4008) | 7159 (3250,14538) | 262.30 | 139.99 (133.37,147.17) | 188.64(184.13,193.30) | 0.91 (0.86,0.96) |
| Netherlands | 11607 (5468,23688) | 19358 (8823,40474) | 66.78 | 343.74 (337.46,350.11) | 321.22(316.66,325.84) | -0.59 (-0.97,-0.21) |
| New Zealand | 2455 (1118,5093) | 5730 (2632,11854) | 133.40 | 366.30 (351.81,381.38) | 391.94(381.81,402.29) | 0.2 (0.18,0.22) |
| Nicaragua | 551 (251,1137) | 2374 (1080,4905) | 330.85 | 229.55 (210.54,250.08) | 298.67(286.65,311.30) | 0.87 (0.86,0.88) |
| Niger | 578 (260,1204) | 2035 (915,4178) | 252.08 | 144.59 (131.34,161.18) | 159.70(152.08,167.95) | 0.36 (0.32,0.41) |
| Nigeria | 15641 (7139,32223) | 40396 (18389,84595) | 158.27 | 228.62 (224.89,232.44) | 290.43(287.47,293.43) | 1.02 (0.86,1.17) |
| Niue | 1 (1,2) | 1 (1,3) | 0.00 | 311.87 (11.68,1880.71) | 361.32(20.72,2692.04) | 0.51 (0.49,0.54) |
| North Macedonia | 1075 (487,2208) | 2389 (1081,4911) | 122.23 | 339.30 (318.88,360.83) | 409.58(388.30,458.08) | 0.73 (0.68,0.78) |
| Northern Mariana Islands | 7 (3,16) | 32 (15,65) | 357.14 | 334.50 (110.52,3222.41) | 358.69(231.70,620.40) | 0.17 (0.15,0.19) |
| Norway | 4174 (1928,8617) | 6330 (2923,13085) | 51.65 | 362.70 (351.41,374.30) | 375.04(365.71,384.57) | 0.37 (0.02,0.73) |
| Oman | 258 (117,533) | 900 (411,1857) | 248.84 | 269.29 (236.15,308.32) | 314.80(292.52,339.69) | 0.46 (0.43,0.5) |
| Pakistan | 20072 (9175,41242) | 56492 (26005,118007) | 181.45 | 227.42 (224.17,230.72) | 304.03(301.40,306.69) | 0.89 (0.84,0.93) |
| Palau | 5 (2,10) | 14 (6,29) | 180.00 | 311.15 (87.22,1972.58) | 358.07(177.16,1369.94) | 0.45 (0.42,0.48) |
| Palestine | 300 (137,618) | 1182 (543,2485) | 294.00 | 214.41 (190.27,242.17) | 294.95(277.28,314.42) | 1.02 (0.98,1.07) |
| Panama | 684 (310,1402) | 2563 (1160,5224) | 274.71 | 285.33 (264.27,307.80) | 344.48(331.25,358.11) | 0.58 (0.56,0.6) |
| Papua New Guinea | 424 (191,872) | 1536 (694,3180) | 262.26 | 159.03 (140.42,183.71) | 206.82(195.34,219.37) | 0.86 (0.84,0.89) |
| Paraguay | 1185 (538,2477) | 3188 (1443,6632) | 169.03 | 331.47 (312.78,351.08) | 328.75(317.36,340.48) | -0.07 (-0.28,0.14) |
| Peru | 6377 (2888,13323) | 20467 (9271,41729) | 220.95 | 331.73 (323.59,340.04) | 368.17(363.13,373.25) | 0.2 (0.08,0.33) |
| Philippines | 11902 (5455,24809) | 44117 (20237,92096) | 270.67 | 265.12 (259.22,271.78) | 335.49(332.25,338.76) | 0.61 (0.54,0.67) |
| Poland | 31078 (14218,64296) | 63001 (28962,130151) | 102.72 | 410.08 (405.45,414.79) | 503.58(499.60,507.60) | 0.72 (0.66,0.78) |
| Portugal | 6900 (3121,14324) | 12662 (5778,25719) | 83.51 | 282.55 (275.76,289.59) | 308.93(303.41,314.54) | 0.95 (0.54,1.37) |
| Puerto Rico | 2224 (997,4584) | 4783 (2195,9894) | 115.06 | 363.84 (348.67,379.82) | 390.08(378.77,401.67) | 0.23 (0.22,0.23) |
| Qatar | 39 (18,82) | 385 (174,781) | 887.18 | 276.75 (179.29,616.74) | 295.79(257.32,348.00) | 0.17 (0.13,0.2) |
| Republic of Korea | 16995 (7660,34936) | 69105 (30955,143654) | 306.62 | 362.85 (356.88,369.18) | 415.25(412.15,418.37) | 0.41 (0.23,0.58) |
| Republic of Moldova | 2674 (1210,5471) | 4697 (2162,9645) | 75.65 | 357.53 (343.38,372.80) | 444.30(431.61,457.29) | 0.93 (0.79,1.07) |
| Romania | 16537 (7505,34804) | 24422 (11010,51477) | 47.68 | 340.16 (334.60,345.98) | 387.30(382.32,392.35) | 0.53 (0.48,0.57) |
| Russian Federation | 192813 (89006,399671) | 260104 (119578,541913) | 34.90 | 623.19 (620.31,626.08) | 609.65(607.26,612.04) | 0.21 (0.12,0.31) |
| Rwanda | 599 (267,1211) | 1705 (760,3460) | 184.64 | 140.36 (127.61,156.08) | 175.90(166.90,185.71) | 0.79 (0.75,0.83) |
| Saint Kitts and Nevis | 22 (10,47) | 46 (21,96) | 109.09 | 340.87 (207.51,605.66) | 377.00(269.26,526.68) | 0.32 (0.31,0.34) |
| Saint Lucia | 40 (18,81) | 142 (65,298) | 255.00 | 273.63 (193.25,404.68) | 343.54(289.22,406.87) | 0.64 (0.56,0.73) |
| Saint Vincent and the Grenadines | 35 (16,71) | 82 (38,169) | 134.29 | 288.08 (198.50,437.44) | 330.19(261.96,423.14) | 0.42 (0.4,0.45) |
| Samoa | 35 (16,72) | 79 (36,163) | 125.71 | 256.32 (175.59,389.65) | 333.02(261.75,429.24) | 1.04 (0.95,1.13) |
| San Marino | 19 (9,39) | 40 (18,82) | 110.53 | 314.81 (188.50,498.48) | 321.73(226.95,444.89) | 0.05 (0.04,0.06) |
| Sao Tome and Principe | 24 (11,50) | 61 (27,127) | 154.17 | 227.48 (145.07,354.96) | 351.50(265.54,481.40) | 1.53 (1.44,1.62) |
| Saudi Arabia | 2026 (929,4125) | 8257 (3702,16715) | 307.55 | 238.52 (227.91,249.69) | 299.41(291.86,307.26) | -0.22 (-0.56,0.12) |
| Senegal | 924 (421,1892) | 2950 (1358,6093) | 219.26 | 183.59 (171.35,197.46) | 240.32(231.43,249.63) | 0.83 (0.79,0.86) |
| Serbia | 6597 (2998,13668) | 11677 (5319,24094) | 77.00 | 334.23 (325.41,343.60) | 405.90(398.48,413.43) | 0.73 (0.7,0.77) |
| Seychelles | 25 (12,52) | 72 (33,149) | 188.00 | 264.43 (171.76,391.72) | 361.03(280.62,462.42) | 0.95 (0.82,1.07) |
| Sierra Leone | 500 (226,1031) | 1276 (583,2640) | 155.20 | 155.13 (141.35,170.60) | 222.41(209.89,235.95) | 1.24 (1.13,1.34) |
| Singapore | 1568 (707,3206) | 6621 (3017,13818) | 322.26 | 446.93 (424.54,470.67) | 441.27(430.63,452.13) | -0.05 (-0.06,-0.04) |
| Slovakia | 4540 (2070,9355) | 8161 (3774,16776) | 79.76 | 438.55 (425.66,451.87) | 490.73(480.06,501.58) | 0.36 (0.31,0.42) |
| Slovenia | 1893 (868,3891) | 3566 (1625,7302) | 88.38 | 443.52 (423.42,464.66) | 470.61(455.06,486.59) | 0.15 (0.1,0.2) |
| Solomon Islands | 44 (20,92) | 144 (65,298) | 227.27 | 214.67 (143.15,514.92) | 284.96(236.22,368.35) | 0.97 (0.92,1.03) |
| Somalia | 524 (235,1060) | 1551 (706,3166) | 195.99 | 164.67 (148.77,184.22) | 183.24(170.98,200.28) | 0.43 (0.38,0.48) |
| South Africa | 13088 (5991,27365) | 33081 (15054,67737) | 152.76 | 399.81 (392.89,406.85) | 438.50(433.63,443.46) | 0.34 (0.3,0.38) |
| South Sudan | 541 (242,1103) | 877 (395,1772) | 62.11 | 137.20 (125.00,151.43) | 155.32(144.41,167.22) | 0.5 (0.44,0.55) |
| Spain | 27733 (12342,57116) | 51275 (23087,105327) | 84.89 | 287.89 (284.49,291.33) | 317.36(314.55,320.19) | 0.34 (0.28,0.4) |
| Sri Lanka | 3463 (1582,7152) | 13544 (6162,28319) | 291.11 | 204.81 (197.59,212.82) | 286.50(281.61,291.48) | 1.05 (1,1.1) |
| Sudan | 2049 (927,4153) | 6415 (2898,13304) | 213.08 | 143.44 (136.86,150.62) | 216.13(210.71,221.68) | 1.4 (1.28,1.52) |
| Suriname | 150 (69,311) | 413 (188,855) | 175.33 | 357.13 (301.54,420.92) | 380.10(343.97,419.39) | 0.25 (0.24,0.27) |
| Sweden | 7662 (3464,15604) | 11119 (4985,22557) | 45.12 | 301.86 (294.91,308.96) | 309.20(303.27,315.24) | 0.79 (-0.32,1.91) |
| Switzerland | 5560 (2534,11389) | 9641 (4370,19874) | 73.40 | 318.93 (310.49,327.57) | 317.93(311.50,324.47) | -0.01 (-0.02,-0.01) |
| Syrian Arab Republic | 1604 (734,3284) | 6115 (2770,12451) | 281.23 | 192.60 (182.98,202.69) | 271.12(263.85,278.97) | 1.31 (1.22,1.4) |
| Taiwan (Province of China) | 6649 (3011,13957) | 24457 (11169,50576) | 267.83 | 251.81 (245.35,258.57) | 324.03(319.96,328.14) | 0.8 (0.75,0.84) |
| Tajikistan | 1690 (753,3498) | 4267 (1864,8918) | 152.49 | 377.18 (359.03,396.29) | 442.52(428.06,457.59) | 0.61 (0.46,0.76) |
| Thailand | 9547 (4359,19304) | 48739 (22079,99675) | 410.52 | 173.05 (169.46,176.72) | 254.26(252.01,256.54) | 1.25 (1.22,1.28) |
| Timor-Leste | 57 (26,118) | 350 (157,721) | 514.04 | 147.52 (108.38,203.55) | 250.71(223.80,283.30) | 2 (1.84,2.17) |
| Togo | 320 (145,653) | 1414 (629,2905) | 341.88 | 176.09 (155.99,200.51) | 247.93(233.71,263.73) | 1.14 (1.05,1.22) |
| Tokelau | 1 (0,1) | 1 (0,2) | 0.00 | 259.46 (0.51,6056.77) | 343.64(5.44,2738.90) | 1.03 (0.98,1.08) |
| Tonga | 22 (10,46) | 39 (18,81) | 77.27 | 246.13 (151.56,431.11) | 298.56(212.56,412.00) | 0.62 (0.6,0.64) |
| Trinidad and Tobago | 462 (211,961) | 1268 (583,2661) | 174.46 | 338.54 (306.32,383.09) | 368.16(348.05,389.23) | 0.28 (0.26,0.31) |
| Tunisia | 1874 (858,3899) | 6874 (3148,14405) | 266.81 | 226.77 (215.38,239.93) | 301.40(294.23,308.73) | 0.92 (0.86,0.98) |
| Turkey | 10039 (4579,20284) | 43740 (19756,91174) | 335.70 | 179.78 (176.15,183.48) | 271.43(268.87,274.01) | 1.31 (0.84,1.79) |
| Turkmenistan | 1327 (590,2684) | 3909 (1714,7903) | 194.57 | 430.41 (406.77,455.48) | 581.36(562.36,600.93) | 1.11 (0.98,1.24) |
| Tuvalu | 3 (1,6) | 6 (3,13) | 100.00 | 258.80 (31.05,3970.50) | 345.21(120.16,1275.05) | 0.97 (0.93,1.02) |
| Uganda | 1500 (686,3035) | 4352 (1956,8954) | 190.13 | 153.00 (144.72,162.29) | 198.48(192.30,204.92) | 0.78 (0.75,0.82) |
| Ukraine | 58480 (26446,121804) | 75284 (34533,158114) | 28.73 | 473.37 (469.44,477.35) | 548.02(544.07,551.99) | 0.54 (0.5,0.57) |
| United Arab Emirates | 136 (61,285) | 1623 (731,3363) | 1093.38 | 266.44 (218.85,334.13) | 258.05(233.16,297.94) | -0.13 (-0.22,-0.04) |
| United Kingdom | 52269 (24052,108854) | 77909 (35940,160859) | 49.05 | 337.68 (334.74,340.64) | 355.84(353.30,358.39) | -0.38 (-0.77,0.02) |
| United Republic of Tanzania | 3576 (1617,7405) | 8586 (3923,17450) | 140.10 | 210.76 (203.11,219.13) | 221.00(216.17,225.95) | -0.37 (-0.57,-0.16) |
| United States of America | 238774 (109735,493476) | 477385 (220732,981165) | 99.93 | 440.37 (438.58,442.16) | 465.79(464.46,467.12) | 0.16 (-0.21,0.52) |
| United States Virgin Islands | 47 (21,96) | 124 (57,256) | 163.83 | 346.98 (248.06,606.95) | 378.91(314.11,457.91) | 0.28 (0.25,0.3) |
| Uruguay | 2403 (1091,4976) | 3384 (1539,6945) | 40.82 | 353.14 (339.09,367.70) | 360.31(348.09,372.87) | 0.08 (0.04,0.11) |
| Uzbekistan | 8691 (3934,17843) | 24724 (10905,50518) | 184.48 | 459.87 (450.04,469.93) | 563.78(556.28,571.39) | 0.62 (0.53,0.7) |
| Vanuatu | 14 (6,29) | 52 (23,107) | 271.43 | 153.62 (73.07,618.08) | 196.69(140.88,329.38) | 0.78 (0.74,0.81) |
| Venezuela (Bolivarian Republic of) | 4851 (2198,9910) | 18376 (8336,38059) | 278.81 | 317.29 (308.32,326.55) | 357.17(351.97,362.43) | -0.11 (-0.37,0.15) |
| Viet Nam | 9871 (4461,20550) | 38474 (17256,79368) | 289.77 | 147.90 (144.95,150.91) | 232.98(230.59,235.39) | 1.55 (1.52,1.58) |
| Yemen | 989 (437,2003) | 4580 (2103,9293) | 363.09 | 136.12 (126.77,146.74) | 214.74(208.23,221.53) | 1.83 (1.68,1.98) |
| Zambia | 951 (432,1994) | 2354 (1079,4874) | 147.53 | 220.64 (205.43,239.15) | 235.31(225.02,246.59) | 0.3 (0.13,0.48) |
| Zimbabwe | 1172 (532,2402) | 2114 (969,4403) | 80.38 | 188.73 (177.18,201.56) | 205.23(195.24,216.55) | 0.01 (-0.1,0.11) |
