# Supplementary material for: Global burden of hand osteoarthritis in adults aged ≥55 years: An underestimated and fastest-growing subtype, 1990 to 2021 with projections to 2050: an analysis of GBD 2021 data
Source: Medicine (Baltimore). 2026 Aug 21;105(34):e50098. doi: 10.1097/MD.0000000000050098 (PMC13502859; doi:10.1097/MD.0000000000050098)
Supplement: Supplementary file 10 [file medi-105-e50098-s010.docx]

**Table S10. Final ARIMA model parameters, stationarity diagnostics, and residual autocorrelation tests for projected HOA and other osteoarthritis subtypes from 2022 to 2050.**

| **Cause** | **Measure** | **Age** | **Metric** | **ARIMA(p, d, q)** | **AIC** | **BIC** | **ADF *P* value** | **KPSS *P* value** | **Ljung_Box *P* value** |
| --- | --- | --- | --- | --- | --- | --- | --- | --- | --- |
| HOA | Incidence | ≥55years | number | ARIMA(1,2,0) | 634.02 | 636.82 | 0.0396 | >0.1 | 0.6407 |
| Knee OA | Incidence | ≥55years | number | ARIMA(1,2,0) | 703.27 | 706.07 | <0.01 | >0.1 | 0.9873 |
| Hip OA | Incidence | ≥55years | number | ARIMA(0,2,0) | 599.28 | 600.69 | 0.0348 | >0.1 | 0.9964 |
| Other OA | Incidence | ≥55years | number | ARIMA(0,2,2) | 554.77 | 558.98 | 0.0458 | >0.1 | 0.9599 |
| HOA | Incidence | ≥55years | rate | ARIMA(0,2,1) | 54.58 | 57.38 | 0.0385 | >0.1 | 0.7231 |
| Knee OA | Incidence | ≥55years | rate | ARIMA(2,1,1) | 101.18 | 108.35 | 0.0371 | >0.1 | 0.3308 |
| Hip OA | Incidence | ≥55years | rate | ARIMA(0,1,1) | 19.45 | 23.75 | 0.0163 | >0.1 | 0.9797 |
| Other OA | Incidence | ≥55years | rate | ARIMA(0,1,1) | -119.42 | -115.12 | 0.0398 | >0.1 | 0.6566 |
| HOA | prevalence | ≥55years | number | ARIMA(0,2,1) | 834.90 | 837.70 | 0.0290 | >0.1 | 0.9341 |
| Knee OA | prevalence | ≥55years | number | ARIMA(0,2,0) | 885.25 | 886.65 | 0.0343 | >0.1 | 0.9843 |
| Hip OA | prevalence | ≥55years | number | ARIMA(0,2,0) | 801.32 | 802.72 | <0.01 | >0.1 | 0.9784 |
| Other OA | prevalence | ≥55years | number | ARIMA(0,2,2) | 728.85 | 733.05 | 0.0446 | >0.1 | 0.7493 |
| HOA | prevalence | ≥55years | rate | ARIMA(1,1,3) | 273.39 | 280.56 | 0.0246 | >0.1 | 0.1150 |
| Knee OA | prevalence | ≥55years | rate | ARIMA(1,1,0) | 302.28 | 306.58 | <0.01 | >0.1 | 0.9733 |
| Hip OA | prevalence | ≥55years | rate | ARIMA(0,1,1) | 224.85 | 227.72 | <0.01 | >0.1 | 0.9511 |
| Other OA | prevalence | ≥55years | rate | ARIMA(0,1,1) | 84.56 | 88.86 | 0.0486 | >0.1 | 0.7655 |
| HOA | YLDs | ≥55years | number | ARIMA(0,2,2) | 628.18 | 632.38 | 0.0451 | >0.1 | 0.0729 |
| Knee OA | YLDs | ≥55years | number | ARIMA(0,2,0) | 677.17 | 678.57 | 0.0383 | >0.1 | 0.9871 |
| Hip OA | YLDs | ≥55years | number | ARIMA(0,2,0) | 593.69 | 595.09 | <0.01 | >0.1 | 0.9823 |
| Other OA | YLDs | ≥55years | number | ARIMA(1,2,0) | 533.70 | 536.50 | 0.0217 | >0.1 | 0.4135 |
| HOA | YLDs | ≥55years | rate | ARIMA(2,1,1) | 58.43 | 65.60 | 0.0474 | >0.1 | 0.0711 |
| Knee OA | YLDs | ≥55years | rate | ARIMA(1,1,0) | 85.79 | 90.09 | <0.01 | >0.1 | 0.9828 |
| Hip OA | YLDs | ≥55years | rate | ARIMA(0,1,1) | 10.34 | 13.20 | 0.0171 | >0.1 | 0.9524 |
| Other OA | YLDs | ≥55years | rate | ARIMA(0,1,2) | -103.90 | -98.17 | 0.0189 | >0.1 | 0.6006 |
| HOA | Incidence | All ages | rate | ARIMA(2,2,0) | -31.38 | -27.18 | 0.0475 | >0.1 | 0.2655 |
| HOA | prevalence | All ages | rate | ARIMA(3,1,0) | 174.99 | 180.73 | 0.0448 | >0.1 | 0.9281 |
| HOA | YLDs | All ages | rate | ARIMA(3,1,0) | -37.86 | -32.13 | <0.01 | >0.1 | 0.9503 |
